# Solvent Accessibility Promotes Rotamer Errors during Protein Modeling with Major Side-Chain Prediction Programs

**DOI:** 10.1021/acs.jcim.3c00134

**Published:** 2023-07-06

**Authors:** Tareq Hameduh, Michal Mokry, Andrew D. Miller, Zbynek Heger, Yazan Haddad

**Affiliations:** † Department of Chemistry and Biochemistry, Mendel University in Brno, Zemědělská 1665/1, CZ-613 00 Brno, Czech Republic; ‡ Veterinary Research Institute, Hudcova 296/70, CZ-621 00 Brno, Czech Republic; § KP Therapeutics (Europe) s.r.o., Purkyňova 649/127, CZ-612 00 Brno, Czech Republic

## Abstract

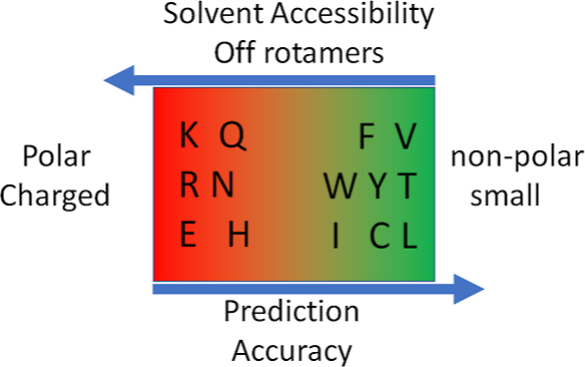

Side-chain rotamer prediction is one of the most critical late stages in protein 3D structure building. Highly advanced and specialized algorithms (e.g., FASPR, RASP, SCWRL4, and SCWRL4v) optimize this process by use of rotamer libraries, combinatorial searches, and scoring functions. We seek to identify the sources of key rotamer errors as a basis for correcting and improving the accuracy of protein modeling going forward. In order to evaluate the aforementioned programs, we process 2496 high-quality single-chained all-atom filtered 30% homology protein 3D structures and use discretized rotamer analysis to compare original with calculated structures. Among 513,024 filtered residue records, increased amino acid residue-dependent rotamer errors—associated in particular with polar and charged amino acid residues (ARG, LYS, and GLN)—clearly correlate with increased amino acid residue solvent accessibility and an increased residue tendency toward the adoption of non-canonical off rotamers which modeling programs struggle to predict accurately. Understanding the impact of solvent accessibility now appears key to improved side-chain prediction accuracies.

## Introduction

**Figure 1:**
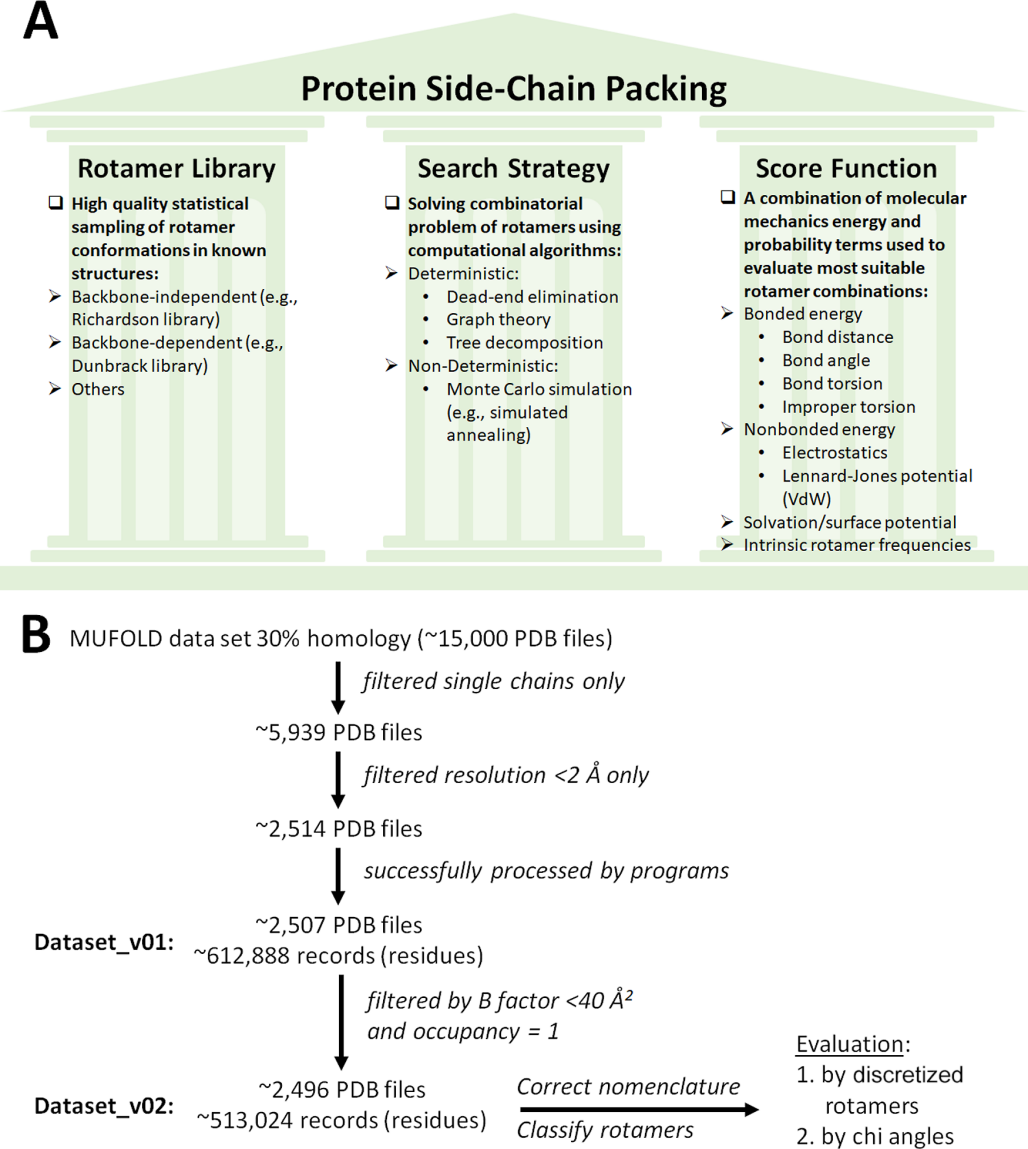
(A) Protein side-chain packing problem solving pillars. (B) Strategy to evaluate side-chain packing employed here.

Structural proteomes, holding deposited predicted protein 3D structures, are now emerging rapidly after recent leaps in the accuracy and quality of protein 3D structure predictions after nearly three decades of steady progress with homology modeling.^1−3^ Since 1994, Critical Assessment of Protein Structure Prediction (CASP) has been keeping a biannual record of progress in protein 3D structure prediction.^4^ Until CASP14 (in 2020), contact maps (amino acid structural contacts described in a 2D matrix) and deep learning were used as only “supporting steps” to homology modeling. However, the Alphafold2 program has used these strategies without recourse to homology modeling to produce highly accurate protein 3D structure predictions,^5,6^ so leading the way to future end-to-end prediction programs.^7−9^ Nevertheless, it is important to emphasize that previous homology modeling strategies are not now entirely redundant, indeed they still have adequate accuracy for use in the development of novel therapeutics and for investigations into cellular mechanisms at the molecular level. Accordingly, we have been seeking to understand where these previous state-of-the-art methods fell short, by taking a closer look at the sources of fine errors to shed the light on this problem. The protein side-chain packing problem is considered one of the most important late stages in homology modeling (which takes place after the step of building a backbone) with the objective of reaching a meaningful model with the least possible physical errors.^10^ The packing solution is supported by three main pillars (Figure 1A): (1) rotamer library: defined as statistical clustering of the sample space of observed side-chain conformations in known 3D structures. Rotamer libraries can be either backbone-dependent or -independent, where the former provides for higher accuracies of prediction. (2) Search strategy: solving the combinatorial problem of choosing the most fitting rotamer between different options using different mathematical models, e.g., Monte Carlo, graph theory-based approaches, tree-decomposition searches, or a combination of different models. (3) Scoring function: summation of different forms of energy of the protein calculated from natural frequencies or by molecular mechanics, which include contributions from protein bonded and non-bonded forces (e.g., van der Waals and electrostatic potentials) as well as solvent. In other words, side-chain prediction programs employ searching algorithms with different scoring functions to screen rotamer libraries, to find the most suitable packing with least “clashes”.^10−13^

Several cutting-edge programs are used to perform side-chain packaging with a decent level of accuracy, which in turn allows researchers to build models and reach reliable conclusions. Here, we attempted to evaluate and identify sources of errors in four of these algorithms (FASPR, RASP, SCWRL4, and SCWRL4v). Our choice of programs was based on their high predictive quality, their convenience in processing large protein data sets locally, their widespread use in scientific communities, and their computational efficiency.

The FASPR program (https://zhanggroup.org/FASPR/) was developed by the Zhang Lab (University of Michigan). This is an improved version of the two other programs (SCWRL4 and RASP) due to the combination of higher accuracy, speed, and determinacy during side-chain modeling. FASPR starts by reading the protein backbone coordinates and using the Dunbrack backbone-dependent rotamer library to construct the initial rotamer predictions. Then, the energy of the different rotamers is calculated with the exclusion of high-energy rotamers not in global minimum energy configurations. Next, residues with only one rotamer are retained, while those with multiple configurations are processed by different combinatorial search methods to select a preferred rotamer. Finally, a repacked structure model is generated.^11^

The RASP program (https://sourceforge.net/projects/raspv180/) was developed by the Jiang Lab (Chinese Academy of Sciences) as a faster successor to the CIS-RR program developed by the same laboratory. The first step of prediction starts with rotamer selection from the Dunbrack backbone-dependent rotamer library. This is followed by the calculation of the energies of different rotamer atoms and “clashes”, followed by combinatorial searches excluding low probability rotamers. Finally, protein structures are optimized by relaxing rotamers that are positioned relative to each other within 60% of the sum of their van der Waals radii (and so defined as “clashing”), while rotamers without “clashes” are retained.^14^

The SCWRL4 program (http://dunbrack.fccc.edu/lab/scwrl) was produced by the Dunbrack Lab (Fox Chase Cancer Center) using the Dunbrack backbone-dependent rotamer library. The program features speed, accuracy, and usability. After rotamer library data input and construction of coordinates, the calculation of energies is followed by graph computations.^15,16^ SCWRL4 uses a deterministic search method, which starts with a graph representation of amino acid interactions followed by combinatorial optimization (comprising dead-end elimination and tree decomposition).^10^

Recently, we employed a discretized rotamer analysis as an innovative tool for investigation of rotamer-related phenomena.^17,18^ Our choice for discretized classification of rotamers was based on the canonical plus/trans/minus method of Richardson Laboratory,^19,20^ while all rotamers found outside these classical structural ranges were classified as “off” rotamers (outside ±30°). These off rotamers should be intrinsically higher energy conformers than canonical rotamers, hence logically, less frequent in protein structures than related canonical rotamers. Our method has the benefit of avoiding the confusion in the literature where gauche nomenclature was used to describe both *g*^+^ and *g*^–^as +60° (and sometimes as −60°). For example, the mt rotamer of LEU represents any conformation of LEU side chains where the Chi1 angle is assigned as minus, i.e., −60° (belongs to interval −95 to −35°), and the Chi2 angle is assigned as trans, i.e., ±180° (belongs to interval +150 to −150°); hence, LEU side chains are said to be in a minus-trans rotameric state, shortened to mt. When the mode is neither plus (+60°), minus (−60°) nor trans (±180°), the angle is directly included as a shorthand (in the case of ARG, ASN, ASP, GLN, GLU, HIS, PHE, TRP, and TYR rotamers). PRO is special case of rotamers because it exists in cis and trans conformations depending on the amide nitrogen and Cα atoms. Here, we were mostly interested in the Cγ exo and Cγ endo rotamers that describe the Chi1 angle conformation related to the amide nitrogen and Cα atoms orientation to the ring. When the atoms are oriented to the inside the ring, the rotamer is denoted Cγ endo, and when they are oriented to the outside the ring, the rotamer is denoted Cγ exo.

We believe that discretized rotamer analysis provides a framework for biophysical interpretation of key rotamer errors for several reasons: first, the rotamer bins describe similar biophysical shapes that correlate with energy distributions. Second, combined classes provide for more effective descriptions of highly frequent rotamers than singular Chi angle classifications. Third, outliers (mostly within the off rotamer class) can be identified more easily. Hence, the aim of our work was to employ discretized rotamer analysis to evaluate the performance of four side-chain packing prediction algorithms (FASPR, RASP, SCWRL4, and SCWRL4v) in order to identify sources of errors at the amino acid residue level. In order to do this, we set out to compare the rotamer classes of high-quality residue-by-residue filtered protein 3D structures from the 30% low homology MUFOLD-DB data set with the same structures processed through the four programs mentioned above. The dominant theme emerging from our analysis was the apparent tendency for computational rotamer errors to be promoted when amino acid residues are exposed to higher solvent accessibility levels. Further analysis suggested that higher solvent accessibility levels also correlate with a selective preference for higher energy off rotamer classes in certain amino acids, consistent with solvent stabilization of these higher energy off rotamers. This work highlights the role of solvent accessibility in side-chain packing prediction and provides details concerning the types of errors involved. Given that most approaches to protein structure prediction go through either molecular docking (protein–protein and protein–ligand) or molecular dynamics where solvent accessibility plays a major role in interfacial interactions, then an accurate knowledge of the impact of solvent accessibility on side-chain packing will benefit these applications.

## Methods

### Protein Data Sets

The MUFOLD-DB data set with 30% homology^21^ was used here for testing the side-chain prediction software (website http://mufold.org/ was accessed on 5.6.2020 and data set is inactive at the time of submission). This data set has approx. 15,082 low homology protein PDB structures with contiguous chains where missing residues have been partially modeled by loop modeling. The data set was filtered after program testing according to the following criteria (Figure 1B): (1) to avoid errors in solvent accessibility calculations, only single-chained PDB files were retained. The original data set has separate chains per file; therefore, this step was vital to remove the multimers where part of the chain becomes solvent accessible during computation. The same PDB files downloaded from the RCSB Protein Data Bank (https://www.rcsb.org/) were used for comparison. (2) Only structures with resolution <2.0 Å were retained. (3) Torsional angles calculations were retained only when all atoms involved showed good B-factors (temperature) ≤40 Å^2^ and occupancies of 1. This criterion thus excludes residues with missing atoms or partially modeled loops mentioned above. Due to this rigorous quality filtering, only 2496 PDB structures were retained totaling 513,024 residues. Records for crystalized water, ions, and ligands are not included in the MUFOLD-DB collection and were not investigated or computed.

### Programs

All data processing and analysis were carried out in R language version 4.2.2 in RStudio version 2023.03.0 + 386. Functions of the dplyr and ggplot2 packages were used for general data analysis including summary and plots, respectively. Gradient graphs were created in Microsoft Excel for Microsoft 365 MSO (Version 2304) using conditional formatting “color scales”. Bash and Bat scripts written from loops in R were used to process PDB files using FASPR (https://github.com/xiaoqiah/FASPR last accessed in 11.11.2022), RASP v1.90 (https://sourceforge.net/projects/raspv180/ last accessed in 11.11.2022), and SCWRL4 (Scwrl4.0.4_64bit_2020; http://dunbrack.fccc.edu/lab/SCWRLlic last accessed in 11.11.2022) programs. The SCWRL4 program allows the option to search extended rotamer library by default. To disable this sub-rotamers sampling, we have used the option “-v” and denoted the results of processing as “SCWRL4v”.

### Nomenclature Correction

IUPAC-IUB commission rules were used to correct Chi angles in ASP, GLU, PHE, and TYR prior to side-chain evaluation.^22^ Briefly, for the residue ASP, if Chi1 angle was in the range −180° ≤ Chi1 ≤ −90° or +90° < Chi1 ≤ +180°, then it was rotated by 180°. For the residues GLU, PHE, and TYR, if Chi2 angle was in the range −180° ≤ Chi2 ≤ −90° or +90° < Chi2 ≤ +180°, then it was rotated by 180°. This rule was used to avoid biases in discretization due to periodicity in the branched amino acids. Unfortunately, we were unable to calculate the Chi5 angles for ARG, and thus, we have only accounted for the rotamer classes in the penultimate rotamer library for ARG. The Chi2 ranges of PHE and TYR described in a study by Lovell et al.^20^ and employed in this work are based on the tentative rules 2.3.2 and 4.3 about side-chain branching, as defined by IUPAC-IUB commission on biochemical nomenclature in 1969.^22^ Chi2 torsions can sometimes deviate from the rule due to mistakes of various sources (the force field used, improper documenting/reading of rotamer library, the optimization steps, the PDB writing algorithm, or a combination of these), in which case the Cδ and Cε atoms’ names are switched in the final written PDB records. An easy post hoc fix can be enabled by using the following rule: for PHE and TYR, if −180 ≤ Chi2 ≤ −90 or +90 < Chi2 ≤ +180 then “CD1” and “CD2”, and also “CE1” and “CE2”, atom names should be switched in the PDB records.

### Side-Chain Evaluation—Discretized Rotamer Analysis

Discretized classification of rotamers was carried out according to canonical plus/trans/minus method of Richardson Laboratory.^19,20^ Briefly, torsional angles were extracted in R using the torsion.pdb() function of the bio3d package by Grant Lab,^23^ and the classification of rotamers for each amino acid was carried out using our previously published code in R.^17,18^ Canonical rotamers were annotated according to their Chi angles, as previously described above. All rotamers found outside the canonical ranges (outside ±30°) were classified as “off” rotamers. Secondary structure information (coded by 8 categories: α-helix, β-bridge, extended β-strand, 3–10 helix, pi helix, turn, bend, and coil) and solvent accessibility score (ACC) were extracted using the dssp() function of the bio3d package in R. The mkdssp version 2.0.4 program used here was a C++ adaptation by Maarten L. Hekkelman of the original source code written by Kabsch and Sander (https://github.com/ecapriotti/lb1-2/blob/master/dssp/ last accessed in 11.11.2022). To describe the solvent accessibility, the static solvent exposure as number of water molecules in direct contact with certain residue was calculated as surface area in Å^2^ units. This solvent ACC can be converted to number of water molecules via division by a factor of 9.65 ≈ 10 or represented by a monomolecular layer of water molecules surrounding specific part of the protein.^24^

### Side-Chain Evaluation—Chi Angle Analysis

In order to study error distributions with amino acid side-chain structures, we also analyzed for Chi angle errors as a function of torsional angle deviations, wherein a correct angle prediction is identified within ±30° of the original torsional angle (for two angles *x* and *y*, the difference was calculated by the following formula: [difference = 180° – abs(abs(*x* – *y*) – 180°)] where abs() is the function to calculate the absolute value. The difference was then dichotomized into correct and error groups at 30° cut-off, denoted by 0 and 1, respectively). A decomposition of the error rates by amino acids at different levels of ACC values [all, zero, low (0–50 Å^2^), medium (50–100 Å^2^), and high (>100 Å^2^)] was as follows: (1) percentage of errors represents the sum of errors for given amino acid divided by sum of records per same amino acid (i.e., mathematically this was directly calculated as the mean of the dichotomized variable). (2) Percentage contribution of a given amino acid to errors was calculated from the sum of errors for that amino acid divided by sum of all error records for all amino acids.

### Statistical Analysis

Direct relationship between rotamer classes and solvent accessibility was investigated using analysis of variance (ANOVA) method via aov() function from the stats package in R. Tukey’s “honest significant difference” method was applied as post hoc using TukeyHSD() function from the stats package in R. For 963 ad-hoc combinations, the adjusted alpha level would be 0.05/963 = 0.000052, so ad-hoc *p*-value was adjusted accordingly. An adjusted *p*-value <0.05 was considered significant, and confidence interval (CI) was calculated at 95%.

## Results

### Quality-Filtering of Protein 3D Structures

Protein 3D structures analyzed here possessed a mean of 322.5 ± 167.6 amino acid residues and a range of 29–1287 residues. Of the 2496 structures involved, approx. 513,024 filtered residues were dominated by LEU (10.1%), ALA (9.4%), GLY (8.2%), and VAL (7.8%) while the rarest residues were CYS (1.3%), MET (1.4%), and TRP (1.6%) (Table 1). Theoretically, canonical rotamers of the small amino acids were anticipated to predominate due to their highly stable, low-energy conformational states, while higher energy off rotamer conformational states were expected to be scarce unless otherwise stabilized, for example, by interactions with solvent water molecules. Off rotamers were observed most frequently with ARG (44.1%), GLN (20.2%), LYS (14.0%), MET (12.5%), PRO (10.3%), and ASN (9.6%).

**Table 1:** Quality-Filtered Residues of 3D Structure Data Set Used in This Study Showing Rotamer Distribution

residue	canonical rotamers		off rotamers		all
ALA					48,429
ARG	11,829	55.9%	9347	44.1%	21,176
ASN	19,398	90.4%	2062	9.6%	21,460
ASP	29,155	97.6%	718	2.4%	29,873
CYS	6562	99.3%	44	0.7%	6606
GLN	12,926	79.8%	3264	20.2%	16,190
GLU	24,918	94.4%	1472	5.6%	26,390
GLY					42,216
HIS	12,059	95.6%	553	4.4%	12,612
ILE	30,857	98.6%	452	1.4%	31,309
LEU	49,462	95.5%	2334	4.5%	51,796
LYS	17,941	86.0%	2920	14.0%	20,861
MET	6335	87.5%	905	12.5%	7240
PHE	22,807	97.8%	521	2.2%	23,328
PRO	23,159	89.7%	2646	10.3%	25,805
SER	28,972	99.0%	283	1.0%	29,255
THR	29,334	99.4%	170	0.6%	29,504
TRP	7896	94.4%	469	5.6%	8365
TYR	20,155	97.6%	497	2.4%	20,652
VAL	39,791	99.6%	166	0.4%	39,957
**total**	**393,556**	**93.2%**	**28,823**	**6.8%**	**513,024**

### Evaluation of Side-Chain Predictions—Rotamer Errors

**Figure 2:**
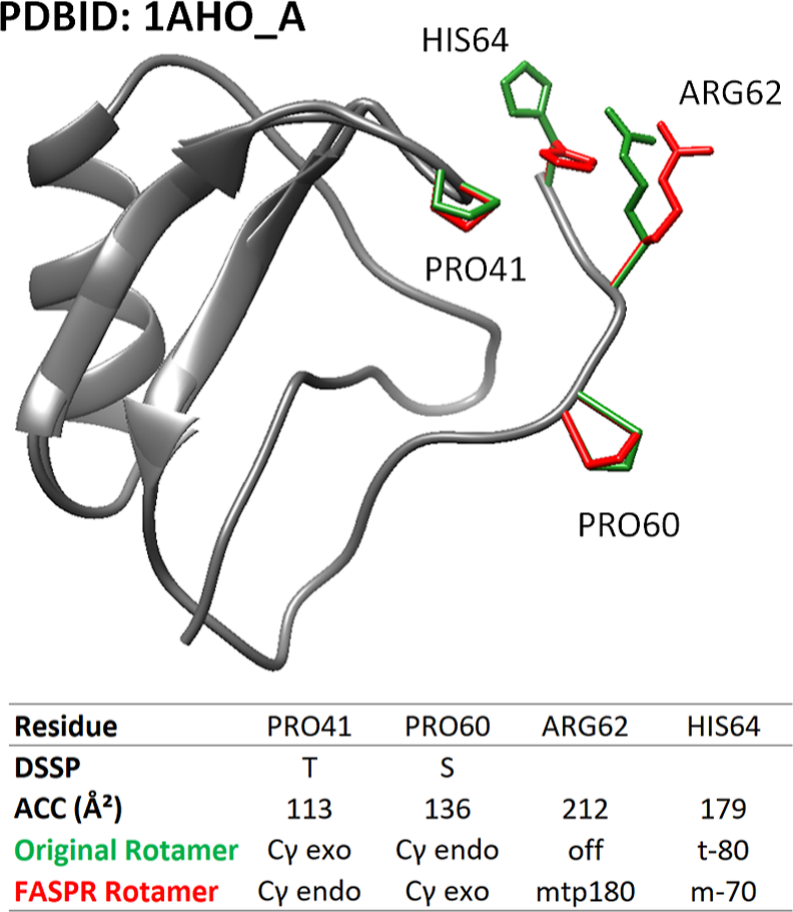
Illustration of side-chain prediction errors in scorpion toxin II protein (3D structure PDB ID 1AHO_A) after processing with the FASPR program. Errors are shown in the table below with their corresponding solvent ACC and secondary structures (DSSP code: T: turn, S: bend, _: coil). Original rotamer side chains are shown in green color while FASPR predicted side-chains are shown in red. The structure contained 64 residues, yet after filtering out bad B factors and uncertain occupancies, 57 residues were retained.

Each 3D structure was processed by the four algorithms which—to put it simply—remove original side chains and replace them with predicted ones. In order to evaluate key errors in prediction, we have employed two methods: in the first method, a discretized rotamer classification was used, residue-by-residue, for original and processed 3D structures. Thereafter, a logical comparison was employed to identify identical and non-identical rotamer classes (before and after processing). A representative example of key errors is shown in Figure 2. For long-chained amino acids, a deviation in one torsional angle was sufficient to be defined as a computational error in a discretized rotamer class. To study this effect, a secondary evaluation method was utilized to observe the deviation of each Chi angle independently of other Chi angles.

**Figure 3:**
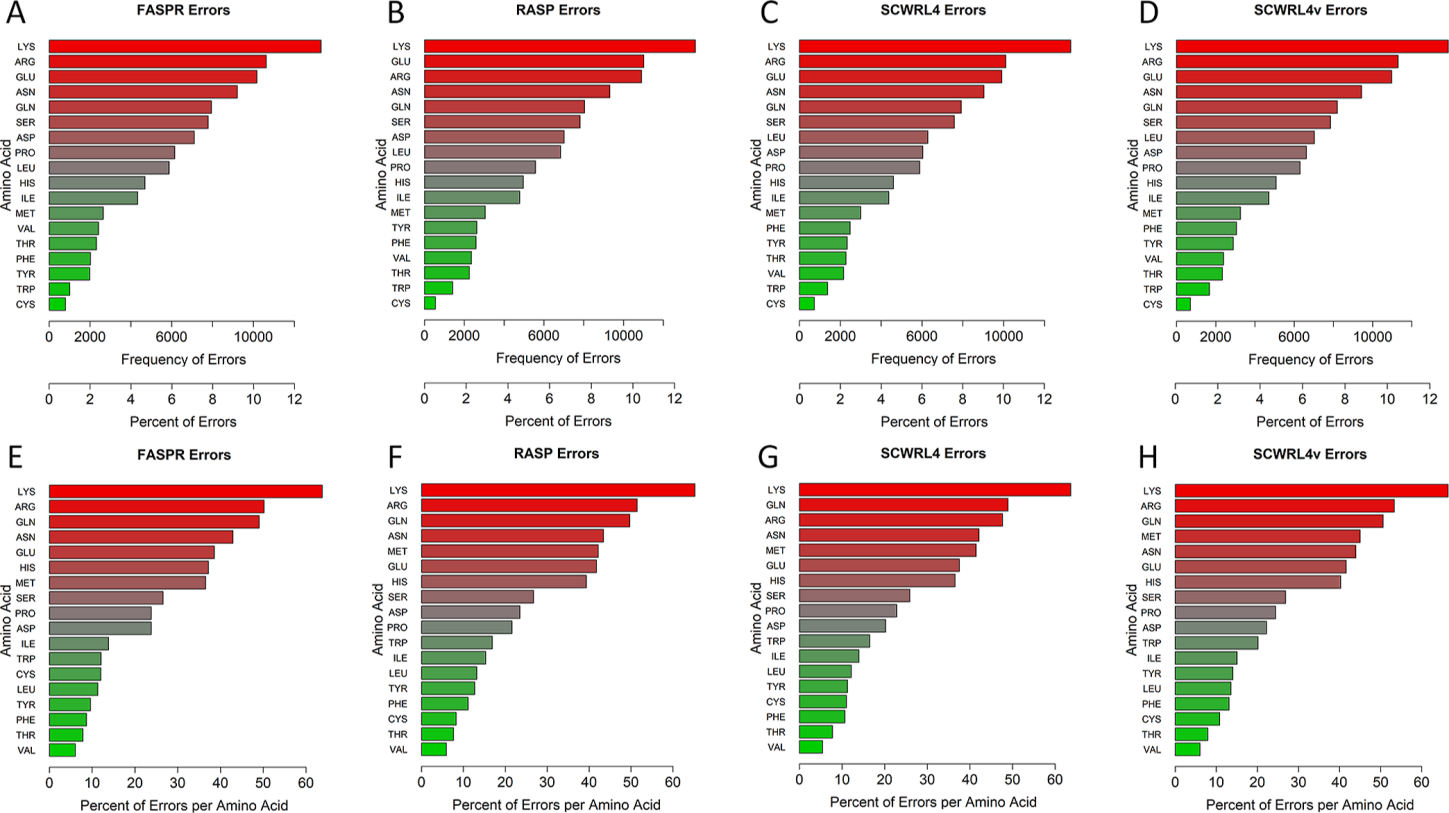
Variations in errors from amino acid residue side-chain prediction programs according to analysis by discretized rotamer classes. Frequency and percentage of amino acid residue side-chain prediction errors in structures processed by FASPR (A), RASP (B), SCWRL4 (C), and SCWRL4v (D). Percentage of amino acid residue side-chain prediction errors normalized to the frequency of residues in protein structures, as processed by FASPR (E), RASP (F), SCWRL4 (G), and SCWRL4v (H). SCWRL4v represents use of SCWRL4 program with fixed rotamer library search using -v option to disable sub-rotamers.

Overall, approx. FASPR generated 100,360 discretized rotamer assignment errors (23.8%), RASP generated 104,751 assignment errors (24.8%), and SCWRL4 produced 99,394 assignment errors (23.5%) while SCWRL4v yielded 107,712 errors (25.5%). The general similarity in the performance was clear between the four programs studied, so too we observed a general similarity between programs in the frequencies of rotamer prediction errors per amino acid residue type (Figure 3). The frequencies of rotamer prediction errors were found especially high for LYS (with 13,317; 13,617; 13,285; and 13,862 assignment errors found, respectively, post use of FASPR, RASP, SCWRL4, and SCWRL4v). Rotamer prediction errors were also high in the case of other charged or polar amino acid residues ARG, GLU, ASN, and GLN (Figure 3A–D). Rotamer prediction errors were least for TRP and CYS amino acid residues, which were also the least frequent amino acid residues in our protein data set in general (Table 1). When the percentage of rotamer prediction errors was determined per amino acid residue, the top ranked most error prone residues were LYS, ARG, GLN, ASN, MET, HIS, and GLU (Figure 3E–H). The least error prone residues were VAL, THR, CYS, PHE, and TYR.

**Figure 4:**
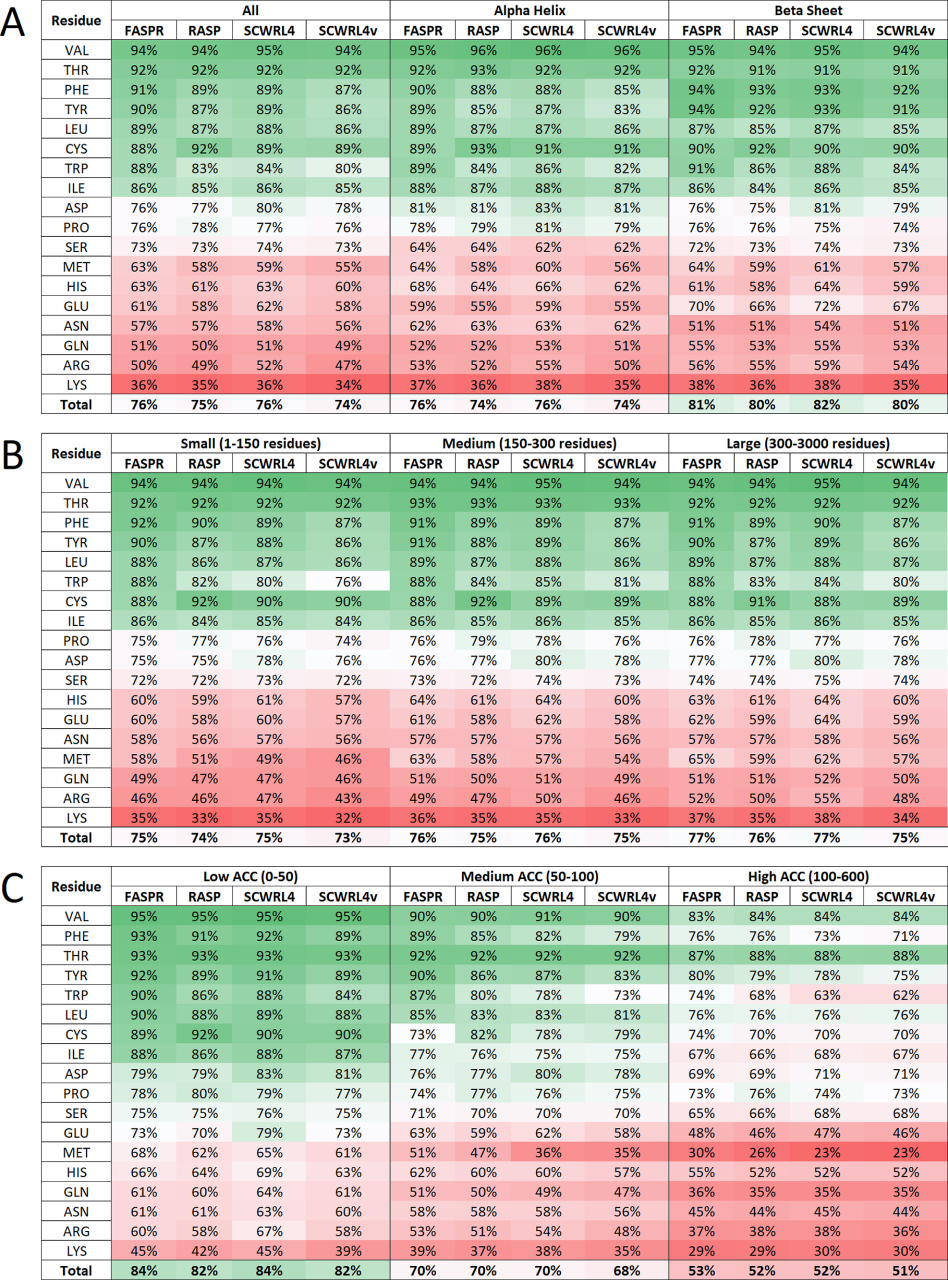
Accuracy of side-chain prediction programs as a function of amino acid residue and based on discretized rotamer classes. (A) Percentage accuracy of discretized rotamer predictions in all, alpha helix and beta sheet were categorized into three groupings of amino acid residues, following the use of FASPR, RASP, SCWRL4, and SCWRL4v. (B) Percentage accuracy of discretized rotamer predictions according to protein size. (C) Percentage accuracy of discretized rotamer predictions according to solvent accessibility (ACC). SCWRL4v represents use of SCWRL4 program with fixed rotamer library search using -v option to disable sub-rotamers.

When the amino acid frequency was taken into consideration, as shown in Figure 4A, then rates of prediction accuracy could be determined. These rates of prediction accuracy were found to vary between three distinct groupings of amino acids as follows. In the first grouping (VAL, THR, PHE, TYR, LEU, CYS, TRP, and ILE), rates of rotamer prediction accuracy were in the range 80–95% (reaching up to 96% for VAL in α-helix). In the second grouping (ASP, PRO, and SER), rates of rotamer prediction accuracy were in the medium range 73–80% (including SER in α-helix). Finally, in the third grouping (MET, HIS, GLU, ASN, GLN, ARG, and LYS), rates were only in the range 34–63%. Canonical rotamers were either similarly or better predicted with the exception of ARG wherein off rotamers were better predicted than canonical rotamers (Figure S1). Overall, the four programs exhibited similar patterns of prediction accuracy across most secondary structures, SCWRL4v being the least effective (Figures 4A and S1–S3). Interestingly, protein size was observed to have no clear influence on accuracy of discretized rotamer predictions (Figure 4B). On the other hand, dramatic correlations were observed when ACC was introduced as a scoring metric in data processing involving the four programs (Figure 4C). In this instance, rates of rotamer prediction accuracy declined from 82–84 to 68–70%, in moving from low to medium ACC, and to 51–53%, in moving from low to high ACC, with few exceptions. In general, prediction accuracies for VAL and THR residues deteriorated least with increasing ACC, while prediction accuracies for MET, GLN, LYS, and ARG deteriorated the most. Prediction accuracies for the first two groups of residues were generally well preserved (85–87% overall) at lower values of ACC (<50 Å^2^, i.e., when each residue is probably in contact with less than five molecules of water) (Figure S1). Therefore, we would suggest that rotamer prediction errors at higher solvent accessibility (beyond ACC >50 Å^2^) could be linked to amino acid side-chain contacts with five or more molecules of water.

### Evaluation of Side-Chain Predictions—Chi Angle Errors

**Figure 5:**
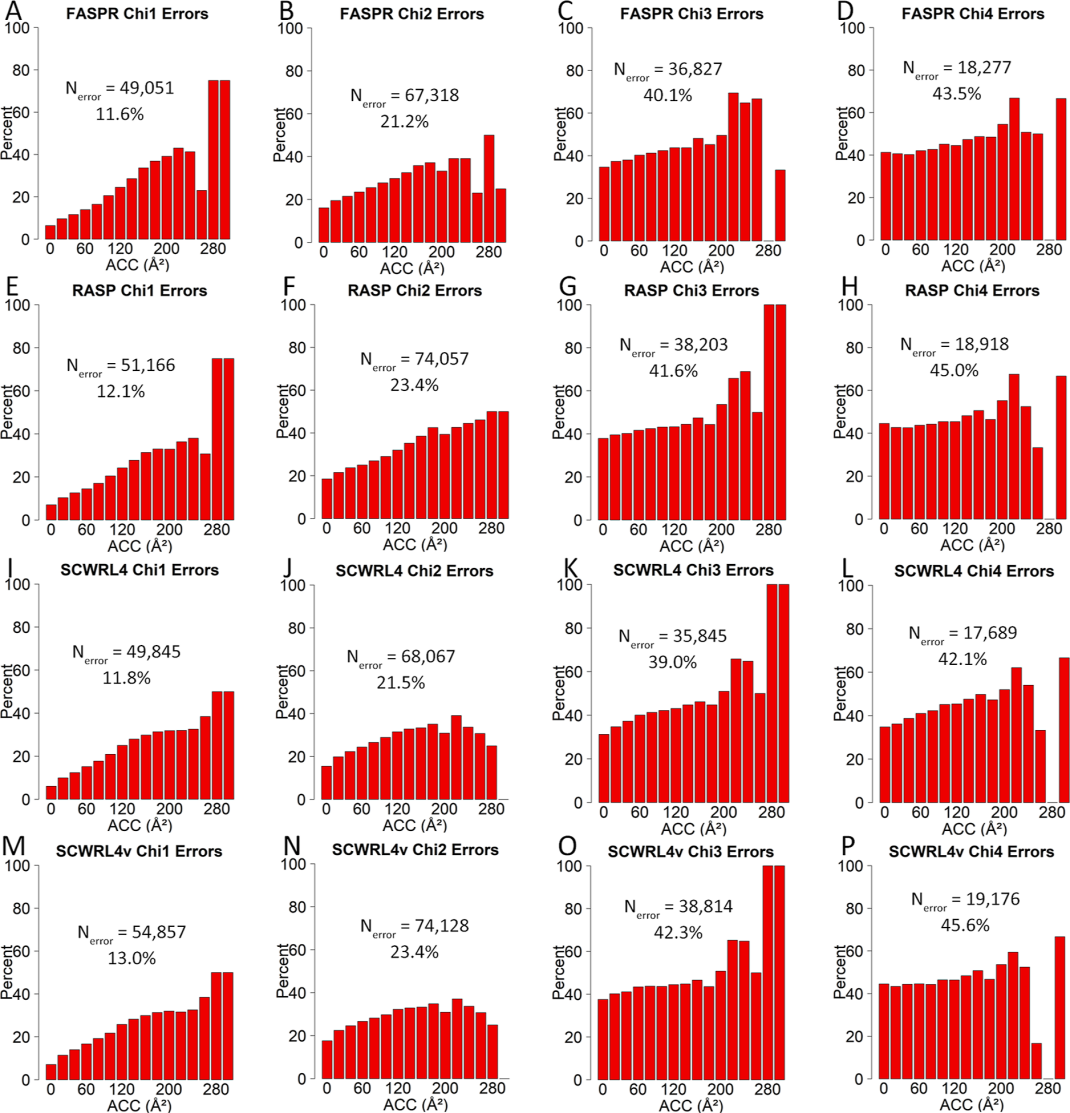
Errors in side-chain prediction programs according to solvent accessibility as a function of Chi angle deviation by 30°. Percent angular errors in torsional angles following analysis (A–D) with FASPR, (E–H) RASP, (I–L) SCWRL4, and (M–P) SCWRL4v. The *y*-axis represents percentage of errors in each bin divided by number of cases per bin. Percentages at ACC >220 Å^2^ may be based on few cases and may not be representative. Each bar is labeled by the lower limit of a 20 Å^2^ ACC interval divided from 0 to 320 Å^2^. SCWRL4v represents use of SCWRL4 program with fixed rotamer library search using -v option to disable sub-rotamers.

In studying Chi angles independently, the distribution of angular errors leading to discretized rotamer errors was highest when Chi2, Chi3, or Chi4 torsional angles were involved across the full solvent accessibility range (peaks around 6% of total cases in bins of 20 Å^2^ ACC) (Figure S4). In terms of percentages, the rate of angular errors increased from 11.6–13.0 to 21.2–23.4% going from Chi1 to Chi2 torsional angles, respectively, and up to 39.0–45.6% going to either Chi3 or Chi4 torsional angles, using each of the four programs (Figure 5). A closer look at the percentage of errors per each bin (20 Å^2^ ACC interval) revealed a steady increase in errors in the range of 0–200 Å^2^ ACC (Figure 5). This increase was very obvious in the Chi1 and Chi2 torsional angles. In contrast, percentages per total cases showed angular error peaks at 0 and 100 Å^2^ ACC; however, the former was not visible when Chi4 torsional angles were analyzed, and the latter was not properly visible when Chi1 and Chi2 torsional angles were analyzed (Figure S4). To understand the cause of error peaks at zero Å^2^ ACC, further analyses were performed calculating the percentage contribution of each amino acid residue to the observed angular errors. A decomposition of these angular errors according to amino acid residue showed evident correlations in errors and distances of Chi angles away from the polypeptide backbone (Figure S5). With few exceptions (e.g., LYS Chi4), angular errors at zero ACC were less frequent than errors at other defined ACC values, across all amino acid residues (Figure S5C). On the other hand, when the contribution of individual amino acid residues was taken into consideration, it was obvious that ILE, VAL, and LEU demonstrated the most Chi1 angular errors at zero ACC (Figure S6). PHE, LEU, and ILE exhibited the most Chi2 angular errors at zero ACC, while MET exhibited the most Chi3 errors at zero ACC (Figure S6). ARG, LYS, and GLU demonstrated significant Chi3 errors at all ACC values relative to zero ACC (Figure S6). Having noted these variations, amino acid residues in general exhibited a persistent increase in angular error rates with increasing ACC values whether charged and polar (Figure S7), or small and non-polar (Figure S8).

### Evaluation of Side-Chain Predictions—Role of Side-Chain Length

Amino acids were then grouped into four group categories according to their side-chain length (i.e., denoted by number of Chi angles): group I amino acids constituted residues with only the Chi1 torsional angle (CYS, SER, THR, and VAL), group II amino acids constituted residues with Chi1 and Chi2 torsional angles (ASN, ASP, HIS, ILE, LEU, PHE, PRO, TRP, and TYR), group III amino acids constituted residues with Chi1, Chi2, and Chi3 torsional angles (GLN, GLU, and MET), and group IV amino acids constituted residues with Chi1, Chi2, Chi3, and Chi4 torsional angles (LYS and ARG). The most frequent amino acid residue rotamer errors are shown (Tables 2–5).

**Table 2:** Top 10 Rotamer Errors Found in Group I Residues^a^

residue	rank	FASPR	count	RASP	count	SCWRL4	count	SCWRL4v	count
CYS	**1**	t to m	294	t to m	164	t to m	233	t to m	225
	**2**	p to m	240	m to t	125	p to m	198	p to m	197
	**3**	m to t	112	p to m	122	m to t	133	m to t	136
	**4**	m to p	51	m to p	35	m to p	55	m to p	50
	**5**	t to p	31	p to t	29	p to t	34	p to t	31
	**6**	off to m	26	t to p	26	t to p	32	t to p	30
	**7**	p to t	23	off to m	22	off to m	24	off to m	23
	**8**	off to p	9	off to p	11	off to t	11	off to t	11
	**9**	off to t	9	off to t	11	off to p	9	off to p	10
SER	**1**	m to p	2225	m to p	2392	m to p	2535	m to p	2384
	**2**	t to m	1383	m to t	1227	m to t	1203	p to m	1347
	**3**	p to m	1234	t to m	1164	p to m	1176	m to t	1307
	**4**	m to t	1128	p to m	1099	t to m	945	t to m	1071
	**5**	t to p	843	t to p	882	t to p	787	p to t	732
	**6**	p to t	685	p to t	775	p to t	651	t to p	731
	**7**	off to p	124	off to p	122	off to p	120	off to p	117
	**8**	off to t	95	off to t	103	off to t	96	off to t	97
	**9**	off to m	64	off to m	58	off to m	67	off to m	69
THR	**1**	m to p	661	p to m	789	p to m	738	p to m	788
	**2**	p to m	647	m to p	457	m to p	615	m to p	593
	**3**	t to p	488	t to p	310	t to p	338	t to p	331
	**4**	t to m	242	p to t	255	t to m	204	t to m	206
	**5**	off to p	93	t to m	171	p to t	161	p to t	172
	**6**	p to t	80	m to t	97	off to p	87	off to p	84
	**7**	off to m	70	off to m	80	off to m	75	m to t	78
	**8**	m to t	21	off to p	77	m to t	56	off to m	75
	**9**	off to t	7	off to t	13	off to t	8	off to t	11
VAL	**1**	m to t	869	p to t	607	m to t	592	m to t	602
	**2**	p to t	763	m to t	597	p to t	589	p to t	550
	**3**	m to p	184	t to p	331	p to m	241	t to p	360
	**4**	p to m	154	t to m	258	t to m	222	t to m	281
	**5**	t to p	154	p to m	252	t to p	205	p to m	250
	**6**	t to m	124	m to p	149	m to p	145	m to p	189
	**7**	off to t	114	off to t	104	off to t	96	off to t	105
	**8**	off to m	34	off to m	39	off to m	47	off to m	42
	**9**	off to p	18	off to p	23	off to p	23	off to p	19

a Errors described as “original to predicted”.

**Table 3:** Top 10 Rotamer Errors Found in Group II Residues^a^

residue	rank	FASPR	count	RASP	count	SCWRL4	count	SCWRL4v	count
ASN	**1**	m120° to m-20°	1747	m120° to m-20°	1393	m120° to m-20°	1402	m120° to m-20°	1366
	**2**	m-80° to m-20°	1378	m-80° to m-20°	886	m-80° to m-20°	830	m-80° to m-20°	871
	**3**	off to m-20°	981	off to m-20°	799	off to m-20°	826	off to m-20°	791
	**4**	t30° to m-20°	721	t30° to m-20°	606	t30° to t-20°	623	t30° to t-20°	625
	**5**	t30° to t-20°	691	t30° to t-20°	570	t30° to m-20°	509	m-20° to m-80°	515
	**6**	off to t-20°	374	t-20° to t30°	469	m-20° to m-80°	465	t30° to m-20°	514
	**7**	off to t30°	315	off to t30°	391	off to t30°	426	off to t30°	432
	**8**	t-20° to m-20°	290	m120° to m-80°	378	m120° to m-80°	415	m120° to m-80°	405
	**9**	t-20° to t30°	250	m-20° to m-80°	370	p-10° to p30°	379	t-20° to t30°	402
	**10**	p-10° to p30°	249	p-10° to p30°	367	t-20° to t30°	371	p-10° to p30°	373
ASP	**1**	p-10° to p30°	1252	t70° to m-20°	790	t70° to m-20°	655	t70° to m-20°	684
	**2**	t70° to m-20°	770	t70° to t0°	680	p-10° to p30°	598	p-10° to m-20°	599
	**3**	t0° to t70°	702	p-10° to p30°	656	p-10° to m-20°	567	p-10° to p30°	571
	**4**	t0° to m-20°	644	t0° to m-20°	609	t0° to t70°	549	p30° to p-10°	552
	**5**	p-10° to m-20°	631	m-20° to t0°	550	p30° to p-10°	545	m-20° to t0°	528
	**6**	off to m-20°	508	p30° to p-10°	547	off to m-20°	476	t0° to m-20°	517
	**7**	t70° to t0°	455	p-10° to m-20°	480	m-20° to t0°	459	t70° to t0°	504
	**8**	m-20° to t70°	428	off to m-20°	462	t0° to m-20°	451	t0° to t70°	489
	**9**	m-20° to t0°	392	t0° to t70°	436	t70° to t0°	413	off to m-20°	458
	**10**	m-20° to p30°	317	m-20° to t70°	399	p30° to m-20°	245	m-20° to t70°	390
HIS	**1**	m80° to m-70°	1306	m80° to m-70°	1148	m80° to m-70°	918	m80° to m-70°	876
	**2**	m170° to m-70°	554	m170° to m-70°	454	m-70° to m80°	527	m-70° to m80°	520
	**3**	p80° to p-80°	336	m-70° to m80°	324	m170° to m-70°	436	m170° to m-70°	376
	**4**	t-160° to t-80°	262	t-160° to t-80°	252	m-70° to t-80°	219	t-80° to t-160°	330
	**5**	m-70° to m80°	239	p-80° to p80°	229	t-160° to t-80°	218	t-80° to m-70°	227
	**6**	t-80° to m-70°	217	p80° to p-80°	222	t-80° to m-70°	211	m-70° to m170°	225
	**7**	off to m-70°	208	m-70° to t-80°	219	p80° to p-80°	184	m-70° to t-80°	217
	**8**	m-70° to t-80°	191	t-80° to t-160°	219	p-80° to p80°	180	p-80° to p80°	198
	**9**	t-80° to t-160°	134	m-70° to m170°	209	off to m-70°	176	t-160° to t-80°	190
	**10**	m-70° to m170°	111	t-80° to m-70°	205	t-80° to t-160°	165	p80° to p-80°	187
ILE	**1**	mm to mt	1916	mm to mt	1734	mm to mt	1904	mm to mt	1786
	**2**	mt to mm	307	mt to mm	643	mt to mm	388	mt to mm	636
	**3**	off to mt	209	tt to mt	199	tt to mt	213	off to mt	200
	**4**	tt to mt	185	off to mt	195	off to mt	206	tt to mt	190
	**5**	mp to mt	174	mp to mt	174	mp to mt	200	mp to mt	166
	**6**	tp to tt	155	mt to tt	171	pt to mt	123	pt to mt	132
	**7**	pp to pt	129	pt to mt	138	tp to tt	122	mt to tt	119
	**8**	tt to pt	127	tp to mt	136	tp to mt	116	tp to tt	118
	**9**	off to pt	120	mt to mp	117	pp to pt	105	tp to mt	116
	**10**	mt to pt	116	tp to tt	110	off to pt	87	pp to pt	96
LEU	**1**	off to mt	1297	tp to mt	1234	tp to mt	1334	tp to mt	1398
	**2**	tp to mt	1071	off to mt	1144	off to mt	1202	off to mt	1159
	**3**	mp to mt	909	mt to tp	969	mt to tp	878	mt to tp	1089
	**4**	mt to tp	702	off to tp	658	mp to mt	753	off to tp	670
	**5**	off to tp	702	mp to mt	640	off to tp	672	mp to mt	653
	**6**	tt to tp	601	mt to mp	608	tt to tp	461	mt to mp	513
	**7**	mt to mp	90	tt to tp	379	mt to mp	198	tt to tp	371
	**8**	off to mp	82	tp to tt	317	off to mp	153	tp to tt	321
	**9**	pp to mt	82	off to mp	194	tp to tt	121	off to mp	175
	**10**	tt to mt	70	tt to mt	85	tt to mt	89	tt to mt	96
PHE	**1**	m-30° to m-85°	710	m-30° to m-85°	758	m-30° to m-85°	563	m-85° to m-30°	782
	**2**	off to t80°	299	m-85° to m-30°	384	m-85° to m-30°	377	m-30° to m-85°	457
	**3**	m-85° to m-30°	227	off to t80°	322	off to t80°	297	m-85° to t80°	332
	**4**	m-85° to t80°	186	m-85° to t80°	247	m-85° to t80°	261	off to t80°	275
	**5**	t80° to m-85°	137	t80° to m-85°	237	t80° to m-85°	250	t80° to m-85°	264
	**6**	off to m-85°	84	p90° to m-85°	140	p90° to m-85°	201	p90° to m-85°	202
	**7**	p90° to m-85°	63	m-30° to t80°	104	m-30° to t80°	94	m-85° to p90°	120
	**8**	m-30° to t80°	58	off to m-85°	67	off to m-85°	85	m-30° to t80°	113
	**9**	m-85° to p90°	51	m-85° to p90°	62	p90° to t80°	73	t80° to m-30°	112
	**10**	off to p90°	51	t80° to m-30°	59	m-85° to p90°	68	p90° to t80°	90
PRO	**1**	Cγ endo to Cγ exo	1988	Cγ endo to Cγ exo	1737	Cγ endo to Cγ exo	1889	Cγ endo to Cγ exo	2097
	**2**	Cγ exo to Cγ endo	1508	off to Cγ endo	1338	Cγ exo to Cγ endo	1356	Cγ exo to Cγ endo	1560
	**3**	off to Cγ endo	1361	off to Cγ exo	1308	off to Cγ endo	1346	off to Cγ endo	1347
	**4**	off to Cγ exo	1285	Cγ exo to Cγ endo	1193	off to Cγ exo	1300	off to Cγ exo	1299
	**5**	Cγ endo to off	3						
TRP	**1**	off to m95°	116	m95° to off	132	off to m95°	144	m95° to off	135
	**2**	t-105° to m95°	73	off to m95°	112	m-90° to m95°	128	m-90° to m95°	112
	**3**	m-90° to m95°	63	m95° to m0°	94	t-105° to m95°	122	off to m95°	112
	**4**	off to m0°	63	off to m0°	93	m0° to m95°	110	p-90° to m95°	108
	**5**	m0° to m95°	59	m95° to t-105°	81	p-90° to m95°	103	t-105° to m95°	107
	**6**	m0° to off	59	t-105° to m0°	76	off to m0°	95	m95° to m-90°	105
	**7**	m95° to t-105°	59	m-90° to m95°	75	p90° to p-90°	70	off to m0°	100
	**8**	off to t-105°	55	m95° to m-90°	75	m95° to t-105°	61	m95° to m0°	87
	**9**	m95° to m-90°	53	t-105° to m95°	72	off to t-105°	52	m95° to t-105°	87
	**10**	p90° to p-90°	47	p90° to p-90°	59	m95° to off	51	m0° to m95°	74
TYR	**1**	m-30° to m-85°	622	m-85° to m-30°	503	m-30° to m-85°	466	m-85° to m-30°	571
	**2**	off to t80°	270	m-30° to m-85°	488	m-85° to m-30°	353	m-30° to m-85°	422
	**3**	m-85° to m-30°	187	m-85° to t80°	273	off to t80°	262	m-85° to t80°	317
	**4**	m-85° to t80°	169	off to t80°	255	m-85° to t80°	243	off to t80°	255
	**5**	t80° to m-85°	146	t80° to m-85°	191	p90° to m-85°	186	t80° to m-85°	244
	**6**	off to m-85°	96	t80° to m-30°	177	t80° to m-85°	182	p90° to m-85°	191
	**7**	m-85° to p90°	83	p90° to m-85°	132	t80° to m-30°	100	t80° to m-30°	173
	**8**	p90° to m-85°	73	m-85° to p90°	111	m-85° to p90°	99	m-85° to p90°	167
	**9**	m-30° to t80°	65	m-30° to t80°	91	off to m-85°	85	m-30° to t80°	87
	**10**	off to p90°	61	off to m-85°	82	m-30° to t80°	79	t80° to p90°	82

a Errors described as “original to predicted”.

**Table 4:** Top 10 Rotamer Errors Found in Group III Residues^a^

residue	rank	FASPR	count	RASP	count	SCWRL4	count	SCWRL4v	count
GLN	**1**	off to mt-30°	1351	off to mt-30°	1162	off to mt-30°	1180	off to mt-30°	1163
	**2**	mt-30° to mm-40°	683	mt-30° to mm-40°	723	mt-30° to mm-40°	741	mt-30° to mm-40°	721
	**3**	off to tt0°	593	off to tt0°	542	off to tt0°	532	off to tt0°	563
	**4**	off to mm-40°	564	off to mm-40°	517	off to mm-40°	519	off to mm-40°	495
	**5**	mm100° to mm-40°	514	mm100° to mm-40°	482	mm100° to mm-40°	465	mm100° to mm-40°	436
	**6**	tt0v to mt-30°	433	off to tp60°	373	tt0° to mt-30°	397	tt0° to mt-30°	412
	**7**	off to tp60°	378	tt0° to mt-30°	348	off to tp60°	391	mm-40° to mt-30°	381
	**8**	mm-40° to mt-30°	325	tt0° to tp60°	337	mm-40° to mt-30°	369	off to tp60°	356
	**9**	tt0° to tp60°	306	mm-40° to mt-30°	322	tt0° to tp60°	352	tt0° to tp60°	280
	**10**	mt-30° to tp60°	236	mt-30° to tp60°	300	mt-30° to tp60°	258	mt-30° to tp60°	246
GLU	**1**	mm-40° to mt-10°	1334	mm-40° to mt-10°	1246	tt0° to mt-10°	1332	tt0° to mt-10°	1373
	**2**	tt0° to mt-10°	1220	tt0° to mt-10°	1214	mm-40° to mt-10°	1148	mm-40° to mt-10°	1312
	**3**	mt-10° to tt0°	791	mt-10° to mm-40°	863	mt-10° to tt0°	898	mt-10° to tt0°	1004
	**4**	mp0° to mt-10°	543	mt-10° to tt0°	755	mp0° to mt-10°	585	mt-10° to mm-40°	660
	**5**	off to mt-10°	510	tt0° to tp10°	446	off to mt-10°	543	mp0° to mt-10°	613
	**6**	mt-10° to mm-40°	485	tp10° to mt-10°	440	mt-10° to mm-40°	503	off to mt-10°	549
	**7**	tp10° to mt-10°	413	off to mt-10°	423	tp10° to mt-10°	368	tp10° to mt-10°	460
	**8**	tp10° to tt0°	401	mp0° to mt-10°	358	pt-20° to mt-10°	367	pt-20° to mt-10°	368
	**9**	off to tt0°	344	tp10° to tt0°	305	off to tt0°	324	mm-40° to tt0°	360
	**10**	mm-40° to tt0°	311	off to tt0°	297	mm-40° to tt0°	311	tp10° to tt0°	356
MET	**1**	mtp to mtm	148	mtp to mtm	164	mtp to mmm	141	mtp to mmm	132
	**2**	off to mtp	106	mtm to mtp	124	mtm to mtp	133	mtm to mtp	126
	**3**	mtt to mtm	94	off to mtm	100	off to mmm	125	mtp to mtm	117
	**4**	off to mtm	93	off to mmm	95	off to mtp	116	off to mmm	116
	**5**	off to mmm	92	mtt to mtm	91	mtp to mtm	108	off to mtp	110
	**6**	mtm to mtp	89	off to mtp	89	mmm to mtp	105	mmm to mtp	95
	**7**	mmm to mtp	79	mmm to mtp	77	mtt to mtm	90	mtt to mtm	91
	**8**	mtt to mtp	78	mtp to mmm	77	off to mtm	88	off to mtm	85
	**9**	mtp to mtt	67	mtt to mtp	71	mtt to mtp	84	mtt to mtp	79
	**10**	off to ttp	66	mtp to mtt	69	mmp to mmm	74	mmm to mmp	70

a Errors described as “original to predicted”.

**Table 5:** Top 10 Rotamer Errors Found in Group IV Residues^a^

residue	rank	FASPR	count	RASP	count	SCWRL4	count	SCWRL4v	count
LYS	**1**	off to mttt	1314	off to mttt	1072	off to mttt	1275	off to mttt	1249
	**2**	mttm to mttt	751	mttm to mttt	628	mttm to mttt	716	mttm to mttt	674
	**3**	off to tttt	582	off to tttt	585	off to tttt	610	off to tttt	617
	**4**	mttp to mttt	574	mttp to mttt	495	mttp to mttt	549	mttp to mttt	520
	**5**	tttt to mttt	523	tttt to mttt	455	mmtt to mttt	494	tttt to mttt	487
	**6**	mmtt to mttt	522	tttm to tttt	440	tttt to mttt	488	mmtt to mttt	481
	**7**	tttm to tttt	407	tttp to tttt	421	tttm to tttt	373	tttm to tttt	379
	**8**	mtmt to mttt	395	mmtt to mttt	358	mtmt to mttt	367	mttt to tttt	373
	**9**	tttp to tttt	391	mtmt to mttt	340	tttp to tttt	365	mtmt to mttt	362
	**10**	mtpt to mttt	384	off to mmtt	336	mtpt to mttt	361	tttp to tttt	355
ARG	**1**	off to mtt180°	662	off to mtt180°	648	off to mtt180°	943	off to mtt180°	983
	**2**	off to mtm-85°	546	off to mtm-85°	578	off to mtm-85°	420	off to mtm-85°	419
	**3**	off to mtm180°	377	off to mtm180°	352	off to mtm180°	350	off to mtt-85°	343
	**4**	off to mtt-85°	306	off to mtt85°	259	off to mtt-85°	324	off to mtm180°	330
	**5**	off to mtt85°	306	off to mtt-85°	250	off to mtp180°	228	mtm-85° to mtt180°	240
	**6**	off to mtp180°	280	off to mtp180°	229	mtm-85° to mtt180°	220	off to mtp180°	236
	**7**	mtt180° to mtm180°	183	mtt-85° to mtt180°	191	off to mmt180°	193	mtp180° to mtt180°	203
	**8**	mtt180° to mtt-85°	173	mtp180° to mtt180°	172	off to mtt85°	187	mtt85° to mtt180°	200
	**9**	mtt-85° to mtt180°	166	mtt180° to off	159	mtt85° to mtt180°	185	off to mmt180°	200
	**10**	off to mtp85°	149	mtt180° to mtm180°	158	mtp180° to mtt180°	182	off to mtt85°	196

a Errors described as “original to predicted”.

In the case of group I residues (CYS, SER, THR, and VAL), these were typically associated with high rates of rotamer prediction accuracy (Table 2 and Figure 4). In the case of VAL, the top two rotamer errors (m to t, and p to t) indicated that the four programs all tend to settle on the t position even when it is not the appropriate choice. As for THR, the programs tended to flip between a preference for m and p rotamers, with a lesser preference for the t rotamer. This is in contrast to CYS where the m rotamer was most preferred by the programs, followed by t rotamer.

Regarding the group II residues (ASN, ASP, HIS, ILE, LEU, PHE, PRO, TRP, and TYR), these were typically associated with high to medium rates of rotamer prediction accuracy (Table 3 and Figure 4). In the case of ASN, the top four rotamer errors were observed to involve a switch to the m-20° rotamer. Following this, the most frequent ASP rotamer error was found to be t70° to m-20° while those for HIS were represented by a tendency toward the m-70° rotamer. In the case of ILE, the most frequent rotamer errors came from an apparent preference for mt followed by mm rotamers. On the other hand, the most frequent rotamer errors for LEU highlight program preferences toward mt followed by tp rotamers. Both PHE and TYR exhibited similar rotamer errors involving m-30° and m-85° rotamers. PRO errors were quite negligible considering the high similarity between the Cγ endo and Cγ exo rotamers and the relatively low frequency of this amino acid residue in the protein data set. Most of the rotamer errors with TRP (where the most frequent error involved off and m95°) seemed to relate to program preferences for the m95° rotamer.

As far as group III residues (GLN, GLU, and MET) are concerned, these were typically associated with accuracy rates of ∼49–63% only (Table 4 and Figure 4). In the case of GLN, the most frequent rotamer error was off to mt-30°, whereas in the case of GLU, the four programs showed tendency to replace rotamers with mt-10°. Aside from a handful of canonical rotamers, the four programs frequently seem to replace off rotamer states of MET with canonical rotamer states.

Finally, in the case of group IV residues (LYS and ARG), these were typically associated with very low rates of rotamer prediction accuracy (Table 5 and Figure 4), and once again the four programs also appeared frequently to replace LYS and ARG off with canonical rotamer states (particularly mttt and tttt in the former, while the latter exhibited a tendency to replace off with a wider range of canonical state replacements).

### Nomenclature Errors

**Figure 6:**
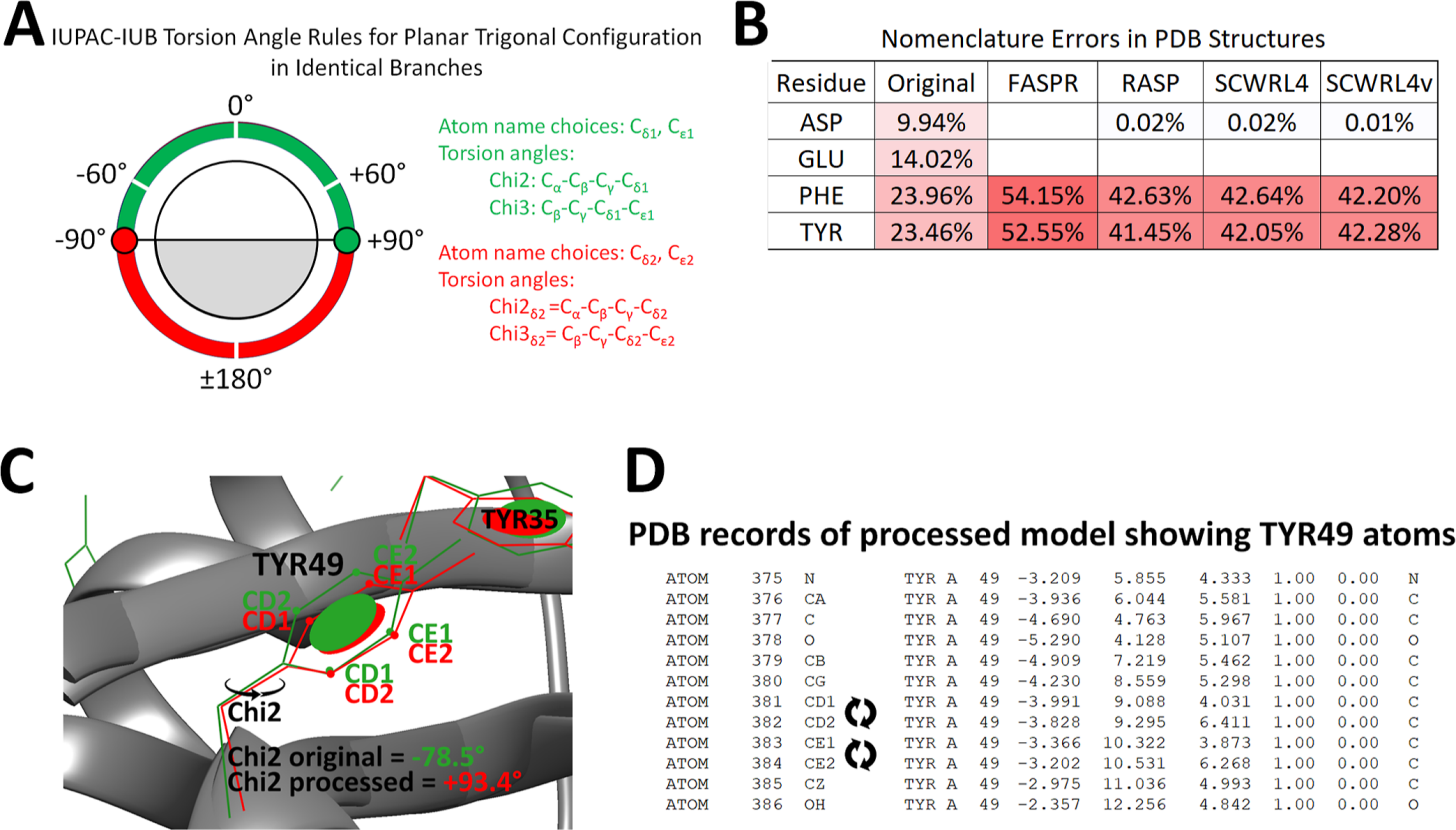
Validity of nomenclature in PDB records according to IUPAC-IUB commission rules. (A) IUPAC-IUB torsion angle rules for the planar trigonal configuration in identical branches, e.g., in Chi3 of TYR. (B) Percentage of records with invalid IUPAC-IUB nomenclature in original and processed structures of MUFOLD-DB (30%) data set using FASPR, RASP, SCWRL4, and SCWRL4v algorithms. (C) Example of invalid TYR nomenclature after processing (visualized by UCSF Chimera program). TYR49 side-chain from scorpion toxin II protein (3D structure PDB ID 1AHO_A) is shown in green (original) and red (after processing with the FASPR program). The choice for TYR49 ring carbon atoms (CD1 and CE1) in processed structure is invalid since the Chi2 angle was in the red range, as shown in the rules in panel (A). (D) PDB records of TYR49 in processed 1AHO_A model showing a quick fix for nomenclature simply by flipping the CD1 and CD2 terms and also CE1 and CE2 terms in the text.

Furthermore, we note here one additional source of error in program execution connected with branched amino acids ASP, GLU, PHE, and TYR. Due to periodicity in branched atoms of these residues, the nonconformity in atom names was resolved by the IUPAC-IUB commission nomenclature rules for planar trigonal configurations for identical branches.^22^ Briefly, in the cases of PHE and TYR, the choice for naming Cδ1 and Cε1 atom pairs versus Cδ2 and Cε2 atom pairs, respectively, is given priority to the lowest torsional angle (Figure 6A). Overall, nomenclature errors were found even in the original PDB files of the MUFOLD data set (9.94, 14.02, 23.96, and 23.46% errors in the cases of ASP, GLU, PHE, and TYR, respectively), as shown in Figure 6B. Interestingly, the four programs were able to manage the nomenclature in ASP and GLU but struggled with PHE and TYR in nearly 41–54% chances. Examples of invalid TYR nomenclature and a quick fix of PDB records are shown in Figure 6C,D. Briefly, the rotamers of TYR49 from scorpion toxin II protein (PDB ID 1AHO_A) were analyzed by superposition using original pdb data and FASPR-processed models. Despite the nearly superposed TYR aromatic rings, the choice for naming Cδ1 and Cε1 atoms (CD1 and CD2 atoms in PDB records, respectively), in the processed model was invalid according to IUPAC-IUB rules of torsion angles (Figure 6A). A quick fix for nomenclature in the PDB records can be made simply by flipping the CD1 and CD2 names and also CE1 and CE2 terms.

## Rotamers and Solvent Accessibility

With reference to the studied data set (Table 1), this comprised 422,379 rotamers divided as follows: 393,556 canonical and 28,823 off rotamers. After preparation, this data set was used to study the implied correlation between the appearance of off rotamers and high solvent accessibility, considering the situation with group I residues (CYS, SER, THR, and VAL) was typically associated with high rates of rotamer prediction accuracy (Figure 4). Off rotamer conformational states were also rare ≤1% and generally associated with high or the highest ACC values (14.57 ± 20.45, 47.35 ± 36.61, 50.60 ± 41.70 and 40.26 ± 42.90 Å^2^, respectively), as compared to the situation with canonical rotamer conformational states (Table 6). Hence, confidence in prediction of off rotamers was zero as far as group I residues were concerned. The situation with group II residues (ASN, ASP, HIS, ILE, LEU, PHE, PRO, TRP, and TYR) was also associated with high rates of accurate rotamer prediction (Figure 4). Once again, off rotamer conformational states were rare but are not always correlated with high or the highest ACC values (Table 7). For example, off rotamer conformational states of ILE and LEU were found associated with the highest ACC scores (31.82 ± 39.31 and 25.46 ± 36.39 Å^2^, respectively), while off rotamer conformational states of TRP were associated with the lowest of ACC score (31.24 ± 43.32 Å^2^). Again, confidence in prediction of off rotamers was zero as far as the group II residues were concerned. In the case of group III, residues (GLN, GLU, and MET) were typically associated with lower rates of rotamer prediction accuracy (Figure 4) while off rotamer conformational states were found associated with high but not necessarily the highest ACC values (Table 8). Finally, with reference to group IV residues (ARG and LYS) were certainly associated with low rates of rotamer prediction accuracy (Figure 4), and off rotamer conformational states were found associated with middle to the highest ACC values (80.45 ± 52.87 and 96.55 ± 48.06 Å^2^, respectively), as compared to the situation with canonical rotamer conformational states (Table 9). It is worth noting that off rotamers of ARG were the most frequent, hence confidence in prediction of off rotamers was high.

**Table 6:** Solvent Accessibility across Rotamers of Group I Residues (ACC Score in Å^2^ Units)

residue	rotamer	frequency	percent	solvent accessibility	confidence
				(ACC mean ± SD)	(%)
CYS	m	3674	55.6	13.03 ± 19.65	90.3
	p	1102	16.7	15.51 ± 20.68	69.4
	t	1786	27.0	12.00 ± 16.98	73.7
	off	44	0.7	14.57 ± 20.45	0.0
SER	m	8143	27.8	34.42 ± 34.47	39.6
	p	14,059	48.1	42.24 ± 34.10	79.7
	t	6770	23.1	28.26 ± 29.61	59.1
	off	283	1.0	47.35 ± 36.61	0.0
THR	m	12,932	43.8	38.27 ± 34.82	91.8
	p	14,256	48.3	45.33 ± 37.23	91.1
	t	2146	7.3	25.52 ± 30.73	63.0
	off	170	0.6	50.60 ± 41.70	0.0
VAL	m	7475	18.7	23.49 ± 30.31	83.9
	p	2664	6.7	19.67 ± 28.70	54.1
	t	29,652	74.2	19.74 ± 28.57	97.3
	off	166	0.4	40.26 ± 42.90	0.0

**Table 7:** Solvent Accessibility across Rotamers of Group II Residues (ACC Score in Å^2^ units)

residue	rotamer	frequency	percent	solvent accessibility	confidence
				(ACC mean ± SD)	(%)
ASN	m-20°	7003	32.6	65.06 ± 44.06	78.2
	m-80°	2021	9.4	50.96 ± 41.66	16.0
	m120°	2120	9.9	66.87 ± 47.22	0.4
	p-10°	1194	5.6	53.13 ± 41.40	29.1
	p30°	1548	7.2	50.26 ± 35.94	65.8
	t-20°	2704	12.6	43.82 ± 34.43	60.4
	t30°	2808	13.1	58.80 ± 44.91	34.1
	off	2062	9.6	52.99 ± 45.36	0.0
ASP	m-20°	14,654	49.1	68.79 ± 42.88	85.6
	p-10°	2537	8.5	66.21 ± 41.55	11.5
	p30°	2521	8.4	58.03 ± 37.71	58.0
	t0°	6885	23.0	51.42 ± 37.74	74.9
	t70°	2558	8.6	50.96 ± 37.48	34.6
	off	718	2.4	53.96 ± 47.89	0.4
HIS	m-70°	3647	28.9	58.06 ± 47.34	59.6
	m170°	1097	8.7	55.49 ± 46.64	22.4
	m80°	1710	13.6	55.44 ± 44.67	6.2
	p-80°	857	6.8	50.41 ± 45.62	43.4
	p80°	609	4.8	45.37 ± 46.40	22.5
	t-160°	528	4.2	39.21 ± 38.73	27.8
	t-80°	3611	28.6	51.97 ± 43.92	75.5
	off	553	4.4	46.16 ± 47.26	0.4
ILE	mm	4979	15.9	21.48 ± 30.29	44.3
	mp	335	1.1	15.90 ± 28.17	12.2
	mt	18,837	60.2	18.70 ± 29.03	92.3
	pp	167	0.5	29.62 ± 39.65	8.4
	pt	3897	12.4	26.02 ± 33.77	89.2
	tp	831	2.7	25.31 ± 33.52	41.4
	tt	1811	5.8	19.65 ± 31.20	66.8
	off	452	1.4	31.82 ± 39.31	0.0
LEU	mp	1278	2.5	18.73 ± 30.12	19.1
	mt	31,694	61.2	25.90 ± 34.22	92.3
	pp	374	0.7	16.55 ± 28.84	56.4
	tp	15,053	29.1	20.19 ± 30.34	83.9
	tt	1063	2.1	15.61 ± 28.37	27.4
	off	2334	4.5	25.46 ± 36.39	5.5
PHE	m-30°	2047	8.8	24.88 ± 36.96	45.2
	m-85°	10,655	45.7	27.92 ± 38.47	88.6
	p90°	2542	10.9	24.46 ± 34.47	87.2
	t80°	7563	32.4	27.21 ± 37.27	92.5
	off	521	2.2	23.11 ± 40.36	5.6
PRO	Cγ Endo	11,230	43.5	45.64 ± 37.23	76.0
	Cγ Exo	11,929	46.2	53.58 ± 37.44	82.7
	off	2646	10.3	50.47 ± 37.67	0.0
TRP	m-90°	409	4.9	37.72 ± 45.69	50.4
	m0°	791	9.5	35.75 ± 41.98	62.5
	m95°	2654	31.7	39.29 ± 44.12	78.8
	p-90°	812	9.7	48.58 ± 51.71	72.7
	p90°	390	4.7	46.15 ± 49.88	55.1
	t-105°	2840	34.0	37.17 ± 42.68	89.3
	off	469	5.6	31.24 ± 43.32	13.2
TYR	m-30°	1577	7.6	40.40 ± 46.62	42.0
	m-85°	9384	45.4	43.71 ± 44.51	86.0
	p90°	2340	11.3	41.34 ± 41.67	85.6
	t80°	6854	33.2	44.94 ± 44.88	89.7
	off	497	2.4	29.68 ± 37.84	6.4

**Table 8:** Solvent Accessibility across Rotamers of Group III Residues (ACC Score in Å^2^ Units)

residue	rotamer	frequency	percent	solvent accessibility	confidence
				(ACC mean ± SD)	(%)
GLN	mm-40°	1986	12.3	71.41 ± 46.65	53.6
	mm100°	799	4.9	73.20 ± 47.93	0.4
	mp0°	428	2.6	61.42 ± 46.29	13.6
	mt-30°	4975	30.7	70.07 ± 46.42	57.2
	pm0°	170	1.1	61.51 ± 42.17	34.7
	pt20°	606	3.7	66.34 ± 49.33	38.8
	tp-100°	372	2.3	57.63 ± 42.82	0.3
	tp60°	1205	7.4	66.32 ± 42.85	55.4
	tt0°	2385	14.7	67.21 ± 46.86	45.5
	off	3264	20.2	68.20 ± 48.56	1.7
GLU	mm-40°	3843	14.6	77.96 ± 45.76	32.6
	mp0°	1572	6.0	78.12 ± 45.89	22.9
	mt-10°	9296	35.2	80.03 ± 46.05	62.1
	pm0°	597	2.3	78.61 ± 49.27	38.4
	pt-20°	1353	5.1	77.20 ± 47.43	42.4
	tm-20°	319	1.2	67.78 ± 46.71	5.3
	tp10°	1850	7.0	69.41 ± 43.71	24.9
	tt0°	6088	23.1	76.83 ± 46.82	50.6
	off	1472	5.6	75.29 ± 53.43	0.3
MET	mmm	1302	18.0	29.86 ± 37.56	59.1
	mmp	282	3.9	28.13 ± 38.01	38.7
	mmt	232	3.2	27.31 ± 38.18	23.7
	mtm	774	10.7	25.32 ± 38.63	54.8
	mtp	1179	16.3	26.96 ± 36.96	49.2
	mtt	566	7.8	24.49 ± 37.58	40.3
	ptm	176	2.4	24.09 ± 33.91	49.4
	ptp	160	2.2	32.14 ± 45.02	40.0
	tpp	427	5.9	23.25 ± 34.78	48.9
	tpt	84	1.2	17.54 ± 28.59	33.3
	ttm	433	6.0	29.79 ± 41.93	39.0
	ttp	504	7.0	24.64 ± 37.83	53.6
	ttt	216	3.0	20.37 ± 32.52	31.5
	off	905	12.5	29.61 ± 41.75	15.4

**Table 9:** Solvent Accessibility across Rotamers of Group IV Residues (ACC Score in Å^2^ Units)

residue	rotamer	frequency	percent	solvent accessibility	confidence
				(ACC mean ± SD)	(%)
ARG	mmm-85°	463	2.2	76.38 ± 50.89	13.8
	mmm180°	313	1.5	92.50 ± 56.45	8.3
	mmt-85°	567	2.7	69.40 ± 48.17	42.3
	mmt180°	500	2.4	81.74 ± 53.07	15.8
	mmt85°	244	1.2	84.86 ± 59.50	7.8
	mtm-85°	1207	5.7	96.19 ± 52.25	27.6
	mtm105°	316	1.5	79.18 ± 51.89	6.0
	mtm180°	1005	4.7	83.09 ± 55.50	27.3
	mtp-105°	254	1.2	90.11 ± 57.74	0.4
	mtp180°	958	4.5	85.27 ± 52.79	16.3
	mtp85°	775	3.7	84.24 ± 54.85	14.7
	mtt-85°	1150	5.4	84.91 ± 53.49	21.5
	mtt180°	1731	8.2	87.95 ± 55.41	28.2
	mtt85°	922	4.4	84.11 ± 50.55	15.3
	ptm-85°	72	0.3	94.32 ± 63.31	5.6
	ptm180°	146	0.7	79.80 ± 53.66	11.0
	ptp180°	184	0.9	74.55 ± 57.14	17.9
	ptp85°	100	0.5	87.75 ± 50.42	6.0
	ptt-85°	282	1.3	82.45 ± 52.36	21.3
	ptt180°	303	1.4	74.49 ± 51.93	28.1
	ptt85°	337	1.6	82.97 ± 50.41	30.3
	off	9347	44.1	80.45 ± 52.87	50.0
LYS	mmmt	309	1.5	81.42 ± 45.12	5.5
	mmtm	433	2.1	86.64 ± 45.69	2.3
	mmtp	289	1.4	94.41 ± 45.70	0.3
	mmtt	1532	7.3	85.84 ± 47.19	30.4
	mptt	65	0.3	86.22 ± 52.75	0.0
	mtmm	250	1.2	81.30 ± 53.49	3.6
	mtmt	724	3.5	85.57 ± 48.77	9.9
	mtpp	239	1.1	87.10 ± 48.07	0.8
	mtpt	736	3.5	86.83 ± 44.67	5.3
	mttm	1058	5.1	94.08 ± 46.85	2.5
	mttp	800	3.8	97.66 ± 48.74	2.3
	mttt	4358	20.9	95.38 ± 45.80	59.1
	ptmt	104	0.5	92.13 ± 45.07	1.9
	ptpt	95	0.5	84.62 ± 47.57	7.4
	pttm	145	0.7	83.90 ± 48.33	2.1
	pttp	149	0.7	86.17 ± 51.57	2.0
	pttt	694	3.3	93.24 ± 47.78	42.2
	tptm	124	0.6	82.31 ± 48.43	0.0
	tptp	190	0.9	76.33 ± 47.29	3.2
	tptt	544	2.6	82.88 ± 45.52	16.2
	ttmm	123	0.6	86.38 ± 45.07	0.0
	ttmt	385	1.8	81.26 ± 43.97	6.0
	ttpp	148	0.7	75.61 ± 46.29	2.0
	ttpt	475	2.3	84.62 ± 45.11	11.4
	tttm	719	3.4	94.68 ± 45.24	1.5
	tttp	686	3.3	98.43 ± 43.81	3.1
	tttt	2567	12.3	94.83 ± 46.56	45.1
	off	2920	14.0	96.55 ± 48.06	0.7

It is important to emphasize on the fact that rotamer classes were often associated with distinct mean ACC despite the standard deviation in the range of 30–50 Å^2^ ACC. A statistical analysis was also used to investigate and further detail the general correlation between off rotamer conformational states and high or highest ACC values (Tables 10, S1 and S2). Overall, a significant positive correlation was found between high ACC values and errors involving off rotamers while a negative correlation was found mostly with ARG, ASN, ASP, HIS, TRP, and TYR residues (Table 10). With regard to top canonical-to-canonical errors, significant differences were found with mean ACC values between frequent canonical-to-canonical error combinations (Tables S1 and S2). The following is a summary of significant canonical-to-canonical error combinations followed by the equivalent mean ACC difference: SER (p-m 7.82 Å^2^), THR (p-m 7.06 Å^2^), VAL (t-m −3.75 Å^2^), ILE (mt-mm −2.78 Å^2^), LEU (tp-mt −5.72 Å^2^), and PRO (Cγ exo-Cγ endo 7.94 Å^2^). Exceptions where top canonical-to-canonical errors did not show a significant difference with mean ACC values include CYS (p-m 2.47; t-m −1.04 Å^2^), ASN (m120°–m-20° 1.81 Å^2^), ASP (p30°–p-10° −2.88 Å^2^), HIS (m80°–m-70° −2.63 Å^2^), PHE (m-85°–m-30° 3.05 Å^2^), TYR (m-85°–m-30° 3.32 Å^2^), GLU (mt-10°–mm-40° 2.07 Å^2^), and MET (mtp–mtm 1.64 Å^2^) (Tables S1 and S2).

**Table 10:** Significant Mean Difference in Solvent Accessibility between off Rotamers and Canonical Rotamers^a^

residue	variable	difference (Å^2^)	lower CI	upper CI	*p* original	*p* adjusted	correlation (off rotamers vs high ACC)
ARG	off-mtm-85°	–15.74	–21.59	–9.89	0.00	0.00	
ARG	off-mtt180°	–7.49	–12.50	–2.49	1.67 × 10^–^^5^	1.61 × 10^–^^2^	
ASN	off-m-20°	–12.07	–15.31	–8.83	0.00	0.00	
ASN	off-m120°	–13.88	–17.87	–9.88	0.00	0.00	
ASN	t-20°-off	–9.18	–12.95	–5.40	0.00	0.00	+
ASP	off-m-20°	–14.83	–19.28	–10.37	5.15 × 10^–^^14^	4.96 × 10^–^^11^	
ASP	p-10°-off	12.25	7.32	17.18	2.08 × 10^–^^11^	2.00 × 10^–^^8^	
HIS	off-m-70°	–11.90	–18.19	–5.61	2.74 × 10^–^^7^	2.64 × 10^–^^4^	
ILE	off-mm	10.34	5.82	14.86	1.10 × 10^–^^1^	1.06 × 10^–^^7^	+
ILE	off-mp	15.92	9.29	22.54	9.54 × 10^–^^12^	9.19 × 10^–^^9^	+
ILE	off-mt	13.12	8.75	17.50	1.02 × 10^–^^13^	9.81 × 10^–^^11^	+
ILE	tt-off	–12.17	–17.00	–7.33	7.15 × 10^–^^13^	6.88 × 10^–^^1^	+
LEU	off-mp	6.73	3.45	10.00	6.98 × 10^–^^8^	6.72 × 10^–^^5^	+
LEU	pp-off	–8.91	–14.15	–3.68	1.83 × 10^–^^5^	1.76 × 10^–^^2^	+
LEU	tp-off	–5.27	–7.36	–3.18	1.05 × 10^–^^11^	1.01 × 10^–^^8^	+
LEU	tt-off	–9.84	–13.32	–6.37	8.78 × 10^–^^14^	8.46 × 10^–^^11^	+
LYS	off-mmmt	15.12	4.74	25.50	2.30 × 10^–^^5^	2.22 × 10^–^^2^	+
LYS	off-mmtt	10.70	5.23	16.18	0.00	0.00	+
LYS	off-mtmt	10.97	3.77	18.18	5.69 × 10^–^^6^	5.48 × 10^–^^3^	+
LYS	tptp-off	–20.22	–33.21	–7.23	2.72 × 10^–^^6^	2.62 × 10^–^^3^	+
LYS	tptt-off	–13.67	–21.77	–5.57	7.23 × 10^–^^8^	6.96 × 10^–^^5^	+
LYS	ttmt-off	–15.28	–24.69	–5.88	5.38 × 10^–^^7^	5.18 × 10^–^^4^	+
LYS	ttpp-off	–20.93	–35.55	–6.31	3.85 × 10^–^^5^	3.70 × 10^–^^2^	+
PRO	off-Endo	4.83	2.94	6.72	6.65 × 10^–^^9^	6.41 × 10^–^^6^	+
SER	off-m	12.93	7.76	18.09	7.69 × 10^–^^1^	7.41 × 10^–^^7^	+
SER	t-off	–19.08	–24.27	–13.90	3.51 × 10^–^^14^	3.38 × 10^–^^11^	+
THR	off-m	12.33	5.24	19.43	4.70 × 10^–^^5^	4.53 × 10^–^^2^	
THR	t-off	–25.08	–32.40	–17.76	3.06 × 10^–^^14^	2.95 × 10^–^^11^	+
TRP	p-90°-off	17.34	9.72	24.96	4.61 × 10^–^^1^	4.44 × 10^–^^7^	
TRP	p90°-off	14.91	5.91	23.92	2.19 × 10^–^^5^	2.11 × 10^–^^2^	
TYR	off-m-30°	–10.72	–16.94	–4.50	2.59 × 10^–^^5^	2.49 × 10^–^^2^	
TYR	off-m-85°	–14.04	–19.60	–8.47	0.00	0.00	
TYR	p90°-off	11.66	5.69	17.64	9.55 × 10^–^^7^	9.20 × 10^–^^4^	
TYR	t80°-off	15.26	9.64	20.88	0.00	0.00	
VAL	off-m	16.77	10.93	22.62	1.02 × 10^–^^12^	9.87 × 10^–^^1^	+
VAL	p-off	–20.59	–26.54	–14.63	2.91 × 10^–^^14^	2.80 × 10^–^^11^	+
VAL	t-off	–20.52	–26.31	–14.72	5.10 × 10^–^^14^	4.91 × 10^–^^11^	+

a Tukey’s “honest significant difference” method was used for ANOVA post hoc.

## Discussion

Protein side-chain packing prediction is an important process in de novo and homology modeling. Rotamers (or rotational isomers) describe clusters of side-chain biophysical shapes recorded in experimental data such as X-ray crystallography or molecular dynamics simulations.^16,19,25,26^ Rotamers are tightly packed in proteins providing structural stability while some are flexible and might play functional roles in interactions and active sites. Nowadays, several advanced side-chain packing prediction programs are used to provide accurate predictions of rotamers and have been utilized in building protein 3D models for various applications such as drug design and enzyme engineering to name a few.

Herein, we have investigated the performance of four side-chain packing prediction programs (FASPR, RASP, and SCWRL4) by processing 3D structure files of the MUFOLD-DB data set, which constitutes low homology contiguous chain collection of proteins. The processed data set contained 393,556 and 28,823 canonical and off rotamers, respectively (Table 1). We have also analyzed the data set to keep perspective on the naturally observed frequencies of rotamers without excluding rotamer outliers. The issue whether the off rotamers are real or artifacts of crystallography method had been a long question in biology. Petrella and Karplus^27^ employed energy-based rotational maps—obtained from molecular dynamics data—to uncover a rough estimate of 63.8% of off rotamers to be real while the remaining were artifacts of the X-ray refinements. Our data set was filtered for B factor quality and full occupancy to reduce artifacts.

Using the discretized rotamer analysis, all four programs demonstrated high rates of rotamer prediction accuracy (74–76%) (Figures 3 and 4) although the SCWRL4v algorithm (using a fixed rotamer option) was the least effective. This was generally expected since the four programs use the same rotamer library (Dunbrack backbone-dependent library) for their rotamer collection. Importantly, the accuracies of all four side-chain prediction programs, based on discretized rotamer classes across amino acid residues, appeared very amino acid residue dependent (Figure 3). Indeed, when the amino acid residue frequency was taken into consideration (Figures 3E–H and 4), then rotamer prediction accuracies segregated into three distinct amino acid residue groupings. This same analysis also demonstrated that rotamer prediction errors were largely secondary structure and protein size independent but clearly dependent on increasing ACC values (Figure 4C).

In terms of the percentage of rotamer prediction errors per amino acid residue, errors were lowest for small and nonpolar amino acid residues and highest for charged or polar amino acid residues LYS, ARG, and GLN compared with other residues (Figure 4A). Using the Chi angle analysis instead of discretized rotamer analysis, errors in “Chi angle prediction” within a ±30° cut-off were either largely constant or increased up to an ACC value of approx 100 Å^2^, then declined thereafter when the percentage was divided by total number of cases (Figure S4). A very steady increase in errors was observed for each solvent accessibility interval in all Chi angles, highlighting the role of solvent accessibility in directly impacting each Chi angle independently (Figure 5). In grouping amino acid residues into four group categories according to their side-chain length and Chi angles, rotamer prediction errors were found to be substantially canonical to canonical with group I (Table 2) and group II residues except TRP (Table 3) but increasingly off rotamer to canonical with group III (Table 4) and group IV residues (Table 5).

Energy-based rotational maps of Chi angles described by Petrella and Karplus^27^ can be interpreted by the following ranking of rotamer classes based on lowest energy dips (dips with less than 1 kcal/mol difference are separated by a comma): ILE Chi1 (p,t,m; most frequent is m), ILE Chi2 (t < m,p; most frequent is t), LEU Chi1 (m,t < p; most frequent are m and t), LEU Chi2 (t < p < m; most frequent are p and t), MET Chi1 (m < p,t; most frequent is m), MET Chi2 (m,t < p; most frequent is t), MET Chi3 (m,t < p), PHE Chi1 (m < t < p; most frequent is m and t), PHE Chi2 (105°), TRP Chi1 (m < p,t; most frequent is m and t), TRP Chi2 (100°,-85°; most frequent is 100°), and VAL Chi1 (m,t < p; most frequent is t). Taking into consideration the combinatorial nature of energy calculations, this summary of energy–rotamer correlations is in line with our findings with regard to the fact that frequent rotamers often display the lowest energy. Indeed, herein, most frequent rotamers of ILE were mt, mm, and pt at 60.2, 15.9, and 12.4%, respectively (Table 7), most frequent rotamers of LEU were mt and tp at 61.2 and 29.1%, respectively (Table 7), most frequent rotamers of MET were mmm, mtp, and off at 18.0, 16.3, and 12.5%, respectively (Table 8), most frequent rotamers of PHE were m-85°, t80°, and p90° at 45.7, 32.4, and 10.9%, respectively (Table 7), the most frequent rotamers of TRP were t-105° and m95° at 34.0 and 31.7%, respectively (Table 7), while the most frequent rotamers of VAL were t and m at 74.2 and 18.7%, respectively (Table 6).

The general correlation between the adoption of off rotamer conformational states at high or highest ACC values (Table 10) suggested to us that off rotamer states may be stabilized in some way by increased solvent accessibility. Importantly, this suggestion is supported by Zhu et al.,^28^ who demonstrated through quantum mechanical energy studies that solvent plays a key role in stabilizing unfavorable conformations in polar or charged side chains while both the protein itself and the solvent have minimal influence on side-chain conformations of hydrophobic amino acids. Hence overall, charged and polar amino acid residues appear to have a propensity to adopt off rotamer classes in native proteins aided in all likelihood by favorable energetics from solvent accessibility. This is in line with our analysis. Unfortunately, our data here show clearly that all four side-chain packing prediction programs appear largely unable to handle properly the presence of off rotamers due to their tendency to mis-assign such classes to alternative canonical rotamers. This is very clear from the confidence scores for off rotamers in comparison to canonical rotamers (Tables 6–9). Accordingly, all this raises the key question, how can such errors be avoided going forward?

The addition of the side-chains is the last step in “model-building” in homology modeling, and it comes prior to model optimization (or refinement).^29^ Several studies highlighted the importance of solvent accessibility in the refinement stage for improvement of homology models by adding the solvent implicitly (as surface potential) or explicitly (as water molecule model) to molecular simulations.^30,31^ The solvent accessibility can be also added to the energy function in refinement.^32^ Furthermore, in one study, the consistency between observed and predicted solvent accessibility was used as a prediction feature for model refinement using deep learning.^33^ Researchers studying X-ray crystal structures and NMR employ similar methods of refinement (in such cases, the experimental data are used as distance restraints for model in molecular simulations). Among the lessons learned from NMR structure refinement is the greater performance of explicit over implicit solvent; “this is due to the missing energetic and entropic contributions and hydrogen-bonding capacities of the water molecules and the missing dielectric screening effect of this high-permittivity solvent”.^34^

Until now, the solvent accessibility issue has been intensely treated post hoc as a refinement problem and not as a side-chain packing prediction problem. However, once the source of the problem is identified and understood, then it should be theoretically possible to address it at earlier stage. Thus, going back to the three pillars of the protein side-chain packing problem, several strategies can be devised to reduce side-chain packing prediction errors:

1. Exploring the application of new statistical clustering of side-chain conformations that is weighted by solvent accessibility, i.e., developing solvent accessibility-dependent rotamer libraries: the choice of rotamer library has direct impact on accuracy. In a recent study by the same FASPR team, Huang et al.^13^ demonstrated clear shift in accuracy when comparing the use of six different rotamer libraries. They also showed improved performance by backbone-dependent rotamer libraries due to addition of backbone energy term in the energy function and due to sectioning of the library to more speed-efficient subsets. On the other hand, rotamer libraries that explore the full dynamic range of conformations in solution such as the Dynameomics library^26^ provide much larger sampling of rotamers particularly at the extreme solvent accessibility spectrum. This library, which is also backbone-dependent, could be better choice for packing the highly accessible pockets and outer shell of the protein (i.e., the residues directly facing solvent). Our proposed strategy is to first calculate the solvent accessibility (directly or from a mock rotamer packing), and then, the rotamers could be selected from either library based on a threshold value for solvent exposure. There is one innovation that is also worth mentioning which is rotamer pair library where the conformations are sampled in consecutive pairs of amino acids, thus further subsetting the backbone-dependent library.^35^
2. Giving more weight to solvent accessibility in combinatorial problem search strategies and scoring functions: adding solvation-related energy terms to scoring functions could be achieved directly. On the other hand, the solvent ACC is a more quantitative attribute for amino acid residues as compared to core vs surface attributes previously described.^11^ Rotamers belonging to long-chain polar amino acid residues with an enhanced tendency to adopt off rotamer states need to be re-evaluated energetically with solvent stabilization in mind, in order to reduce potential errors in rotamer prediction. Rotamers belonging to amino acid residues in the interaction with exogenous ligands may also need to be re-evaluated energetically too just in case these interactions unexpectedly stabilize some rotamers in preference to others.
3. Developing error prediction algorithms based on processed data sets (e.g., like the one described in this work): for example, a sequence-based error prediction tool can be used to determine hotspots. Once a hotspot site for error is predicted, then it can be automatically analyzed for and fixed. Alternatively, an ACC-based error hotspot prediction tool can be developed.
4. Taking specialized route for error correction, i.e., residue by residue, where each amino acid demands a unique scenario: in other words, also focusing on clusters of residues with high error rate by targeting ARG, LYS, and GLN rotamers.
5. Improving discretized rotamer analysis by classifying off rotamers in more depth, or alternatively to explore the range of the canonical rotamers discretization to six or more bins per torsional angle.

It is obvious that solvent accessibility inadvertently promotes rotamer errors in side-chain prediction programs. The fact that off rotamers are associated with both errors and solvent accessibility allows us to hypothesize that higher-energy off rotamers, classically speaking, can be stabilized favorably by solvent interactions in preference to those more conventionally stable rotamers preferred by current rotamer libraries. A residue by residue full scale energetics analysis is required to test this hypothesis, which is beyond the scope of this work. Investigation of both rotamer dynamics and per-residue energy decomposition will be the upcoming steps for this work.

In conclusion, we have demonstrated here, when using four side-chain packing prediction programs, a likely correlation between high frequencies of rotamer errors and high solvent accessibilities with charged and polar amino acid residues (ARG, GLN, GLU, LYS, MET, and ASN). Overall, the results described here provide insights toward improved accuracy in side-chain packing predictions.

## Supporting Information Available

Detailed mean differences in solvent accessibility across rotamer classes; Side-chain prediction accuracy based on discretized rotamer classes; Side-chain prediction errors based on Chi angles deviation by 30° from original (PDF)

## Notes

The data and code underlying this article are deposited in Zenodo (https://doi.org/10.5281/zenodo.7934793) under Creative Commons Attribution 4.0 International licence.
