# Supplementary material for: Solvent Accessibility Promotes Rotamer Errors during Protein Modeling with Major Side-Chain Prediction Programs
Source: J Chem Inf Model. 2023 Jul 6;63(14):4405–22. doi: 10.1021/acs.jcim.3c00134 (PMC10369486; doi:10.1021/acs.jcim.3c00134)
Supplement: Supplementary file 1 — ci3c00134_si_001.pdf [file ci3c00134_si_001.pdf]

Supporting Information

Solvent Accessibility Promotes Rotamer Errors During Protein Modelling with Major Side-Chain Prediction Programs

Tareq Hameduh, Michal Mokry, Andrew D. Miller, Zbynek Heger and Yazan Haddad

Table S1. Analysis of Variance (ANOVA) test for mean difference between rotamers according to solvent accessibility. ($p < 0.05$ is significant).

Residue	f_value	p_value
ARG	9.158	1.67E-29
ASN	104.576	3.52E-151
ASP	225.650	3.15E-237
CYS	7.843	3.19E-05
GLN	6.772	9.19E-10
GLU	12.822	1.38E-18
HIS	19.406	5.28E-26
ILE	44.081	1.86E-62
LEU	85.774	4.24E-90
LYS	10.619	7.18E-45
MET	3.057	1.57E-04
PHE	7.591	4.16E-06
PRO	130.923	2.68E-57
SER	297.539	2.76E-190
THR	232.438	4.59E-149
TRP	11.870	2.63E-13
TYR	17.107	5.14E-14
VAL	59.669	1.76E-38

Table S2. Tukey's 'Honest Significant Difference' test for mean difference between rotamers according to solvent accessibility, used as Analysis of Variance (ANOVA) *post hoc* (*: Adjusted p <0.05 is significant).

No.	Residue	Variable	Difference (Å^2)	Lower CI	Upper CI	p original	p adjusted	
1	ARG	mmm180°-mmm-85°	16.13	2.13	30.12	6.59E-03	6.34E+00	
2	ARG	mmt-85°-mmm-85°	-6.97	-18.95	5.01	8.93E-01	8.60E+02	
3	ARG	mmt180°-mmm-85°	5.36	-6.97	17.69	9.96E-01	9.59E+02	
4	ARG	mmt85°-mmm-85°	8.48	-6.64	23.61	9.23E-01	8.89E+02	
5	ARG	mtm-85°-mmm-85°	19.82	9.36	30.27	0.00E+00	0.00E+00	*
6	ARG	mtm105°-mmm-85°	2.81	-11.15	16.76	1.00E+00	9.63E+02	
7	ARG	mtm180°-mmm-85°	6.71	-4.03	17.46	8.12E-01	7.82E+02	
8	ARG	mtp-105°-mmm-85°	13.74	-1.19	28.67	1.21E-01	1.16E+02	
9	ARG	mtp180°-mmm-85°	8.90	-1.93	19.72	2.91E-01	2.81E+02	
10	ARG	mtp85°-mmm-85°	7.86	-3.37	19.10	6.20E-01	5.97E+02	
11	ARG	mtt-85°-mmm-85°	8.54	-1.99	19.06	3.17E-01	3.05E+02	
12	ARG	mtt180°-mmm-85°	11.57	1.56	21.58	6.24E-03	6.01E+00	
13	ARG	mtt85°-mmm-85°	7.73	-3.16	18.63	5.92E-01	5.70E+02	
14	ARG	off-mmm-85°	4.07	-5.03	13.18	9.94E-01	9.57E+02	
15	ARG	ptm-85°-mmm-85°	17.94	-6.28	42.17	5.04E-01	4.85E+02	
16	ARG	ptm180°-mmm-85°	3.43	-14.73	21.58	1.00E+00	9.63E+02	
17	ARG	ptp180°-mmm-85°	-1.83	-18.49	14.84	1.00E+00	9.63E+02	
18	ARG	ptp85°-mmm-85°	11.37	-9.71	32.46	9.47E-01	9.12E+02	
19	ARG	ptt-85°-mmm-85°	6.07	-8.37	20.52	9.97E-01	9.60E+02	
20	ARG	ptt180°-mmm-85°	-1.89	-16.02	12.24	1.00E+00	9.63E+02	
21	ARG	ptt85°-mmm-85°	6.59	-7.10	20.29	9.84E-01	9.48E+02	
22	ARG	mmt-85°-mmm180°	-23.10	-36.57	-9.64	7.16E-08	6.90E-05	*
23	ARG	mmt180°-mmm180°	-10.77	-24.55	3.01	3.92E-01	3.77E+02	
24	ARG	mmt85°-mmm180°	-7.64	-23.98	8.69	9.89E-01	9.52E+02	
25	ARG	mtm-85°-mmm180°	3.69	-8.44	15.82	1.00E+00	9.63E+02	
26	ARG	mtm105°-mmm180°	-13.32	-28.57	1.93	1.89E-01	1.82E+02	
27	ARG	mtm180°-mmm180°	-9.41	-21.79	2.96	4.48E-01	4.31E+02	
28	ARG	mtp-105°-mmm180°	-2.39	-18.54	13.76	1.00E+00	9.63E+02	
29	ARG	mtp180°-mmm180°	-7.23	-19.68	5.22	8.95E-01	8.62E+02	
30	ARG	mtp85°-mmm180°	-8.26	-21.07	4.54	7.66E-01	7.38E+02	
31	ARG	mtt-85°-mmm180°	-7.59	-19.79	4.60	8.17E-01	7.87E+02	
32	ARG	mtt180-mmm180°	-4.56	-16.31	7.19	9.99E-01	9.62E+02	
33	ARG	mtt85-mmm180°	-8.40	-20.91	4.12	7.00E-01	6.74E+02	
34	ARG	off-mmm180°	-12.05	-23.04	-1.07	1.45E-02	1.39E+01	
35	ARG	ptm-85°-mmm180°	1.81	-23.18	26.81	1.00E+00	9.63E+02	
36	ARG	ptm180°-mmm180°	-12.70	-31.87	6.46	7.22E-01	6.95E+02	
37	ARG	ptp180°-mmm180°	-17.96	-35.72	-0.19	4.40E-02	4.24E+01	
38	ARG	ptp85°-mmm180°	-4.75	-26.72	17.21	1.00E+00	9.63E+02	
39	ARG	ptt-85°-mmm180°	-10.06	-25.76	5.64	7.77E-01	7.49E+02	
40	ARG	ptt180°-mmm180°	-18.02	-33.43	-2.60	5.17E-03	4.98E+00	
41	ARG	ptt85°-mmm180°	-9.53	-24.55	5.48	7.90E-01	7.61E+02	
42	ARG	mmt180°-mmt-85°	12.33	0.60	24.07	2.65E-02	2.56E+01	
43	ARG	mmt85°-mmt-85°	15.46	0.82	30.10	2.51E-02	2.41E+01	
44	ARG	mtm-85°-mmt-85°	26.79	17.05	36.53	0.00E+00	0.00E+00	*

45	ARG	mtm105°-mmt-85°	9.78	-3.64	23.21	5.38E-01	5.18E+02	
46	ARG	mtm180°-mmt-85°	13.69	3.64	23.73	2.13E-04	2.05E-01	
47	ARG	mtp-105°-mmt-85°	20.71	6.27	35.15	5.69E-05	5.48E-02	
48	ARG	mtp180°-mmt-85°	15.87	5.74	26.00	4.10E-06	3.95E-03	*
49	ARG	mtp85°-mmt-85°	14.84	4.27	25.41	1.01E-04	9.70E-02	
50	ARG	mtt-85°-mmt-85°	15.51	5.70	25.32	3.03E-06	2.92E-03	*
51	ARG	mtt180°-mmt-85°	18.54	9.29	27.80	0.00E+00	0.00E+00	*
52	ARG	mtt85°-mmt-85°	14.71	4.50	24.91	5.03E-05	4.84E-02	*
53	ARG	off-mmt-85°	11.05	2.78	19.32	3.44E-04	3.31E-01	
54	ARG	ptm-85°-mmt-85°	24.92	0.99	48.84	3.01E-02	2.90E+01	
55	ARG	ptm180°-mmt-85°	10.40	-7.35	28.15	8.87E-01	8.54E+02	
56	ARG	ptp180°-mmt-85°	5.15	-11.08	21.37	1.00E+00	9.63E+02	
57	ARG	ptp85°-mmt-85°	18.35	-2.39	39.09	1.70E-01	1.64E+02	
58	ARG	ptt-85°-mmt-85°	13.04	-0.89	26.98	1.02E-01	9.84E+01	
59	ARG	ptt180°-mmt-85°	5.09	-8.52	18.70	9.99E-01	9.62E+02	
60	ARG	ptt85°-mmt-85°	13.57	0.41	26.72	3.41E-02	3.28E+01	
61	ARG	mmt85°-mmt180°	3.12	-11.81	18.06	1.00E+00	9.63E+02	
62	ARG	mtm-85°-mmt180°	14.46	4.29	24.63	7.28E-05	7.01E-02	
63	ARG	mtm105°-mmt180°	-2.55	-16.30	11.19	1.00E+00	9.63E+02	
64	ARG	mtm180°-mmt180°	1.35	-9.11	11.82	1.00E+00	9.63E+02	
65	ARG	mtp-105°-mmt180°	8.38	-6.36	23.11	9.13E-01	8.79E+02	
66	ARG	mtp180°-mmt180°	3.54	-7.01	14.09	1.00E+00	9.63E+02	
67	ARG	mtp85°-mmt180°	2.50	-8.47	13.47	1.00E+00	9.63E+02	
68	ARG	mtt-85°-mmt180°	3.18	-7.07	13.42	1.00E+00	9.63E+02	
69	ARG	mtt180°-mmt180°	6.21	-3.50	15.92	7.80E-01	7.51E+02	
70	ARG	mtt85°-mmt180°	2.37	-8.25	12.99	1.00E+00	9.63E+02	
71	ARG	off-mmt180°	-1.29	-10.06	7.49	1.00E+00	9.63E+02	
72	ARG	ptm-85°-mmt180°	12.58	-11.52	36.69	9.62E-01	9.26E+02	
73	ARG	ptm180°-mmt180°	-1.93	-19.92	16.06	1.00E+00	9.63E+02	
74	ARG	ptp180°-mmt180°	-7.19	-23.68	9.30	9.95E-01	9.59E+02	
75	ARG	ptp85°-mmt180°	6.01	-14.94	26.96	1.00E+00	9.63E+02	
76	ARG	ptt-85°-mmt180°	0.71	-13.53	14.95	1.00E+00	9.63E+02	
77	ARG	ptt180°-mmt180°	-7.25	-21.17	6.68	9.63E-01	9.27E+02	
78	ARG	ptt85°-mmt180°	1.23	-12.24	14.71	1.00E+00	9.63E+02	
79	ARG	mtm-85°-mmt85°	11.33	-2.09	24.76	2.43E-01	2.34E+02	
80	ARG	mtm105°-mmt85°	-5.68	-21.97	10.62	1.00E+00	9.63E+02	
81	ARG	mtm180°-mmt85°	-1.77	-15.42	11.88	1.00E+00	9.63E+02	
82	ARG	mtp-105°-mmt85°	5.25	-11.89	22.40	1.00E+00	9.63E+02	
83	ARG	mtp180°-mmt85°	0.41	-13.30	14.13	1.00E+00	9.63E+02	
84	ARG	mtp85°-mmt85°	-0.62	-14.66	13.42	1.00E+00	9.63E+02	
85	ARG	mtt-85°-mmt85°	0.05	-13.43	13.53	1.00E+00	9.63E+02	
86	ARG	mtt180°-mmt85°	3.08	-9.99	16.16	1.00E+00	9.63E+02	
87	ARG	mtt85°-mmt85°	-0.75	-14.52	13.02	1.00E+00	9.63E+02	
88	ARG	off-mmt85°	-4.41	-16.81	7.99	1.00E+00	9.63E+02	
89	ARG	ptm-85°-mmt85°	9.46	-16.19	35.11	1.00E+00	9.63E+02	
90	ARG	ptm180°-mmt85°	-5.06	-25.07	14.95	1.00E+00	9.63E+02	
91	ARG	ptp180°-mmt85°	-10.31	-28.98	8.36	9.33E-01	8.99E+02	
92	ARG	ptp85°-mmt85°	2.89	-19.82	25.60	1.00E+00	9.63E+02	

93	ARG	ptt-85°-mmt85°	-2.41	-19.13	14.31	1.00E+00	9.63E+02	
94	ARG	ptt180°-mmt85°	-10.37	-26.82	6.08	8.00E-01	7.71E+02	
95	ARG	ptt85°-mmt85°	-1.89	-17.97	14.18	1.00E+00	9.63E+02	
96	ARG	mtm105°-mtm-85°	-17.01	-29.09	-4.93	9.43E-05	9.08E-02	
97	ARG	mtm180°-mtm-85°	-13.10	-21.27	-4.94	1.79E-06	1.72E-03	*
98	ARG	mtp-105°-mtm-85°	-6.08	-19.28	7.12	9.91E-01	9.54E+02	
99	ARG	mtp180°-mtm-85°	-10.92	-19.20	-2.65	4.55E-04	4.38E-01	
100	ARG	mtp85°-mtm-85°	-11.95	-20.76	-3.15	2.33E-04	2.24E-01	
101	ARG	mtt-85°-mtm-85°	-11.28	-19.16	-3.40	6.01E-05	5.79E-02	
102	ARG	mtt180°-mtm-85°	-8.25	-15.42	-1.08	6.83E-03	6.57E+00	
103	ARG	mtt85°-mtm-85°	-12.08	-20.45	-3.72	4.69E-05	4.52E-02	*
104	ARG	off-mtm-85°	-15.74	-21.59	-9.89	0.00E+00	0.00E+00	*
105	ARG	ptm-85°-mtm-85°	-1.87	-25.07	21.33	1.00E+00	9.63E+02	
106	ARG	ptm180°-mtm-85°	-16.39	-33.15	0.37	6.44E-02	6.20E+01	
107	ARG	ptp180°-mtm-85°	-21.64	-36.78	-6.51	6.18E-05	5.95E-02	
108	ARG	ptp85°-mtm-85°	-8.44	-28.34	11.46	9.97E-01	9.60E+02	
109	ARG	ptt-85°-mtm-85°	-13.75	-26.39	-1.10	1.66E-02	1.60E+01	
110	ARG	ptt180°-mtm-85°	-21.70	-33.99	-9.42	0.00E+00	0.00E+00	*
111	ARG	ptt85°-mtm-85°	-13.22	-25.00	-1.44	1.02E-02	9.81E+00	
112	ARG	mtm180°-mtm105°	3.91	-8.43	16.24	1.00E+00	9.63E+02	
113	ARG	mtp-105°-mtm105°	10.93	-5.19	27.05	6.81E-01	6.56E+02	
114	ARG	mtp180°-mtm105°	6.09	-6.32	18.49	9.80E-01	9.44E+02	
115	ARG	mtp85°-mtm105°	5.06	-7.71	17.82	9.99E-01	9.62E+02	
116	ARG	mtt-85°-mtm105°	5.73	-6.42	17.87	9.88E-01	9.51E+02	
117	ARG	mtt180°-mtm105°	8.76	-2.94	20.46	4.80E-01	4.63E+02	
118	ARG	mtt85°-mtm105°	4.93	-7.54	17.39	9.99E-01	9.62E+02	
119	ARG	off-mtm105°	1.27	-9.67	12.21	1.00E+00	9.63E+02	
120	ARG	ptm-85°-mtm105°	15.14	-9.84	40.11	8.51E-01	8.20E+02	
121	ARG	ptm180°-mtm105°	0.62	-18.52	19.75	1.00E+00	9.63E+02	
122	ARG	ptp180°-mtm105°	-4.63	-22.37	13.10	1.00E+00	9.63E+02	
123	ARG	ptp85°-mtm105°	8.57	-13.38	30.51	9.99E-01	9.62E+02	
124	ARG	ptt-85°-mtm105°	3.26	-12.40	18.93	1.00E+00	9.63E+02	
125	ARG	ptt180°-mtm105°	-4.70	-20.07	10.68	1.00E+00	9.63E+02	
126	ARG	ptt85°-mtm105°	3.79	-11.19	18.76	1.00E+00	9.63E+02	
127	ARG	mtp-105°-mtm180°	7.02	-6.41	20.45	9.61E-01	9.25E+02	
128	ARG	mtp180°-mtm180°	2.18	-6.45	10.82	1.00E+00	9.63E+02	
129	ARG	mtp85°-mtm180°	1.15	-7.99	10.29	1.00E+00	9.63E+02	
130	ARG	mtt-85°-mtm180°	1.82	-6.44	10.08	1.00E+00	9.63E+02	
131	ARG	mtt180°-mtm180°	4.85	-2.73	12.44	7.78E-01	7.50E+02	
132	ARG	mtt85°-mtm180°	1.02	-7.70	9.74	1.00E+00	9.63E+02	
133	ARG	off-mtm180°	-2.64	-8.99	3.71	9.98E-01	9.61E+02	
134	ARG	ptm-85°-mtm180°	11.23	-12.10	34.56	9.84E-01	9.48E+02	
135	ARG	ptm180°-mtm180°	-3.29	-20.23	13.65	1.00E+00	9.63E+02	
136	ARG	ptp180°-mtm180°	-8.54	-23.88	6.79	9.28E-01	8.93E+02	
137	ARG	ptp85°-mtm180°	4.66	-15.39	24.71	1.00E+00	9.63E+02	
138	ARG	ptt-85°-mtm180°	-0.64	-13.53	12.24	1.00E+00	9.63E+02	
139	ARG	ptt180°-mtm180°	-8.60	-21.14	3.93	6.59E-01	6.34E+02	
140	ARG	ptt85°-mtm180°	-0.12	-12.16	11.92	1.00E+00	9.63E+02	

141	ARG	mtp180°-mtp-105°	-4.84	-18.34	8.65	1.00E+00	9.63E+02	
142	ARG	mtp85°-mtp-105°	-5.87	-19.70	7.95	9.97E-01	9.60E+02	
143	ARG	mtt-85°-mtp-105°	-5.20	-18.46	8.06	9.99E-01	9.62E+02	
144	ARG	mtt180°-mtp-105°	-2.17	-15.02	10.68	1.00E+00	9.63E+02	
145	ARG	mtt85°-mtp-105°	-6.00	-19.56	7.55	9.94E-01	9.58E+02	
146	ARG	off-mtp-105°	-9.66	-21.83	2.50	3.57E-01	3.44E+02	
147	ARG	ptm-85°-mtp-105°	4.21	-21.33	29.74	1.00E+00	9.63E+02	
148	ARG	ptm180°-mtp-105°	-10.31	-30.17	9.55	9.64E-01	9.28E+02	
149	ARG	ptp180°-mtp-105°	-15.57	-34.08	2.95	2.50E-01	2.41E+02	
150	ARG	ptp85°-mtp-105°	-2.36	-24.94	20.21	1.00E+00	9.63E+02	
151	ARG	ptt-85°-mtp-105°	-7.67	-24.21	8.88	9.90E-01	9.53E+02	
152	ARG	ptt180°-mtp-105°	-15.63	-31.89	0.64	7.86E-02	7.57E+01	
153	ARG	ptt85°-mtp-105°	-7.14	-23.03	8.75	9.93E-01	9.56E+02	
154	ARG	mtp85°-mtp180°	-1.03	-10.27	8.21	1.00E+00	9.63E+02	
155	ARG	mtt-85°-mtp180°	-0.36	-8.73	8.00	1.00E+00	9.63E+02	
156	ARG	mtt180°-mtp180°	2.67	-5.03	10.37	1.00E+00	9.63E+02	
157	ARG	mtt85°-mtp180°	-1.16	-9.99	7.66	1.00E+00	9.63E+02	
158	ARG	off-mtp180°	-4.82	-11.31	1.67	4.96E-01	4.78E+02	
159	ARG	ptm-85°-mtp180°	9.05	-14.32	32.42	9.99E-01	9.62E+02	
160	ARG	ptm180°-mtp180°	-5.47	-22.46	11.52	1.00E+00	9.63E+02	
161	ARG	ptp180°-mtp180°	-10.72	-26.12	4.67	6.30E-01	6.06E+02	
162	ARG	ptp85°-mtp180°	2.48	-17.62	22.57	1.00E+00	9.63E+02	
163	ARG	ptt-85°-mtp180°	-2.83	-15.78	10.13	1.00E+00	9.63E+02	
164	ARG	ptt180°-mtp180°	-10.78	-23.39	1.82	2.21E-01	2.13E+02	
165	ARG	ptt85°-mtp180°	-2.30	-14.41	9.81	1.00E+00	9.63E+02	
166	ARG	mtt-85°-mtp85°	0.67	-8.22	9.56	1.00E+00	9.63E+02	
167	ARG	mtt180°-mtp85°	3.71	-4.56	11.97	9.93E-01	9.57E+02	
168	ARG	mtt85°-mtp85°	-0.13	-9.45	9.19	1.00E+00	9.63E+02	
169	ARG	off-mtp85°	-3.79	-10.94	3.36	9.55E-01	9.20E+02	
170	ARG	ptm-85°-mtp85°	10.08	-13.48	33.64	9.96E-01	9.60E+02	
171	ARG	ptm180°-mtp85°	-4.44	-21.69	12.81	1.00E+00	9.63E+02	
172	ARG	ptp180°-mtp85°	-9.69	-25.37	5.99	8.28E-01	7.97E+02	
173	ARG	ptp85°-mtp85°	3.51	-16.81	23.83	1.00E+00	9.63E+02	
174	ARG	ptt-85°-mtp85°	-1.79	-15.09	11.51	1.00E+00	9.63E+02	
175	ARG	ptt180°-mtp85°	-9.75	-22.71	3.21	4.70E-01	4.53E+02	
176	ARG	ptt85°-mtp85°	-1.27	-13.75	11.21	1.00E+00	9.63E+02	
177	ARG	mtt180°-mtt-85°	3.03	-4.24	10.31	9.97E-01	9.61E+02	
178	ARG	mtt85°-mtt-85°	-0.80	-9.26	7.65	1.00E+00	9.63E+02	
179	ARG	off-mtt-85°	-4.46	-10.44	1.52	4.87E-01	4.69E+02	
180	ARG	ptm-85°-mtt-85°	9.41	-13.82	32.64	9.98E-01	9.61E+02	
181	ARG	ptm180°-mtt-85°	-5.11	-21.91	11.69	1.00E+00	9.63E+02	
182	ARG	ptp180°-mtt-85°	-10.36	-25.55	4.82	6.69E-01	6.45E+02	
183	ARG	ptp85°-mtt-85°	2.84	-17.10	22.78	1.00E+00	9.63E+02	
184	ARG	ptt-85°-mtt-85°	-2.46	-15.17	10.24	1.00E+00	9.63E+02	
185	ARG	ptt180°-mtt-85°	-10.42	-22.77	1.93	2.44E-01	2.35E+02	
186	ARG	ptt85°-mtt-85°	-1.94	-13.79	9.90	1.00E+00	9.63E+02	
187	ARG	mtt85°-mtt180°	-3.84	-11.63	3.96	9.80E-01	9.44E+02	
188	ARG	off-mtt180°	-7.49	-12.50	-2.49	1.67E-05	1.61E-02	*

189	ARG	ptm-85°-mtt180°	6.37	-16.63	29.38	1.00E+00	9.63E+02	
190	ARG	ptm180°-mtt180°	-8.14	-24.62	8.34	9.79E-01	9.43E+02	
191	ARG	ptp180°-mtt180°	-13.40	-28.22	1.43	1.42E-01	1.37E+02	
192	ARG	ptp85°-mtt180°	-0.20	-19.86	19.47	1.00E+00	9.63E+02	
193	ARG	ptt-85°-mtt180°	-5.50	-17.78	6.78	9.93E-01	9.57E+02	
194	ARG	ptt180°-mtt180°	-13.46	-25.37	-1.55	9.13E-03	8.80E+00	
195	ARG	ptt85°-mtt180°	-4.97	-16.36	6.41	9.95E-01	9.58E+02	
196	ARG	off-mtt85°	-3.66	-10.26	2.94	9.31E-01	8.97E+02	
197	ARG	ptm-85°-mtt85°	10.21	-13.19	33.61	9.95E-01	9.59E+02	
198	ARG	ptm180°-mtt85°	-4.31	-21.34	12.73	1.00E+00	9.63E+02	
199	ARG	ptp180°-mtt85°	-9.56	-25.00	5.88	8.25E-01	7.95E+02	
200	ARG	ptp85°-mtt85°	3.64	-16.49	23.77	1.00E+00	9.63E+02	
201	ARG	ptt-85°-mtt85°	-1.66	-14.68	11.35	1.00E+00	9.63E+02	
202	ARG	ptt180°-mtt85°	-9.62	-22.28	3.04	4.50E-01	4.33E+02	
203	ARG	ptt85°-mtt85°	-1.14	-13.31	11.03	1.00E+00	9.63E+02	
204	ARG	ptm-85°-off	13.87	-8.76	36.49	8.38E-01	8.07E+02	
205	ARG	ptm180°-off	-0.65	-16.60	15.30	1.00E+00	9.63E+02	
206	ARG	ptp180°-off	-5.90	-20.14	8.33	9.98E-01	9.61E+02	
207	ARG	ptp85°-off	7.30	-11.93	26.53	9.99E-01	9.62E+02	
208	ARG	ptt-85°-off	2.00	-9.56	13.55	1.00E+00	9.63E+02	
209	ARG	ptt180°-off	-5.96	-17.12	5.20	9.52E-01	9.17E+02	
210	ARG	ptt85°-off	2.52	-8.08	13.12	1.00E+00	9.63E+02	
211	ARG	ptm180°-ptm-85°	-14.52	-42.06	13.02	9.58E-01	9.22E+02	
212	ARG	ptp180°-ptm-85°	-19.77	-46.35	6.81	4.95E-01	4.77E+02	
213	ARG	ptp85°-ptm-85°	-6.57	-36.13	22.99	1.00E+00	9.63E+02	
214	ARG	ptt-85°-ptm-85°	-11.87	-37.12	13.38	9.88E-01	9.52E+02	
215	ARG	ptt180°-ptm-85°	-19.83	-44.90	5.24	3.66E-01	3.53E+02	
216	ARG	ptt85°-ptm-85°	-11.35	-36.18	13.48	9.92E-01	9.55E+02	
217	ARG	ptp180°-ptm180°	-5.25	-26.45	15.94	1.00E+00	9.63E+02	
218	ARG	ptp85°-ptm180°	7.95	-16.87	32.77	1.00E+00	9.63E+02	
219	ARG	ptt-85°-ptm180°	2.65	-16.85	22.14	1.00E+00	9.63E+02	
220	ARG	ptt180°-ptm180°	-5.31	-24.58	13.95	1.00E+00	9.63E+02	
221	ARG	ptt85°-ptm180°	3.17	-15.78	22.12	1.00E+00	9.63E+02	
222	ARG	ptp85°-ptp180°	13.20	-10.56	36.96	9.29E-01	8.95E+02	
223	ARG	ptt-85°-ptp180°	7.90	-10.23	26.02	9.95E-01	9.59E+02	
224	ARG	ptt180°-ptp180°	-0.06	-17.93	17.81	1.00E+00	9.63E+02	
225	ARG	ptt85°-ptp180°	8.42	-9.11	25.95	9.85E-01	9.48E+02	
226	ARG	ptt-85°-ptp85°	-5.30	-27.56	16.95	1.00E+00	9.63E+02	
227	ARG	ptt180°-ptp85°	-13.26	-35.32	8.79	8.60E-01	8.29E+02	
228	ARG	ptt85°-ptp85°	-4.78	-26.56	17.00	1.00E+00	9.63E+02	
229	ARG	ptt180°-ptt-85°	-7.96	-23.78	7.87	9.74E-01	9.38E+02	
230	ARG	ptt85°-ptt-85°	0.52	-14.91	15.96	1.00E+00	9.63E+02	
231	ARG	ptt85°-ptt180°	8.48	-6.66	23.62	9.24E-01	8.90E+02	
232	ASN	m-80°-m-20°	-14.10	-17.36	-10.84	0.00E+00	0.00E+00	*
233	ASN	m120°-m-20°	1.81	-1.40	5.01	6.81E-01	6.56E+02	
234	ASN	off-m-20°	-12.07	-15.31	-8.83	0.00E+00	0.00E+00	*
235	ASN	p-10°-m-20°	-11.93	-15.98	-7.89	0.00E+00	0.00E+00	*
236	ASN	p30°-m-20°	-14.81	-18.44	-11.18	0.00E+00	0.00E+00	*

237	ASN	t-20°-m-20°	-21.25	-24.17	-18.32	0.00E+00	0.00E+00	*
238	ASN	t30°-m-20°	-6.26	-9.15	-3.38	0.00E+00	0.00E+00	*
239	ASN	m120°-m-80°	15.91	11.89	19.92	0.00E+00	0.00E+00	*
240	ASN	off-m-80°	2.03	-2.01	6.07	7.96E-01	7.66E+02	
241	ASN	p-10°-m-80°	2.17	-2.55	6.89	8.60E-01	8.28E+02	
242	ASN	p30°-m-80°	-0.71	-5.07	3.66	1.00E+00	9.63E+02	
243	ASN	t-20°-m-80°	-7.14	-10.94	-3.35	2.18E-07	2.10E-04	*
244	ASN	t30°-m-80°	7.84	4.07	11.61	0.00E+00	0.00E+00	*
245	ASN	off-m120°	-13.88	-17.87	-9.88	0.00E+00	0.00E+00	*
246	ASN	p-10°-m120°	-13.74	-18.41	-9.06	0.00E+00	0.00E+00	*
247	ASN	p30°-m120°	-16.61	-20.93	-12.30	0.00E+00	0.00E+00	*
248	ASN	t-20°-m120°	-23.05	-26.80	-19.30	0.00E+00	0.00E+00	*
249	ASN	t30°-m120°	-8.07	-11.78	-4.35	0.00E+00	0.00E+00	*
250	ASN	p-10°-off	0.14	-4.56	4.84	1.00E+00	9.63E+02	
251	ASN	p30°-off	-2.74	-7.08	1.61	5.43E-01	5.23E+02	
252	ASN	t-20°-off	-9.18	-12.95	-5.40	0.00E+00	0.00E+00	*
253	ASN	t30°-off	5.81	2.06	9.56	7.15E-05	6.88E-02	
254	ASN	p30°-p-10°	-2.88	-7.85	2.10	6.52E-01	6.28E+02	
255	ASN	t-20°-p-10°	-9.31	-13.80	-4.82	0.00E+00	0.00E+00	*
256	ASN	t30°-p-10°	5.67	1.21	10.13	2.98E-03	2.87E+00	
257	ASN	t-20°-p30°	-6.44	-10.55	-2.32	5.92E-05	5.70E-02	
258	ASN	t30°-p30°	8.55	4.46	12.64	0.00E+00	0.00E+00	*
259	ASN	t30°-t-20°	14.98	11.50	18.46	0.00E+00	0.00E+00	*
260	ASP	off-m-20°	-14.83	-19.28	-10.37	5.15E-14	4.96E-11	*
261	ASP	p-10°-m-20°	-2.58	-5.08	-0.07	3.96E-02	3.81E+01	
262	ASP	p30°-m-20°	-10.76	-13.27	-8.25	0.00E+00	0.00E+00	*
263	ASP	t0°-m-20°	-17.37	-19.07	-15.66	0.00E+00	0.00E+00	*
264	ASP	t70°-m-20°	-17.83	-20.32	-15.33	0.00E+00	0.00E+00	*
265	ASP	p-10°-off	12.25	7.32	17.18	2.08E-11	2.00E-08	*
266	ASP	p30°-off	4.07	-0.86	9.00	1.74E-01	1.67E+02	
267	ASP	t0°-off	-2.54	-7.11	2.03	6.10E-01	5.87E+02	
268	ASP	t70°-off	-3.00	-7.92	1.92	5.07E-01	4.88E+02	
269	ASP	p30°-p-10°	-8.18	-11.46	-4.90	1.70E-11	1.64E-08	*
270	ASP	t0°-p-10°	-14.79	-17.49	-12.08	0.00E+00	0.00E+00	*
271	ASP	t70°-p-10°	-15.25	-18.51	-11.98	0.00E+00	0.00E+00	*
272	ASP	t0°-p30°	-6.61	-9.32	-3.89	5.88E-11	5.66E-08	*
273	ASP	t70°-p30°	-7.07	-10.34	-3.80	1.10E-08	1.06E-05	*
274	ASP	t70°-t0°	-0.46	-3.16	2.24	9.97E-01	9.60E+02	
275	CYS	off-m	1.54	-5.93	9.00	9.52E-01	9.17E+02	
276	CYS	p-m	2.47	0.78	4.16	9.84E-04	9.47E-01	
277	CYS	t-m	-1.04	-2.46	0.38	2.38E-01	2.29E+02	
278	CYS	p-off	0.94	-6.63	8.50	9.89E-01	9.52E+02	
279	CYS	t-off	-2.57	-10.08	4.94	8.15E-01	7.85E+02	
280	CYS	t-p	-3.51	-5.40	-1.62	1.04E-05	1.00E-02	*
281	GLN	mm100°-mm-40°	1.78	-4.41	7.98	9.96E-01	9.59E+02	
282	GLN	mp0°-mm-40°	-10.00	-17.88	-2.11	2.45E-03	2.36E+00	
283	GLN	mt-30°-mm-40°	-1.34	-5.27	2.59	9.87E-01	9.50E+02	
284	GLN	off-mm-40°	-3.21	-7.42	1.00	3.18E-01	3.06E+02	

285	GLN	pm0°-mm-40°	-9.90	-21.72	1.92	1.95E-01	1.88E+02	
286	GLN	pt20°-mm-40°	-5.07	-11.94	1.80	3.65E-01	3.52E+02	
287	GLN	tp-100°-mm-40°	-13.78	-22.14	-5.42	8.16E-06	7.86E-03	*
288	GLN	tp60°-mm-40°	-5.09	-10.49	0.31	8.50E-02	8.18E+01	
289	GLN	tt0°-mm-40°	-4.21	-8.70	0.29	8.95E-02	8.62E+01	
290	GLN	mp0°-mm100°	-11.78	-20.64	-2.92	1.08E-03	1.04E+00	
291	GLN	mt-30°-mm100°	-3.12	-8.76	2.52	7.65E-01	7.37E+02	
292	GLN	off-mm100°	-5.00	-10.84	0.84	1.71E-01	1.64E+02	
293	GLN	pm0°-mm100°	-11.68	-24.18	0.81	9.02E-02	8.68E+01	
294	GLN	pt20°-mm100°	-6.85	-14.82	1.12	1.65E-01	1.59E+02	
295	GLN	tp-100°-mm100°	-15.57	-24.85	-6.28	5.12E-06	4.93E-03	*
296	GLN	tp60°-mm100°	-6.87	-13.62	-0.12	4.19E-02	4.04E+01	
297	GLN	tt0°-mm100°	-5.99	-12.04	0.06	5.47E-02	5.26E+01	
298	GLN	mt-30°-mp0°	8.66	1.20	16.11	9.01E-03	8.67E+00	
299	GLN	off-mp0°	6.78	-0.82	14.39	1.29E-01	1.24E+02	
300	GLN	pm0°-mp0°	0.10	-13.32	13.51	1.00E+00	9.63E+02	
301	GLN	pt20°-mp0°	4.93	-4.41	14.27	8.13E-01	7.83E+02	
302	GLN	tp-100°-mp0°	-3.79	-14.27	6.70	9.80E-01	9.44E+02	
303	GLN	tp60°-mp0°	4.91	-3.42	13.23	6.93E-01	6.67E+02	
304	GLN	tt0°-mp0°	5.79	-1.98	13.56	3.51E-01	3.38E+02	
305	GLN	off-mt-30°	-1.87	-5.21	1.46	7.49E-01	7.22E+02	
306	GLN	pm0°-mt-30°	-8.56	-20.10	2.98	3.58E-01	3.45E+02	
307	GLN	pt20°-mt-30°	-3.73	-10.10	2.64	7.00E-01	6.74E+02	
308	GLN	tp-100°-mt-30°	-12.44	-20.40	-4.49	3.27E-05	3.15E-02	*
309	GLN	tp60°-mt-30°	-3.75	-8.50	1.00	2.70E-01	2.60E+02	
310	GLN	tt0°-mt-30°	-2.87	-6.55	0.82	2.89E-01	2.78E+02	
311	GLN	pm0°-off	-6.69	-18.33	4.95	7.24E-01	6.97E+02	
312	GLN	pt20°-off	-1.86	-8.40	4.69	9.97E-01	9.60E+02	
313	GLN	tp-100°-off	-10.57	-18.67	-2.48	1.49E-03	1.43E+00	
314	GLN	tp60°-off	-1.88	-6.86	3.11	9.74E-01	9.38E+02	
315	GLN	tt0°-off	-0.99	-4.98	2.99	9.99E-01	9.62E+02	
316	GLN	pt20°-pm0°	4.83	-8.01	17.67	9.74E-01	9.38E+02	
317	GLN	tp-100°-pm0°	-3.88	-17.58	9.81	9.97E-01	9.60E+02	
318	GLN	tp60°-pm0°	4.81	-7.31	16.93	9.63E-01	9.27E+02	
319	GLN	tt0°-pm0°	5.69	-6.05	17.44	8.79E-01	8.46E+02	
320	GLN	tp-100°-pt20°	-8.71	-18.46	1.03	1.27E-01	1.22E+02	
321	GLN	tp60°-pt20°	-0.02	-7.39	7.35	1.00E+00	9.63E+02	
322	GLN	tt0°-pt20°	0.86	-5.87	7.59	1.00E+00	9.63E+02	
323	GLN	tp60°-tp-100°	8.69	-0.08	17.47	5.46E-02	5.25E+01	
324	GLN	tt0°-tp-100°	9.58	1.33	17.82	9.06E-03	8.72E+00	
325	GLN	tt0°-tp60°	0.88	-4.35	6.11	1.00E+00	9.63E+02	
326	GLU	mp0°-mm-40°	0.16	-4.17	4.49	1.00E+00	9.63E+02	
327	GLU	mt-10°-mm-40°	2.07	-0.70	4.84	3.34E-01	3.22E+02	
328	GLU	off-mm-40°	-2.68	-7.11	1.75	6.32E-01	6.09E+02	
329	GLU	pm0°-mm-40°	0.65	-5.71	7.01	1.00E+00	9.63E+02	
330	GLU	pt-20°-mm-40°	-0.76	-5.33	3.81	1.00E+00	9.63E+02	
331	GLU	tm-20°-mm-40°	-10.18	-18.60	-1.76	5.58E-03	5.38E+00	
332	GLU	tp10°-mm-40°	-8.55	-12.64	-4.46	3.21E-09	3.10E-06	*

333	GLU	tt0°-mm-40°	-1.13	-4.11	1.85	9.62E-01	9.26E+02	
334	GLU	mt-10°-mp0°	1.91	-2.03	5.85	8.55E-01	8.23E+02	
335	GLU	off-mp0°	-2.83	-8.08	2.41	7.61E-01	7.33E+02	
336	GLU	pm0°-mp0°	0.49	-6.46	7.44	1.00E+00	9.63E+02	
337	GLU	pt-20°-mp0°	-0.92	-6.28	4.44	1.00E+00	9.63E+02	
338	GLU	tm-20°-mp0°	-10.34	-19.22	-1.46	9.27E-03	8.93E+00	
339	GLU	tp10°-mp0°	-8.71	-13.67	-3.75	1.83E-06	1.76E-03	*
340	GLU	tt0°-mp0°	-1.29	-5.38	2.80	9.88E-01	9.52E+02	
341	GLU	off-mt-10°	-4.75	-8.80	-0.69	8.70E-03	8.37E+00	
342	GLU	pm0°-mt-10°	-1.42	-7.52	4.69	9.99E-01	9.62E+02	
343	GLU	pt-20°-mt-10°	-2.83	-7.04	1.37	4.81E-01	4.64E+02	
344	GLU	tm-20°-mt-10°	-12.25	-20.48	-4.01	1.37E-04	1.32E-01	
345	GLU	tp10°-mt-10°	-10.62	-14.30	-6.94	6.22E-14	5.99E-11	*
346	GLU	tt0°-mt-10°	-3.20	-5.58	-0.81	1.06E-03	1.02E+00	
347	GLU	pm0°-off	3.33	-3.69	10.34	8.69E-01	8.37E+02	
348	GLU	pt-20°-off	1.91	-3.53	7.36	9.76E-01	9.40E+02	
349	GLU	tm-20°-off	-7.50	-16.43	1.43	1.84E-01	1.77E+02	
350	GLU	tp10°-off	-5.88	-10.92	-0.83	9.36E-03	9.01E+00	
351	GLU	tt0-off	1.55	-2.65	5.75	9.68E-01	9.32E+02	
352	GLU	pt-20°-pm0°	-1.41	-8.52	5.69	1.00E+00	9.63E+02	
353	GLU	tm-20°-pm0°	-10.83	-20.86	-0.80	2.29E-02	2.21E+01	
354	GLU	tp10°-pm0°	-9.20	-16.01	-2.40	9.17E-04	8.83E-01	
355	GLU	tt0°-pm0°	-1.78	-7.98	4.42	9.94E-01	9.57E+02	
356	GLU	tm-20°-pt-20°	-9.42	-18.41	-0.42	3.22E-02	3.10E+01	
357	GLU	tp10°-pt-20°	-7.79	-12.96	-2.62	1.05E-04	1.01E-01	
358	GLU	tt0°-pt-20°	-0.36	-4.71	3.98	1.00E+00	9.63E+02	
359	GLU	tp10°-tm-20°	1.63	-7.14	10.39	1.00E+00	9.63E+02	
360	GLU	tt0°-tm-20°	9.05	0.75	17.35	2.07E-02	2.00E+01	
361	GLU	tt0°-tp10°	7.42	3.58	11.26	7.14E-08	6.88E-05	*
362	HIS	m170°-m-70°	-2.58	-7.32	2.17	7.23E-01	6.96E+02	
363	HIS	m80°-m-70°	-2.63	-6.67	1.41	5.00E-01	4.81E+02	
364	HIS	off-m-70°	-11.90	-18.19	-5.61	2.74E-07	2.64E-04	*
365	HIS	p-80°-m-70°	-7.66	-12.89	-2.42	2.47E-04	2.38E-01	
366	HIS	p80°-m-70°	-12.69	-18.72	-6.66	4.27E-09	4.11E-06	*
367	HIS	t-160°-m-70°	-18.86	-25.27	-12.44	0.00E+00	0.00E+00	*
368	HIS	t-80°-m-70°	-6.09	-9.33	-2.86	3.20E-07	3.08E-04	*
369	HIS	m80°-m170°	-0.05	-5.38	5.28	1.00E+00	9.63E+02	
370	HIS	off-m170°	-9.32	-16.51	-2.14	2.14E-03	2.07E+00	
371	HIS	p-80°-m170°	-5.08	-11.36	1.20	2.17E-01	2.09E+02	
372	HIS	p80°-m170°	-10.12	-17.08	-3.15	2.86E-04	2.75E-01	
373	HIS	t-160°-m170°	-16.28	-23.58	-8.98	0.00E+00	0.00E+00	*
374	HIS	t-80°-m170°	-3.52	-8.27	1.23	3.25E-01	3.13E+02	
375	HIS	off-m80°	-9.27	-16.01	-2.53	8.07E-04	7.77E-01	
376	HIS	p-80°-m80°	-5.03	-10.79	0.74	1.41E-01	1.36E+02	
377	HIS	p80°-m80°	-10.06	-16.56	-3.56	7.47E-05	7.19E-02	
378	HIS	t-160°-m80°	-16.23	-23.09	-9.37	0.00E+00	0.00E+00	*
379	HIS	t-80°-m80°	-3.46	-7.51	0.58	1.57E-01	1.51E+02	
380	HIS	p-80°-off	4.25	-3.27	11.76	6.79E-01	6.54E+02	

381	HIS	p80°-off	-0.79	-8.89	7.30	1.00E+00	9.63E+02	
382	HIS	t-160°-off	-6.96	-15.34	1.43	1.89E-01	1.82E+02	
383	HIS	t-80°-off	5.81	-0.49	12.10	9.56E-02	9.21E+01	
384	HIS	p80°-p-80°	-5.04	-12.34	2.27	4.21E-01	4.05E+02	
385	HIS	t-160°-p-80°	-11.20	-18.82	-3.58	2.28E-04	2.19E-01	
386	HIS	t-80°-p-80°	1.56	-3.67	6.80	9.86E-01	9.49E+02	
387	HIS	t-160°-p80°	-6.16	-14.36	2.03	3.04E-01	2.93E+02	
388	HIS	t-80°-p80°	6.60	0.56	12.63	2.08E-02	2.00E+01	
389	HIS	t-80°-t-160°	12.76	6.34	19.18	4.72E-08	4.55E-05	*
390	ILE	mp-mm	-5.58	-10.76	-0.39	2.50E-02	2.41E+01	
391	ILE	mt-mm	-2.78	-4.25	-1.32	2.41E-07	2.32E-04	*
392	ILE	off-mm	10.34	5.82	14.86	1.10E-10	1.06E-07	*
393	ILE	pp-mm	8.14	0.90	15.37	1.50E-02	1.45E+01	
394	ILE	pt-mm	4.54	2.57	6.50	7.62E-11	7.33E-08	*
395	ILE	tp-mm	3.83	0.38	7.27	1.72E-02	1.66E+01	
396	ILE	tt-mm	-1.83	-4.35	0.70	3.55E-01	3.42E+02	
397	ILE	mt-mp	2.79	-2.27	7.86	7.06E-01	6.80E+02	
398	ILE	off-mp	15.92	9.29	22.54	9.54E-12	9.19E-09	*
399	ILE	pp-mp	13.71	5.00	22.42	4.98E-05	4.80E-02	*
400	ILE	pt-mp	10.11	4.88	15.35	1.34E-07	1.29E-04	*
401	ILE	tp-mp	9.40	3.45	15.35	4.55E-05	4.38E-02	*
402	ILE	tt-mp	3.75	-1.72	9.22	4.29E-01	4.13E+02	
403	ILE	off-mt	13.12	8.75	17.50	1.02E-13	9.81E-11	*
404	ILE	pp-mt	10.92	3.77	18.07	9.87E-05	9.50E-02	
405	ILE	pt-mt	7.32	5.70	8.94	0.00E+00	0.00E+00	*
406	ILE	tp-mt	6.61	3.35	9.87	2.18E-08	2.10E-05	*
407	ILE	tt-mt	0.96	-1.31	3.22	9.06E-01	8.73E+02	
408	ILE	pp-off	-2.20	-10.53	6.12	9.93E-01	9.56E+02	
409	ILE	pt-off	-5.80	-10.37	-1.24	2.96E-03	2.85E+00	
410	ILE	tp-off	-6.51	-11.88	-1.14	5.87E-03	5.65E+00	
411	ILE	tt-off	-12.17	-17.00	-7.33	7.15E-13	6.88E-10	*
412	ILE	pt-pp	-3.60	-10.87	3.66	8.07E-01	7.77E+02	
413	ILE	tp-pp	-4.31	-12.10	3.49	7.04E-01	6.78E+02	
414	ILE	tt-pp	-9.96	-17.40	-2.53	1.27E-03	1.22E+00	
415	ILE	tp-pt	-0.71	-4.22	2.81	9.99E-01	9.62E+02	
416	ILE	tt-pt	-6.36	-8.98	-3.75	4.66E-12	4.49E-09	*
417	ILE	tt-tp	-5.66	-9.51	-1.80	2.31E-04	2.22E-01	
418	LEU	mt-mp	7.17	4.49	9.86	4.18E-13	4.02E-10	*
419	LEU	off-mp	6.73	3.45	10.00	6.98E-08	6.72E-05	*
420	LEU	pp-mp	-2.19	-7.71	3.34	8.70E-01	8.38E+02	
421	LEU	tp-mp	1.46	-1.28	4.20	6.54E-01	6.30E+02	
422	LEU	tt-mp	-3.12	-7.02	0.78	2.03E-01	1.96E+02	
423	LEU	off-mt	-0.45	-2.46	1.57	9.89E-01	9.52E+02	
424	LEU	pp-mt	-9.36	-14.25	-4.47	7.38E-07	7.11E-04	*
425	LEU	tp-mt	-5.72	-6.65	-4.79	0.00E+00	0.00E+00	*
426	LEU	tt-mt	-10.29	-13.22	-7.36	4.71E-14	4.53E-11	*
427	LEU	pp-off	-8.91	-14.15	-3.68	1.83E-05	1.76E-02	*
428	LEU	tp-off	-5.27	-7.36	-3.18	1.05E-11	1.01E-08	*

429	LEU	tt-off	-9.84	-13.32	-6.37	8.78E-14	8.46E-11	*
430	LEU	tp-pp	3.64	-1.28	8.56	2.83E-01	2.72E+02	
431	LEU	tt-pp	-0.93	-6.59	4.72	9.97E-01	9.60E+02	
432	LEU	tt-tp	-4.57	-7.56	-1.59	1.81E-04	1.74E-01	
433	LYS	mmtm-mmmt	5.21	-7.71	18.13	1.00E+00	9.63E+02	
434	LYS	mmtp-mmmt	12.99	-1.21	27.19	1.34E-01	1.29E+02	
435	LYS	mmtt-mmmt	4.42	-6.40	15.24	1.00E+00	9.63E+02	
436	LYS	mptt-mmmt	4.79	-18.89	28.47	1.00E+00	9.63E+02	
437	LYS	mtmm-mmmt	-0.13	-14.89	14.63	1.00E+00	9.63E+02	
438	LYS	mtmt-mmmt	4.15	-7.64	15.94	1.00E+00	9.63E+02	
439	LYS	mtpp-mmmt	5.68	-9.27	20.62	1.00E+00	9.63E+02	
440	LYS	mtpt-mmmt	5.41	-6.36	17.17	9.97E-01	9.60E+02	
441	LYS	mttm-mmmt	12.65	1.43	23.87	8.51E-03	8.20E+00	
442	LYS	mttp-mmmt	16.23	4.61	27.86	7.71E-05	7.43E-02	
443	LYS	mttt-mmmt	13.96	3.74	24.17	1.40E-04	1.34E-01	
444	LYS	off-mmmt	15.12	4.74	25.50	2.30E-05	2.22E-02	*
445	LYS	ptmt-mmmt	10.71	-8.96	30.38	9.67E-01	9.31E+02	
446	LYS	ptpt-mmmt	3.20	-17.16	23.55	1.00E+00	9.63E+02	
447	LYS	pttm-mmmt	2.48	-14.99	19.95	1.00E+00	9.63E+02	
448	LYS	pttp-mmmt	4.75	-12.56	22.06	1.00E+00	9.63E+02	
449	LYS	pttt-mmmt	11.82	-0.05	23.69	5.25E-02	5.06E+01	
450	LYS	tptm-mmmt	0.89	-17.56	19.34	1.00E+00	9.63E+02	
451	LYS	tptp-mmmt	-5.10	-21.10	10.90	1.00E+00	9.63E+02	
452	LYS	tptt-mmmt	1.45	-10.91	13.81	1.00E+00	9.63E+02	
453	LYS	ttmm-mmmt	4.96	-13.54	23.46	1.00E+00	9.63E+02	
454	LYS	ttmt-mmmt	-0.16	-13.42	13.09	1.00E+00	9.63E+02	
455	LYS	ttpp-mmmt	-5.81	-23.16	11.54	1.00E+00	9.63E+02	
456	LYS	ttpt-mmmt	3.19	-9.49	15.88	1.00E+00	9.63E+02	
457	LYS	tttm-mmmt	13.26	1.45	25.06	9.09E-03	8.75E+00	
458	LYS	tttp-mmmt	17.00	5.12	28.89	3.95E-05	3.81E-02	*
459	LYS	tttt-mmmt	13.41	2.96	23.85	6.51E-04	6.27E-01	
460	LYS	mmtp-mmtm	7.78	-5.40	20.96	9.20E-01	8.86E+02	
461	LYS	mmtt-mmtm	-0.79	-10.24	8.65	1.00E+00	9.63E+02	
462	LYS	mptt-mmtm	-0.42	-23.50	22.66	1.00E+00	9.63E+02	
463	LYS	mtmm-mmtm	-5.34	-19.12	8.44	1.00E+00	9.63E+02	
464	LYS	mtmt-mmtm	-1.06	-11.60	9.48	1.00E+00	9.63E+02	
465	LYS	mtpp-mmtm	0.47	-13.52	14.45	1.00E+00	9.63E+02	
466	LYS	mtpt-mmtm	0.20	-10.31	10.71	1.00E+00	9.63E+02	
467	LYS	mttm-mmtm	7.44	-2.46	17.34	5.18E-01	4.99E+02	
468	LYS	mttp-mmtm	11.02	0.67	21.38	2.12E-02	2.04E+01	
469	LYS	mttt-mmtm	8.74	0.00	17.49	4.99E-02	4.81E+01	
470	LYS	off-mmtm	9.91	0.98	18.85	1.12E-02	1.08E+01	
471	LYS	ptmt-mmtm	5.50	-13.45	24.45	1.00E+00	9.63E+02	
472	LYS	ptpt-mmtm	-2.01	-21.67	17.65	1.00E+00	9.63E+02	
473	LYS	pttm-mmtm	-2.73	-19.38	13.92	1.00E+00	9.63E+02	
474	LYS	pttp-mmtm	-0.46	-16.94	16.02	1.00E+00	9.63E+02	
475	LYS	pttt-mmtm	6.61	-4.02	17.24	8.68E-01	8.35E+02	
476	LYS	tptm-mmtm	-4.32	-21.99	13.35	1.00E+00	9.63E+02	

477	LYS	tptp-mmtm	-10.31	-25.41	4.79	7.24E-01	6.97E+02	
478	LYS	tptt-mmtm	-3.76	-14.94	7.42	1.00E+00	9.63E+02	
479	LYS	ttmm-mmtm	-0.25	-17.98	17.48	1.00E+00	9.63E+02	
480	LYS	ttmt-mmtm	-5.37	-17.53	6.78	9.98E-01	9.61E+02	
481	LYS	ttpp-mmtm	-11.02	-27.54	5.50	7.66E-01	7.37E+02	
482	LYS	ttpt-mmtm	-2.02	-13.55	9.51	1.00E+00	9.63E+02	
483	LYS	tttm-mmtm	8.05	-2.51	18.60	4.85E-01	4.67E+02	
484	LYS	tttp-mmtm	11.79	1.14	22.44	1.15E-02	1.11E+01	
485	LYS	tttt-mmtm	8.19	-0.82	17.21	1.42E-01	1.37E+02	
486	LYS	mmtt-mmtp	-8.57	-19.70	2.56	4.62E-01	4.45E+02	
487	LYS	mptt-mmtp	-8.20	-32.02	15.62	1.00E+00	9.63E+02	
488	LYS	mtmm-mmtp	-13.12	-28.10	1.87	1.99E-01	1.92E+02	
489	LYS	mtmt-mmtp	-8.84	-20.91	3.24	5.78E-01	5.57E+02	
490	LYS	mtpp-mmtp	-7.31	-22.48	7.86	9.93E-01	9.57E+02	
491	LYS	mtpt-mmtp	-7.58	-19.63	4.47	8.53E-01	8.22E+02	
492	LYS	mttm-mmtp	-0.34	-11.85	11.18	1.00E+00	9.63E+02	
493	LYS	mttp-mmtp	3.25	-8.66	15.16	1.00E+00	9.63E+02	
494	LYS	mttt-mmtp	0.97	-9.57	11.51	1.00E+00	9.63E+02	
495	LYS	off-mmtp	2.13	-8.57	12.84	1.00E+00	9.63E+02	
496	LYS	ptmt-mmtp	-2.28	-22.12	17.57	1.00E+00	9.63E+02	
497	LYS	ptpt-mmtp	-9.79	-30.31	10.73	9.94E-01	9.58E+02	
498	LYS	pttm-mmtp	-10.51	-28.17	7.15	9.13E-01	8.79E+02	
499	LYS	pttp-mmtp	-8.24	-25.74	9.26	9.95E-01	9.59E+02	
500	LYS	pttt-mmtp	-1.17	-13.32	10.98	1.00E+00	9.63E+02	
501	LYS	tptm-mmtp	-12.10	-30.73	6.53	8.09E-01	7.79E+02	
502	LYS	tptp-mmtp	-18.09	-34.29	-1.88	1.01E-02	9.76E+00	
503	LYS	tptt-mmtp	-11.54	-24.17	1.09	1.36E-01	1.31E+02	
504	LYS	ttmm-mmtp	-8.03	-26.71	10.65	9.99E-01	9.62E+02	
505	LYS	ttmt-mmtp	-13.15	-26.66	0.36	6.91E-02	6.65E+01	
506	LYS	ttpp-mmtp	-18.80	-36.34	-1.26	1.93E-02	1.85E+01	
507	LYS	ttpt-mmtp	-9.79	-22.74	3.15	5.03E-01	4.84E+02	
508	LYS	tttm-mmtp	0.27	-11.82	12.36	1.00E+00	9.63E+02	
509	LYS	tttp-mmtp	4.02	-8.15	16.19	1.00E+00	9.63E+02	
510	LYS	tttt-mmtp	0.42	-10.35	11.18	1.00E+00	9.63E+02	
511	LYS	mptt-mmtt	0.37	-21.60	22.35	1.00E+00	9.63E+02	
512	LYS	mtmm-mmtt	-4.55	-16.38	7.29	1.00E+00	9.63E+02	
513	LYS	mtmt-mmtt	-0.27	-8.09	7.56	1.00E+00	9.63E+02	
514	LYS	mtpp-mmtt	1.26	-10.81	13.33	1.00E+00	9.63E+02	
515	LYS	mtpt-mmtt	0.99	-6.79	8.77	1.00E+00	9.63E+02	
516	LYS	mttm-mmtt	8.23	1.30	15.17	3.35E-03	3.23E+00	
517	LYS	mttp-mmtt	11.81	4.25	19.38	2.46E-06	2.37E-03	*
518	LYS	mttt-mmtt	9.54	4.38	14.69	0.00E+00	0.00E+00	*
519	LYS	off-mmtt	10.70	5.23	16.18	0.00E+00	0.00E+00	*
520	LYS	ptmt-mmtt	6.29	-11.29	23.88	1.00E+00	9.63E+02	
521	LYS	ptpt-mmtt	-1.22	-19.57	17.13	1.00E+00	9.63E+02	
522	LYS	pttm-mmtt	-1.94	-17.02	13.14	1.00E+00	9.63E+02	
523	LYS	pttp-mmtt	0.33	-14.56	15.22	1.00E+00	9.63E+02	
524	LYS	pttt-mmtt	7.40	-0.54	15.34	1.11E-01	1.07E+02	

525	LYS	tptm-mmtt	-3.53	-19.73	12.67	1.00E+00	9.63E+02	
526	LYS	tptp-mmtt	-9.52	-22.86	3.83	6.36E-01	6.12E+02	
527	LYS	tptt-mmtt	-2.97	-11.63	5.69	1.00E+00	9.63E+02	
528	LYS	ttmm-mmtt	0.54	-15.72	16.80	1.00E+00	9.63E+02	
529	LYS	ttmt-mmtt	-4.58	-14.47	5.31	9.96E-01	9.60E+02	
530	LYS	ttpp-mmtt	-10.23	-25.16	4.71	7.18E-01	6.91E+02	
531	LYS	ttpt-mmtt	-1.22	-10.34	7.89	1.00E+00	9.63E+02	
532	LYS	tttm-mmtt	8.84	0.99	16.68	8.61E-03	8.29E+00	
533	LYS	tttp-mmtt	12.59	4.61	20.56	1.63E-06	1.57E-03	*
534	LYS	tttt-mmtt	8.99	3.38	14.59	8.95E-07	8.62E-04	*
535	LYS	mtmm-mptt	-4.92	-29.08	19.24	1.00E+00	9.63E+02	
536	LYS	mtmt-mptt	-0.64	-23.11	21.83	1.00E+00	9.63E+02	
537	LYS	mtpp-mptt	0.89	-23.39	25.16	1.00E+00	9.63E+02	
538	LYS	mtpt-mptt	0.62	-21.84	23.07	1.00E+00	9.63E+02	
539	LYS	mttm-mptt	7.86	-14.31	30.04	1.00E+00	9.63E+02	
540	LYS	mttp-mptt	11.44	-10.94	33.82	9.85E-01	9.49E+02	
541	LYS	mttt-mptt	9.16	-12.52	30.85	9.99E-01	9.62E+02	
542	LYS	off-mptt	10.33	-11.43	32.09	9.95E-01	9.58E+02	
543	LYS	ptmt-mptt	5.92	-21.52	33.36	1.00E+00	9.63E+02	
544	LYS	ptpt-mptt	-1.59	-29.53	26.34	1.00E+00	9.63E+02	
545	LYS	pttm-mptt	-2.31	-28.21	23.59	1.00E+00	9.63E+02	
546	LYS	pttp-mptt	-0.04	-25.84	25.75	1.00E+00	9.63E+02	
547	LYS	pttt-mptt	7.03	-15.48	29.54	1.00E+00	9.63E+02	
548	LYS	tptm-mptt	-3.90	-30.47	22.67	1.00E+00	9.63E+02	
549	LYS	tptp-mptt	-9.89	-34.82	15.05	1.00E+00	9.63E+02	
550	LYS	tptt-mptt	-3.34	-26.11	19.43	1.00E+00	9.63E+02	
551	LYS	ttmm-mptt	0.17	-26.44	26.78	1.00E+00	9.63E+02	
552	LYS	ttmt-mptt	-4.95	-28.22	18.32	1.00E+00	9.63E+02	
553	LYS	ttpp-mptt	-10.60	-36.42	15.22	9.99E-01	9.63E+02	
554	LYS	ttpt-mptt	-1.60	-24.55	21.35	1.00E+00	9.63E+02	
555	LYS	tttm-mptt	8.47	-14.01	30.94	1.00E+00	9.63E+02	
556	LYS	tttp-mptt	12.21	-10.31	34.73	9.69E-01	9.33E+02	
557	LYS	tttt-mptt	8.61	-13.18	30.41	1.00E+00	9.63E+02	
558	LYS	mtmt-mtmm	4.28	-8.45	17.01	1.00E+00	9.63E+02	
559	LYS	mtpp-mtmm	5.80	-9.89	21.50	1.00E+00	9.63E+02	
560	LYS	mtpt-mtmm	5.54	-7.17	18.24	9.99E-01	9.62E+02	
561	LYS	mttm-mtmm	12.78	0.58	24.98	2.70E-02	2.60E+01	
562	LYS	mttp-mtmm	16.36	3.79	28.93	4.68E-04	4.51E-01	
563	LYS	mttt-mtmm	14.08	2.80	25.37	1.20E-03	1.16E+00	
564	LYS	off-mtmm	15.25	3.82	26.69	2.58E-04	2.48E-01	
565	LYS	ptmt-mtmm	10.84	-9.41	31.09	9.73E-01	9.37E+02	
566	LYS	ptpt-mtmm	3.33	-17.59	24.24	1.00E+00	9.63E+02	
567	LYS	pttm-mtmm	2.61	-15.51	20.72	1.00E+00	9.63E+02	
568	LYS	pttp-mtmm	4.88	-13.08	22.84	1.00E+00	9.63E+02	
569	LYS	pttt-mtmm	11.95	-0.85	24.75	1.09E-01	1.05E+02	
570	LYS	tptm-mtmm	1.02	-18.04	20.08	1.00E+00	9.63E+02	
571	LYS	tptp-mtmm	-4.97	-21.67	11.73	1.00E+00	9.63E+02	
572	LYS	tptt-mtmm	1.58	-11.68	14.84	1.00E+00	9.63E+02	

573	LYS	ttmm-mtmm	5.09	-14.03	24.20	1.00E+00	9.63E+02	
574	LYS	ttmt-mtmm	-0.03	-14.13	14.06	1.00E+00	9.63E+02	
575	LYS	ttpp-mtmm	-5.68	-23.68	12.32	1.00E+00	9.63E+02	
576	LYS	ttpt-mtmm	3.32	-10.24	16.88	1.00E+00	9.63E+02	
577	LYS	tttm-mtmm	13.39	0.64	26.13	2.58E-02	2.48E+01	
578	LYS	tttp-mtmm	17.13	4.31	29.95	2.45E-04	2.36E-01	
579	LYS	tttt-mtmm	13.53	2.04	25.03	3.92E-03	3.78E+00	
580	LYS	mtpp-mtmt	1.53	-11.42	14.47	1.00E+00	9.63E+02	
581	LYS	mtpt-mtmt	1.26	-7.83	10.34	1.00E+00	9.63E+02	
582	LYS	mttm-mtmt	8.50	0.13	16.87	4.10E-02	3.94E+01	
583	LYS	mttp-mtmt	12.08	3.18	20.98	1.65E-04	1.59E-01	
584	LYS	mttt-mtmt	9.80	2.84	16.77	6.18E-05	5.95E-02	
585	LYS	off-mtmt	10.97	3.77	18.18	5.69E-06	5.48E-03	*
586	LYS	ptmt-mtmt	6.56	-11.64	24.76	1.00E+00	9.63E+02	
587	LYS	ptpt-mtmt	-0.95	-19.89	17.98	1.00E+00	9.63E+02	
588	LYS	pttm-mtmt	-1.67	-17.46	14.12	1.00E+00	9.63E+02	
589	LYS	pttp-mtmt	0.60	-15.01	16.21	1.00E+00	9.63E+02	
590	LYS	pttt-mtmt	7.67	-1.55	16.89	2.92E-01	2.81E+02	
591	LYS	tptm-mtmt	-3.26	-20.13	13.60	1.00E+00	9.63E+02	
592	LYS	tptp-mtmt	-9.25	-23.39	4.90	7.99E-01	7.69E+02	
593	LYS	tptt-mtmt	-2.70	-12.55	7.15	1.00E+00	9.63E+02	
594	LYS	ttmm-mtmt	0.81	-16.12	17.73	1.00E+00	9.63E+02	
595	LYS	ttmt-mtmt	-4.31	-15.26	6.63	1.00E+00	9.63E+02	
596	LYS	ttpp-mtmt	-9.96	-25.61	5.69	8.39E-01	8.08E+02	
597	LYS	ttpt-mtmt	-0.96	-11.20	9.29	1.00E+00	9.63E+02	
598	LYS	tttm-mtmt	9.11	-0.03	18.24	5.21E-02	5.01E+01	
599	LYS	tttp-mtmt	12.85	3.61	22.10	8.75E-05	8.43E-02	
600	LYS	tttt-mtmt	9.25	1.95	16.56	8.58E-04	8.26E-01	
601	LYS	mtpt-mtpp	-0.27	-13.19	12.65	1.00E+00	9.63E+02	
602	LYS	mttm-mtpp	6.98	-5.45	19.40	9.53E-01	9.18E+02	
603	LYS	mttp-mtpp	10.56	-2.23	23.35	3.09E-01	2.97E+02	
604	LYS	mttt-mtpp	8.28	-3.25	19.81	6.20E-01	5.97E+02	
605	LYS	off-mtpp	9.45	-2.23	21.12	3.51E-01	3.38E+02	
606	LYS	ptmt-mtpp	5.03	-15.35	25.42	1.00E+00	9.63E+02	
607	LYS	ptpt-mtpp	-2.48	-23.53	18.57	1.00E+00	9.63E+02	
608	LYS	pttm-mtpp	-3.20	-21.46	15.07	1.00E+00	9.63E+02	
609	LYS	pttp-mtpp	-0.93	-19.04	17.19	1.00E+00	9.63E+02	
610	LYS	pttt-mtpp	6.14	-6.87	19.16	9.95E-01	9.58E+02	
611	LYS	tptm-mtpp	-4.79	-23.99	14.42	1.00E+00	9.63E+02	
612	LYS	tptp-mtpp	-10.77	-27.64	6.09	8.33E-01	8.02E+02	
613	LYS	tptt-mtpp	-4.23	-17.69	9.24	1.00E+00	9.63E+02	
614	LYS	ttmm-mtpp	-0.72	-19.97	18.54	1.00E+00	9.63E+02	
615	LYS	ttmt-mtpp	-5.84	-20.13	8.45	1.00E+00	9.63E+02	
616	LYS	ttpp-mtpp	-11.49	-29.64	6.67	8.46E-01	8.15E+02	
617	LYS	ttpt-mtpp	-2.48	-16.24	11.28	1.00E+00	9.63E+02	
618	LYS	tttm-mtpp	7.58	-5.38	20.54	9.27E-01	8.92E+02	
619	LYS	tttp-mtpp	11.33	-1.71	24.36	2.11E-01	2.03E+02	
620	LYS	tttt-mtpp	7.73	-4.01	19.46	7.87E-01	7.58E+02	

621	LYS	mttm-mtpt	7.24	-1.09	15.57	2.10E-01	2.02E+02	
622	LYS	mttp-mtpt	10.83	1.96	19.69	1.89E-03	1.82E+00	
623	LYS	mttt-mtpt	8.55	1.63	15.46	1.48E-03	1.42E+00	
624	LYS	off-mtpt	9.72	2.56	16.87	1.65E-04	1.59E-01	
625	LYS	ptmt-mtpt	5.30	-12.88	23.48	1.00E+00	9.63E+02	
626	LYS	ptpt-mtpt	-2.21	-21.13	16.71	1.00E+00	9.63E+02	
627	LYS	pttm-mtpt	-2.93	-18.69	12.84	1.00E+00	9.63E+02	
628	LYS	pttp-mtpt	-0.66	-16.25	14.93	1.00E+00	9.63E+02	
629	LYS	pttt-mtpt	6.41	-2.77	15.60	6.79E-01	6.53E+02	
630	LYS	tptm-mtpt	-4.52	-21.36	12.33	1.00E+00	9.63E+02	
631	LYS	tptp-mtpt	-10.51	-24.63	3.62	5.41E-01	5.21E+02	
632	LYS	tptt-mtpt	-3.96	-13.77	5.85	1.00E+00	9.63E+02	
633	LYS	ttmm-mtpt	-0.45	-17.35	16.45	1.00E+00	9.63E+02	
634	LYS	ttmt-mtpt	-5.57	-16.48	5.35	9.86E-01	9.49E+02	
635	LYS	ttpp-mtpt	-11.22	-26.85	4.42	6.22E-01	5.99E+02	
636	LYS	ttpt-mtpt	-2.21	-12.43	8.00	1.00E+00	9.63E+02	
637	LYS	tttm-mtpt	7.85	-1.25	16.95	2.24E-01	2.15E+02	
638	LYS	tttp-mtpt	11.60	2.39	20.81	9.89E-04	9.52E-01	
639	LYS	tttt-mtpt	8.00	0.74	15.25	1.24E-02	1.19E+01	
640	LYS	mttp-mttm	3.58	-4.55	11.71	9.98E-01	9.61E+02	
641	LYS	mttt-mttm	1.30	-4.64	7.25	1.00E+00	9.63E+02	
642	LYS	off-mttm	2.47	-3.76	8.70	1.00E+00	9.63E+02	
643	LYS	ptmt-mttm	-1.94	-19.77	15.89	1.00E+00	9.63E+02	
644	LYS	ptpt-mttm	-9.45	-28.04	9.13	9.86E-01	9.50E+02	
645	LYS	pttm-mttm	-10.17	-25.54	5.19	7.79E-01	7.50E+02	
646	LYS	pttp-mttm	-7.90	-23.09	7.28	9.81E-01	9.45E+02	
647	LYS	pttt-mttm	-0.83	-9.31	7.65	1.00E+00	9.63E+02	
648	LYS	tptm-mttm	-11.76	-28.23	4.71	6.32E-01	6.09E+02	
649	LYS	tptp-mttm	-17.75	-31.42	-4.08	4.96E-04	4.77E-01	
650	LYS	tptt-mttm	-11.20	-20.36	-2.05	1.83E-03	1.76E+00	
651	LYS	ttmm-mttm	-7.69	-24.22	8.84	9.96E-01	9.59E+02	
652	LYS	ttmt-mttm	-12.81	-23.14	-2.48	1.37E-03	1.32E+00	
653	LYS	ttpp-mttm	-18.46	-33.69	-3.23	2.21E-03	2.13E+00	
654	LYS	ttpt-mttm	-9.46	-19.04	0.13	5.90E-02	5.68E+01	
655	LYS	tttm-mttm	0.61	-7.78	8.99	1.00E+00	9.63E+02	
656	LYS	tttp-mttm	4.35	-4.15	12.86	9.85E-01	9.49E+02	
657	LYS	tttt-mttm	0.75	-5.59	7.09	1.00E+00	9.63E+02	
658	LYS	mttt-mttp	-2.28	-8.95	4.40	1.00E+00	9.63E+02	
659	LYS	off-mttp	-1.11	-8.04	5.81	1.00E+00	9.63E+02	
660	LYS	ptmt-mttp	-5.52	-23.61	12.57	1.00E+00	9.63E+02	
661	LYS	ptpt-mttp	-13.04	-31.87	5.79	6.97E-01	6.71E+02	
662	LYS	pttm-mttp	-13.75	-29.42	1.91	1.93E-01	1.86E+02	
663	LYS	pttp-mttp	-11.48	-26.97	4.00	5.48E-01	5.28E+02	
664	LYS	pttt-mttp	-4.41	-13.41	4.59	9.92E-01	9.55E+02	
665	LYS	tptm-mttp	-15.34	-32.09	1.40	1.32E-01	1.27E+02	
666	LYS	tptp-mttp	-21.33	-35.34	-7.33	5.67E-06	5.46E-03	*
667	LYS	tptt-mttp	-14.78	-24.43	-5.14	4.58E-06	4.41E-03	*
668	LYS	ttmm-mttp	-11.28	-28.08	5.53	7.55E-01	7.28E+02	

669	LYS	ttmt-mttp	-16.40	-27.16	-5.63	5.66E-06	5.45E-03	*
670	LYS	ttpp-mttp	-22.04	-37.57	-6.52	4.90E-05	4.72E-02	*
671	LYS	ttpt-mttp	-13.04	-23.09	-2.99	5.04E-04	4.86E-01	
672	LYS	tttm-mttp	-2.98	-11.89	5.94	1.00E+00	9.63E+02	
673	LYS	tttp-mttp	0.77	-8.26	9.80	1.00E+00	9.63E+02	
674	LYS	tttt-mttp	-2.83	-9.85	4.20	1.00E+00	9.63E+02	
675	LYS	off-mttt	1.17	-2.98	5.32	1.00E+00	9.63E+02	
676	LYS	ptmt-mttt	-3.24	-20.46	13.97	1.00E+00	9.63E+02	
677	LYS	ptpt-mttt	-10.76	-28.76	7.24	9.09E-01	8.75E+02	
678	LYS	pttm-mttt	-11.48	-26.12	3.17	4.22E-01	4.07E+02	
679	LYS	pttp-mttt	-9.21	-23.66	5.25	8.38E-01	8.07E+02	
680	LYS	pttt-mttt	-2.13	-9.23	4.96	1.00E+00	9.63E+02	
681	LYS	tptm-mttt	-13.07	-28.87	2.74	3.05E-01	2.94E+02	
682	LYS	tptp-mttt	-19.05	-31.91	-6.19	1.38E-05	1.33E-02	*
683	LYS	tptt-mttt	-12.50	-20.40	-4.61	1.42E-06	1.37E-03	*
684	LYS	ttmm-mttt	-9.00	-24.86	6.87	9.48E-01	9.12E+02	
685	LYS	ttmt-mttt	-14.12	-23.34	-4.89	4.87E-06	4.69E-03	*
686	LYS	ttpp-mttt	-19.76	-34.27	-5.26	1.49E-04	1.44E-01	
687	LYS	ttpt-mttt	-10.76	-19.15	-2.38	6.46E-04	6.23E-01	
688	LYS	tttm-mttt	-0.70	-7.68	6.29	1.00E+00	9.63E+02	
689	LYS	tttp-mttt	3.05	-4.08	10.18	9.99E-01	9.62E+02	
690	LYS	tttt-mttt	-0.55	-4.87	3.77	1.00E+00	9.63E+02	
691	LYS	ptmt-off	-4.41	-21.73	12.90	1.00E+00	9.63E+02	
692	LYS	ptpt-off	-11.93	-30.02	6.17	7.85E-01	7.56E+02	
693	LYS	pttm-off	-12.64	-27.41	2.12	2.37E-01	2.28E+02	
694	LYS	pttp-off	-10.37	-24.95	4.20	6.40E-01	6.16E+02	
695	LYS	pttt-off	-3.30	-10.63	4.03	9.98E-01	9.61E+02	
696	LYS	tptm-off	-14.23	-30.14	1.68	1.65E-01	1.59E+02	
697	LYS	tptp-off	-20.22	-33.21	-7.23	2.72E-06	2.62E-03	*
698	LYS	tptt-off	-13.67	-21.77	-5.57	7.23E-08	6.96E-05	*
699	LYS	ttmm-off	-10.16	-26.14	5.81	8.38E-01	8.07E+02	
700	LYS	ttmt-off	-15.28	-24.69	-5.88	5.38E-07	5.18E-04	*
701	LYS	ttpp-off	-20.93	-35.55	-6.31	3.85E-05	3.70E-02	*
702	LYS	ttpt-off	-11.93	-20.51	-3.34	8.91E-05	8.58E-02	
703	LYS	tttm-off	-1.87	-9.09	5.36	1.00E+00	9.63E+02	
704	LYS	tttp-off	1.88	-5.48	9.24	1.00E+00	9.63E+02	
705	LYS	tttt-off	-1.72	-6.41	2.98	1.00E+00	9.63E+02	
706	LYS	ptpt-ptmt	-7.51	-32.14	17.11	1.00E+00	9.63E+02	
707	LYS	pttm-ptmt	-8.23	-30.53	14.07	1.00E+00	9.63E+02	
708	LYS	pttp-ptmt	-5.96	-28.13	16.21	1.00E+00	9.63E+02	
709	LYS	pttt-ptmt	1.11	-17.14	19.36	1.00E+00	9.63E+02	
710	LYS	tptm-ptmt	-9.82	-32.89	13.25	9.99E-01	9.62E+02	
711	LYS	tptp-ptmt	-15.81	-36.97	5.36	5.32E-01	5.12E+02	
712	LYS	tptt-ptmt	-9.26	-27.83	9.31	9.89E-01	9.53E+02	
713	LYS	ttmm-ptmt	-5.75	-28.87	17.36	1.00E+00	9.63E+02	
714	LYS	ttmt-ptmt	-10.87	-30.05	8.30	9.48E-01	9.13E+02	
715	LYS	ttpp-ptmt	-16.52	-38.72	5.68	5.41E-01	5.21E+02	
716	LYS	ttpt-ptmt	-7.52	-26.30	11.27	1.00E+00	9.63E+02	

717	LYS	tttm-ptmt	2.55	-15.66	20.75	1.00E+00	9.63E+02
718	LYS	tttp-ptmt	6.29	-11.97	24.55	1.00E+00	9.63E+02
719	LYS	tttt-ptmt	2.69	-14.66	20.05	1.00E+00	9.63E+02
720	LYS	pttm-ptpt	-0.72	-23.62	22.19	1.00E+00	9.63E+02
721	LYS	pttp-ptpt	1.55	-21.23	24.34	1.00E+00	9.63E+02
722	LYS	pttt-ptpt	8.62	-10.36	27.61	9.97E-01	9.60E+02
723	LYS	tptm-ptpt	-2.31	-25.97	21.35	1.00E+00	9.63E+02
724	LYS	tptp-ptpt	-8.29	-30.10	13.51	1.00E+00	9.63E+02
725	LYS	tptt-ptpt	-1.75	-21.04	17.55	1.00E+00	9.63E+02
726	LYS	ttmm-ptpt	1.76	-21.94	25.46	1.00E+00	9.63E+02
727	LYS	ttmt-ptpt	-3.36	-23.24	16.52	1.00E+00	9.63E+02
728	LYS	ttpp-ptpt	-9.01	-31.82	13.81	1.00E+00	9.63E+02
729	LYS	ttpt-ptpt	0.00	-19.50	19.50	1.00E+00	9.63E+02
730	LYS	tttm-ptpt	10.06	-8.88	29.00	9.76E-01	9.40E+02
731	LYS	tttp-ptpt	13.81	-5.19	32.80	5.94E-01	5.72E+02
732	LYS	tttt-ptpt	10.21	-7.92	28.34	9.52E-01	9.16E+02
733	LYS	pttp-pttm	2.27	-17.97	22.51	1.00E+00	9.63E+02
734	LYS	pttt-pttm	9.34	-6.50	25.19	9.21E-01	8.87E+02
735	LYS	tptm-pttm	-1.59	-22.81	19.64	1.00E+00	9.63E+02
736	LYS	tptp-pttm	-7.58	-26.71	11.56	1.00E+00	9.63E+02
737	LYS	tptt-pttm	-1.03	-17.25	15.19	1.00E+00	9.63E+02
738	LYS	ttmm-pttm	2.48	-18.79	23.75	1.00E+00	9.63E+02
739	LYS	ttmt-pttm	-2.64	-19.55	14.27	1.00E+00	9.63E+02
740	LYS	ttpp-pttm	-8.29	-28.56	11.99	1.00E+00	9.63E+02
741	LYS	ttpt-pttm	0.72	-15.75	17.18	1.00E+00	9.63E+02
742	LYS	tttm-pttm	10.78	-5.02	26.58	7.25E-01	6.98E+02
743	LYS	tttp-pttm	14.53	-1.34	30.39	1.32E-01	1.28E+02
744	LYS	tttt-pttm	10.93	-3.89	25.74	5.60E-01	5.40E+02
745	LYS	pttt-pttp	7.07	-8.60	22.74	9.98E-01	9.61E+02
746	LYS	tptm-pttp	-3.86	-24.95	17.23	1.00E+00	9.63E+02
747	LYS	tptp-pttp	-9.85	-28.84	9.14	9.82E-01	9.46E+02
748	LYS	tptt-pttp	-3.30	-19.34	12.75	1.00E+00	9.63E+02
749	LYS	ttmm-pttp	0.21	-20.93	21.35	1.00E+00	9.63E+02
750	LYS	ttmt-pttp	-4.91	-21.65	11.83	1.00E+00	9.63E+02
751	LYS	ttpp-pttp	-10.56	-30.70	9.58	9.79E-01	9.43E+02
752	LYS	ttpt-pttp	-1.56	-17.85	14.74	1.00E+00	9.63E+02
753	LYS	tttm-pttp	8.51	-7.11	24.13	9.67E-01	9.31E+02
754	LYS	tttp-pttp	12.25	-3.43	27.94	4.29E-01	4.13E+02
755	LYS	tttt-pttp	8.65	-5.97	23.28	9.17E-01	8.84E+02
756	LYS	tptm-pttt	-10.93	-27.85	5.99	8.17E-01	7.87E+02
757	LYS	tptp-pttt	-16.92	-31.13	-2.71	3.15E-03	3.03E+00
758	LYS	tptt-pttt	-10.37	-20.31	-0.43	2.83E-02	2.73E+01
759	LYS	ttmm-pttt	-6.86	-23.84	10.11	1.00E+00	9.63E+02
760	LYS	ttmt-pttt	-11.98	-23.01	-0.96	1.56E-02	1.50E+01
761	LYS	ttpp-pttt	-17.63	-33.34	-1.92	9.23E-03	8.89E+00
762	LYS	ttpt-pttt	-8.63	-18.96	1.71	2.86E-01	2.75E+02
763	LYS	tttm-pttt	1.44	-7.80	10.67	1.00E+00	9.63E+02
764	LYS	tttp-pttt	5.18	-4.16	14.53	9.59E-01	9.24E+02

765	LYS	tttt-pttt	1.58	-5.84	9.01	1.00E+00	9.63E+02	
766	LYS	tptp-tptm	-5.99	-26.02	14.04	1.00E+00	9.63E+02	
767	LYS	tptt-tptm	0.56	-16.71	17.83	1.00E+00	9.63E+02	
768	LYS	ttmm-tptm	4.07	-18.02	26.15	1.00E+00	9.63E+02	
769	LYS	ttmt-tptm	-1.05	-18.97	16.87	1.00E+00	9.63E+02	
770	LYS	ttpp-tptm	-6.70	-27.83	14.43	1.00E+00	9.63E+02	
771	LYS	ttpt-tptm	2.30	-15.20	19.80	1.00E+00	9.63E+02	
772	LYS	tttm-tptm	12.37	-4.51	29.24	5.75E-01	5.54E+02	
773	LYS	tttp-tptm	16.11	-0.82	33.05	8.93E-02	8.60E+01	
774	LYS	tttt-tptm	12.51	-3.44	28.47	4.20E-01	4.04E+02	
775	LYS	tptt-tptp	6.55	-8.07	21.17	9.98E-01	9.61E+02	
776	LYS	ttmm-tptp	10.06	-10.03	30.14	9.89E-01	9.52E+02	
777	LYS	ttmt-tptp	4.94	-10.45	20.32	1.00E+00	9.63E+02	
778	LYS	ttpp-tptp	-0.71	-19.74	18.31	1.00E+00	9.63E+02	
779	LYS	ttpt-tptp	8.29	-6.60	23.19	9.57E-01	9.22E+02	
780	LYS	tttm-tptp	18.36	4.20	32.51	5.08E-04	4.90E-01	
781	LYS	tttp-tptp	22.10	7.88	36.33	2.89E-06	2.79E-03	*
782	LYS	tttt-tptp	18.50	5.46	31.55	5.04E-05	4.85E-02	*
783	LYS	ttmm-tptt	3.51	-13.82	20.83	1.00E+00	9.63E+02	
784	LYS	ttmt-tptt	-1.61	-13.17	9.94	1.00E+00	9.63E+02	
785	LYS	ttpp-tptt	-7.26	-23.35	8.83	9.98E-01	9.61E+02	
786	LYS	ttpt-tptt	1.74	-9.15	12.64	1.00E+00	9.63E+02	
787	LYS	tttm-tptt	11.81	1.95	21.67	2.83E-03	2.72E+00	
788	LYS	tttp-tptt	15.55	5.59	25.52	2.44E-06	2.35E-03	*
789	LYS	tttt-tptt	11.95	3.76	20.14	2.18E-05	2.10E-02	*
790	LYS	ttmt-ttmm	-5.12	-23.09	12.85	1.00E+00	9.63E+02	
791	LYS	ttpp-ttmm	-10.77	-31.94	10.41	9.86E-01	9.50E+02	
792	LYS	ttpt-ttmm	-1.76	-19.32	15.79	1.00E+00	9.63E+02	
793	LYS	tttm-ttmm	8.30	-8.63	25.23	9.92E-01	9.55E+02	
794	LYS	tttp-ttmm	12.05	-4.94	29.04	6.48E-01	6.24E+02	
795	LYS	tttt-ttmm	8.45	-7.57	24.46	9.78E-01	9.42E+02	
796	LYS	ttpp-ttmt	-5.65	-22.43	11.14	1.00E+00	9.63E+02	
797	LYS	ttpt-ttmt	3.36	-8.54	15.26	1.00E+00	9.63E+02	
798	LYS	tttm-ttmt	13.42	2.46	24.38	1.80E-03	1.73E+00	
799	LYS	tttp-ttmt	17.17	6.12	28.22	2.90E-06	2.80E-03	*
800	LYS	tttt-ttmt	13.57	4.08	23.05	3.93E-05	3.79E-02	*
801	LYS	ttpt-ttpp	9.00	-7.33	25.34	9.62E-01	9.26E+02	
802	LYS	tttm-ttpp	19.07	3.40	34.73	2.03E-03	1.95E+00	
803	LYS	tttp-ttpp	22.81	7.09	38.54	2.62E-05	2.52E-02	*
804	LYS	tttt-ttpp	19.21	4.55	33.88	4.01E-04	3.86E-01	
805	LYS	tttm-ttpt	10.06	-0.20	20.32	6.34E-02	6.11E+01	
806	LYS	tttp-ttpt	13.81	3.45	24.17	2.60E-04	2.50E-01	
807	LYS	tttt-ttpt	10.21	1.54	18.88	3.87E-03	3.72E+00	
808	LYS	tttp-tttm	3.75	-5.51	13.01	1.00E+00	9.63E+02	
809	LYS	tttt-tttm	0.15	-7.17	7.47	1.00E+00	9.63E+02	
810	LYS	tttt-tttp	-3.60	-11.06	3.86	9.93E-01	9.57E+02	
811	MET	mmp-mmm	-1.73	-10.14	6.68	1.00E+00	9.63E+02	
812	MET	mmt-mmm	-2.55	-11.68	6.57	1.00E+00	9.63E+02	

813	MET	mtm-mmm	-4.54	-10.35	1.27	3.26E-01	3.14E+02
814	MET	mtp-mmm	-2.91	-8.05	2.24	8.30E-01	8.00E+02
815	MET	mtt-mmm	-5.37	-11.81	1.08	2.28E-01	2.19E+02
816	MET	off-mmm	-0.25	-5.79	5.29	1.00E+00	9.63E+02
817	MET	ptm-mmm	-5.77	-16.06	4.51	8.36E-01	8.05E+02
818	MET	ptp-mmm	2.27	-8.45	13.00	1.00E+00	9.63E+02
819	MET	tpp-mmm	-6.62	-13.76	0.52	1.03E-01	9.96E+01
820	MET	tpt-mmm	-12.33	-26.74	2.09	1.91E-01	1.84E+02
821	MET	ttm-mmm	-0.07	-7.17	7.04	1.00E+00	9.63E+02
822	MET	ttp-mmm	-5.22	-11.94	1.50	3.36E-01	3.24E+02
823	MET	ttt-mmm	-9.50	-18.90	-0.09	4.52E-02	4.35E+01
824	MET	mmt-mmp	-0.82	-12.17	10.53	1.00E+00	9.63E+02
825	MET	mtm-mmp	-2.81	-11.72	6.09	9.99E-01	9.62E+02
826	MET	mtp-mmp	-1.18	-9.67	7.31	1.00E+00	9.63E+02
827	MET	mtt-mmp	-3.64	-12.97	5.69	9.90E-01	9.54E+02
828	MET	off-mmp	1.48	-7.26	10.21	1.00E+00	9.63E+02
829	MET	ptm-mmp	-4.04	-16.34	8.26	9.98E-01	9.61E+02
830	MET	ptp-mmp	4.00	-8.67	16.68	9.99E-01	9.62E+02
831	MET	tpp-mmp	-4.89	-14.71	4.94	9.27E-01	8.92E+02
832	MET	tpt-mmp	-10.60	-26.52	5.32	6.04E-01	5.82E+02
833	MET	ttm-mmp	1.66	-8.14	11.46	1.00E+00	9.63E+02
834	MET	ttp-mmp	-3.49	-13.01	6.03	9.95E-01	9.58E+02
835	MET	ttt-mmp	-7.77	-19.35	3.81	5.92E-01	5.70E+02
836	MET	mtm-mmt	-1.99	-11.57	7.59	1.00E+00	9.63E+02
837	MET	mtp-mmt	-0.35	-9.55	8.84	1.00E+00	9.63E+02
838	MET	mtt-mmt	-2.82	-12.80	7.17	1.00E+00	9.63E+02
839	MET	off-mmt	2.30	-7.12	11.72	1.00E+00	9.63E+02
840	MET	ptm-mmt	-3.22	-16.02	9.58	1.00E+00	9.63E+02
841	MET	ptp-mmt	4.83	-8.33	17.99	9.95E-01	9.58E+02
842	MET	tpp-mmt	-4.06	-14.51	6.38	9.90E-01	9.54E+02
843	MET	tpt-mmt	-9.77	-26.08	6.53	7.61E-01	7.33E+02
844	MET	ttm-mmt	2.48	-7.93	12.90	1.00E+00	9.63E+02
845	MET	ttp-mmt	-2.67	-12.83	7.49	1.00E+00	9.63E+02
846	MET	ttt-mmt	-6.94	-19.05	5.16	8.13E-01	7.83E+02
847	MET	mtp-mtm	1.64	-4.29	7.56	1.00E+00	9.63E+02
848	MET	mtt-mtm	-0.83	-7.91	6.26	1.00E+00	9.63E+02
849	MET	off-mtm	4.29	-1.98	10.56	5.58E-01	5.38E+02
850	MET	ptm-mtm	-1.23	-11.92	9.46	1.00E+00	9.63E+02
851	MET	ptp-mtm	6.82	-4.30	17.94	7.31E-01	7.04E+02
852	MET	tpp-mtm	-2.07	-9.79	5.64	1.00E+00	9.63E+02
853	MET	tpt-mtm	-7.78	-22.49	6.93	8.87E-01	8.55E+02
854	MET	ttm-mtm	4.47	-3.21	12.16	7.97E-01	7.67E+02
855	MET	ttp-mtm	-0.68	-8.01	6.65	1.00E+00	9.63E+02
856	MET	ttt-mtm	-4.95	-14.81	4.90	9.21E-01	8.87E+02
857	MET	mtt-mtp	-2.46	-9.01	4.09	9.93E-01	9.56E+02
858	MET	off-mtp	2.65	-3.01	8.31	9.53E-01	9.18E+02
859	MET	ptm-mtp	-2.87	-13.21	7.48	1.00E+00	9.63E+02
860	MET	ptp-mtp	5.18	-5.61	15.97	9.44E-01	9.09E+02

861	MET	tpp-mtp	-3.71	-10.94	3.52	9.09E-01	8.75E+02
862	MET	tpt-mtp	-9.42	-23.88	5.04	6.40E-01	6.17E+02
863	MET	ttm-mtp	2.84	-4.36	10.03	9.89E-01	9.53E+02
864	MET	ttp-mtp	-2.31	-9.13	4.50	9.97E-01	9.61E+02
865	MET	ttt-mtp	-6.59	-16.07	2.89	5.30E-01	5.10E+02
866	MET	off-mtt	5.12	-1.75	11.98	4.07E-01	3.92E+02
867	MET	ptm-mtt	-0.40	-11.46	10.65	1.00E+00	9.63E+02
868	MET	ptp-mtt	7.64	-3.82	19.11	6.03E-01	5.80E+02
869	MET	tpp-mtt	-1.25	-9.46	6.96	1.00E+00	9.63E+02
870	MET	tpt-mtt	-6.96	-21.93	8.01	9.56E-01	9.21E+02
871	MET	ttm-mtt	5.30	-2.88	13.48	6.48E-01	6.24E+02
872	MET	ttp-mtt	0.15	-7.69	7.99	1.00E+00	9.63E+02
873	MET	ttt-mtt	-4.13	-14.37	6.11	9.87E-01	9.50E+02
874	MET	ptm-off	-5.52	-16.07	5.03	8.96E-01	8.63E+02
875	MET	ptp-off	2.53	-8.45	13.51	1.00E+00	9.63E+02
876	MET	tpp-off	-6.36	-13.88	1.15	2.05E-01	1.97E+02
877	MET	tpt-off	-12.07	-26.68	2.53	2.38E-01	2.29E+02
878	MET	ttm-off	0.18	-7.30	7.67	1.00E+00	9.63E+02
879	MET	ttp-off	-4.97	-12.08	2.15	5.24E-01	5.04E+02
880	MET	ttt-off	-9.24	-18.94	0.45	8.02E-02	7.72E+01
881	MET	ptp-ptm	8.05	-5.94	22.03	8.10E-01	7.80E+02
882	MET	tpp-ptm	-0.85	-12.31	10.62	1.00E+00	9.63E+02
883	MET	tpt-ptm	-6.56	-23.54	10.43	9.91E-01	9.54E+02
884	MET	ttm-ptm	5.70	-5.74	17.15	9.26E-01	8.92E+02
885	MET	ttp-ptm	0.55	-10.66	11.76	1.00E+00	9.63E+02
886	MET	ttt-ptm	-3.73	-16.73	9.28	1.00E+00	9.63E+02
887	MET	tpp-ptp	-8.89	-20.76	2.98	3.99E-01	3.84E+02
888	MET	tpt-ptp	-14.60	-31.85	2.65	2.05E-01	1.98E+02
889	MET	ttm-ptp	-2.34	-14.19	9.50	1.00E+00	9.63E+02
890	MET	ttp-ptp	-7.49	-19.11	4.12	6.56E-01	6.32E+02
891	MET	ttt-ptp	-11.77	-25.13	1.58	1.54E-01	1.49E+02
892	MET	tpt-tpp	-5.71	-20.99	9.57	9.93E-01	9.57E+02
893	MET	ttm-tpp	6.55	-2.18	15.28	3.97E-01	3.82E+02
894	MET	ttp-tpp	1.40	-7.03	9.82	1.00E+00	9.63E+02
895	MET	ttt-tpp	-2.88	-13.57	7.81	1.00E+00	9.63E+02
896	MET	ttm-tpt	12.26	-3.01	27.53	2.82E-01	2.72E+02
897	MET	ttp-tpt	7.11	-7.98	22.20	9.51E-01	9.16E+02
898	MET	ttt-tpt	2.83	-13.64	19.30	1.00E+00	9.63E+02
899	MET	ttp-ttm	-5.15	-13.54	3.24	7.29E-01	7.02E+02
900	MET	ttt-ttm	-9.43	-20.10	1.24	1.51E-01	1.45E+02
901	MET	ttt-ttp	-4.28	-14.69	6.14	9.84E-01	9.48E+02
902	PHE	m-85°-m-30°	3.05	0.57	5.52	7.03E-03	6.77E+00
903	PHE	off-m-30°	-1.77	-6.80	3.26	8.72E-01	8.40E+02
904	PHE	p90°-m-30°	-0.42	-3.46	2.62	9.96E-01	9.59E+02
905	PHE	t80°-m-30°	2.33	-0.22	4.88	9.33E-02	8.98E+01
906	PHE	off-m-85°	-4.82	-9.42	-0.22	3.46E-02	3.33E+01
907	PHE	p90°-m-85°	-3.47	-5.73	-1.20	2.85E-04	2.74E-01
908	PHE	t80°-m-85°	-0.72	-2.26	0.83	7.11E-01	6.85E+02

909	PHE	p90°-off	1.35	-3.58	6.28	9.45E-01	9.10E+02	
910	PHE	t80°-off	4.10	-0.54	8.75	1.12E-01	1.08E+02	
911	PHE	t80°-p90°	2.75	0.40	5.10	1.23E-02	1.19E+01	
912	PRO	Cγ Exo- Cγ Endo	7.94	6.79	9.09	0.00E+00	0.00E+00	*
913	PRO	off- Cγ Endo	4.83	2.94	6.72	6.65E-09	6.41E-06	*
914	PRO	off- Cγ Exo	-3.11	-4.99	-1.22	3.22E-04	3.10E-01	
915	SER	off-m	12.93	7.76	18.09	7.69E-10	7.41E-07	*
916	SER	p-m	7.82	6.63	9.01	0.00E+00	0.00E+00	*
917	SER	t-m	-6.16	-7.56	-4.75	7.11E-15	6.84E-12	*
918	SER	p-off	-5.11	-10.24	0.02	5.14E-02	4.95E+01	
919	SER	t-off	-19.08	-24.27	-13.90	3.51E-14	3.38E-11	*
920	SER	t-p	-13.98	-15.24	-12.71	0.00E+00	0.00E+00	*
921	THR	off-m	12.33	5.24	19.43	4.70E-05	4.53E-02	*
922	THR	p-m	7.06	5.94	8.18	0.00E+00	0.00E+00	*
923	THR	t-m	-12.75	-14.89	-10.60	0.00E+00	0.00E+00	*
924	THR	p-off	-5.27	-12.37	1.82	2.23E-01	2.15E+02	
925	THR	t-off	-25.08	-32.40	-17.76	3.06E-14	2.95E-11	*
926	THR	t-p	-19.80	-21.93	-17.68	0.00E+00	0.00E+00	*
927	TRP	m0°-m-90°	-1.98	-9.98	6.03	9.91E-01	9.54E+02	
928	TRP	m95°-m-90°	1.57	-5.41	8.55	9.94E-01	9.58E+02	
929	TRP	off-m-90°	-6.48	-15.37	2.41	3.23E-01	3.11E+02	
930	TRP	p-90°-m-90°	10.86	2.89	18.82	1.16E-03	1.12E+00	
931	TRP	p90°-m-90°	8.43	-0.87	17.73	1.05E-01	1.02E+02	
932	TRP	t-105°-m-90°	-0.55	-7.50	6.40	1.00E+00	9.63E+02	
933	TRP	m95°-m0°	3.55	-1.78	8.87	4.38E-01	4.21E+02	
934	TRP	off-m0°	-4.51	-12.17	3.15	5.91E-01	5.69E+02	
935	TRP	p-90°-m0°	12.83	6.27	19.40	1.78E-07	1.71E-04	*
936	TRP	p90°-m0°	10.40	2.27	18.53	3.08E-03	2.97E+00	
937	TRP	t-105°-m0°	1.43	-3.86	6.71	9.85E-01	9.49E+02	
938	TRP	off-m95°	-8.06	-14.64	-1.47	5.72E-03	5.51E+00	
939	TRP	p-90°-m95°	9.29	4.02	14.56	4.33E-06	4.17E-03	*
940	TRP	p90°-m95	6.86	-0.27	13.98	6.83E-02	6.58E+01	
941	TRP	t-105°-m95	-2.12	-5.67	1.43	5.73E-01	5.52E+02	
942	TRP	p-90°-off	17.34	9.72	24.96	4.61E-10	4.44E-07	*
943	TRP	p90°-off	14.91	5.91	23.92	2.19E-05	2.11E-02	*
944	TRP	t-105°-off	5.93	-0.61	12.48	1.05E-01	1.02E+02	
945	TRP	p90°-p-90°	-2.43	-10.52	5.67	9.75E-01	9.39E+02	
946	TRP	t-105°-p-90°	-11.41	-16.64	-6.18	2.80E-09	2.70E-06	*
947	TRP	t-105°-p90°	-8.98	-16.07	-1.88	3.63E-03	3.49E+00	
948	TYR	m-85°-m-30°	3.32	0.03	6.61	4.71E-02	4.54E+01	
949	TYR	off-m-30°	-10.72	-16.94	-4.50	2.59E-05	2.49E-02	*
950	TYR	p90°-m-30°	0.95	-3.00	4.89	9.66E-01	9.30E+02	
951	TYR	t80°-m-30°	4.54	1.17	7.92	2.26E-03	2.18E+00	
952	TYR	off-m-85°	-14.04	-19.60	-8.47	0.00E+00	0.00E+00	*
953	TYR	p90°-m-85°	-2.37	-5.17	0.42	1.40E-01	1.35E+02	
954	TYR	t80°-m-85°	1.23	-0.70	3.15	4.08E-01	3.93E+02	
955	TYR	p90°-off	11.66	5.69	17.64	9.55E-07	9.20E-04	*
956	TYR	t80°-off	15.26	9.64	20.88	0.00E+00	0.00E+00	*

957	TYR	t80°-p90°	3.60	0.70	6.50	6.29E-03	6.05E+00	
958	VAL	off-m	16.77	10.93	22.62	1.02E-12	9.87E-10	*
959	VAL	p-m	-3.81	-5.49	-2.13	3.32E-08	3.20E-05	*
960	VAL	t-m	-3.75	-4.71	-2.78	3.21E-14	3.09E-11	*
961	VAL	p-off	-20.59	-26.54	-14.63	2.91E-14	2.80E-11	*
962	VAL	t-off	-20.52	-26.31	-14.72	5.10E-14	4.91E-11	*
963	VAL	t-p	0.07	-1.44	1.57	9.99E-01	9.62E+02	*

A

Residue	All				Alpha Helix				Beta Sheet			
	FASPR	RASP	SCWRL4	SCWRL4v	FASPR	RASP	SCWRL4	SCWRL4v	FASPR	RASP	SCWRL4	SCWRL4v
VAL	94%	94%	95%	94%	95%	96%	97%	96%	95%	94%	95%	94%
THR	93%	93%	93%	93%	93%	93%	92%	93%	92%	92%	92%	91%
PHE	93%	91%	91%	89%	93%	90%	91%	87%	96%	95%	95%	93%
TYR	92%	89%	91%	88%	92%	87%	90%	86%	96%	94%	95%	93%
LEU	93%	91%	92%	90%	92%	90%	91%	89%	92%	90%	92%	90%
CYS	89%	92%	90%	90%	89%	93%	92%	92%	91%	92%	90%	90%
TRP	91%	86%	87%	83%	93%	87%	90%	86%	93%	88%	90%	86%
ILE	87%	86%	87%	86%	89%	88%	89%	88%	87%	85%	87%	86%
ASP	78%	78%	82%	80%	83%	82%	85%	83%	78%	77%	84%	81%
PRO	85%	87%	86%	84%	85%	87%	89%	87%	86%	87%	85%	84%
SER	74%	74%	75%	74%	65%	64%	62%	63%	72%	73%	75%	73%
MET	69%	62%	64%	59%	70%	63%	65%	61%	71%	65%	67%	62%
HIS	66%	63%	66%	62%	70%	66%	68%	64%	63%	60%	67%	61%
GLU	65%	61%	66%	62%	62%	58%	62%	58%	74%	69%	75%	71%
ASN	63%	62%	64%	62%	69%	69%	70%	69%	57%	57%	60%	57%
GLN	63%	61%	61%	59%	63%	62%	63%	61%	71%	66%	67%	65%
ARG	40%	36%	44%	35%	40%	37%	43%	35%	41%	38%	47%	38%
LYS	42%	40%	42%	38%	42%	41%	43%	40%	43%	41%	43%	40%
**Total**	**80%**	**79%**	**80%**	**78%**	**80%**	**78%**	**79%**	**77%**	**84%**	**83%**	**85%**	**83%**

B

Residue	Small (1-150 residues)				Medium (150-300 residues)				Large (300-3000 residues)			
	FASPR	RASP	SCWRL4	SCWRL4v	FASPR	RASP	SCWRL4	SCWRL4v	FASPR	RASP	SCWRL4	SCWRL4v
VAL	94%	94%	95%	94%	94%	95%	95%	95%	94%	94%	95%	94%
THR	92%	93%	92%	92%	93%	93%	93%	93%	93%	93%	93%	92%
PHE	93%	91%	91%	88%	93%	91%	91%	88%	93%	91%	92%	89%
TYR	92%	89%	90%	88%	92%	89%	90%	88%	93%	89%	91%	88%
LEU	92%	90%	90%	89%	92%	90%	91%	90%	93%	91%	92%	91%
TRP	90%	85%	83%	78%	91%	87%	87%	84%	91%	86%	87%	83%
CYS	88%	92%	91%	90%	89%	93%	90%	90%	89%	92%	89%	89%
ILE	87%	86%	86%	86%	87%	86%	87%	86%	88%	86%	88%	87%
PRO	83%	86%	85%	83%	85%	88%	87%	85%	85%	87%	86%	84%
ASP	76%	77%	80%	78%	78%	78%	82%	80%	79%	79%	82%	80%
SER	73%	72%	74%	73%	74%	73%	75%	74%	75%	75%	75%	74%
HIS	63%	61%	64%	59%	66%	63%	66%	62%	66%	63%	67%	62%
GLU	63%	61%	63%	60%	65%	61%	65%	61%	66%	62%	67%	62%
ASN	63%	61%	63%	62%	62%	62%	63%	61%	63%	62%	65%	62%
MET	65%	57%	55%	51%	69%	62%	62%	59%	70%	63%	67%	62%
GLN	60%	57%	57%	56%	63%	61%	61%	59%	64%	62%	62%	60%
ARG	33%	33%	37%	31%	39%	36%	42%	34%	42%	38%	47%	37%
LYS	41%	38%	40%	37%	42%	40%	41%	38%	42%	40%	43%	39%
**Total**	**79%**	**78%**	**78%**	**77%**	**80%**	**79%**	**80%**	**78%**	**80%**	**79%**	**81%**	**78%**

C

Residue	Low ACC (0-50)				Medium ACC (50-100)				High ACC (100-600)			
	FASPR	RASP	SCWRL4	SCWRL4v	FASPR	RASP	SCWRL4	SCWRL4v	FASPR	RASP	SCWRL4	SCWRL4v
VAL	95%	95%	96%	95%	90%	91%	91%	91%	84%	86%	86%	86%
PHE	95%	92%	94%	91%	90%	86%	84%	80%	78%	78%	75%	73%
THR	93%	94%	93%	93%	92%	92%	92%	92%	89%	89%	89%	89%
TYR	95%	91%	94%	91%	92%	88%	88%	84%	81%	80%	79%	76%
TRP	94%	89%	92%	87%	89%	82%	80%	75%	76%	71%	66%	65%
LEU	94%	92%	93%	91%	89%	87%	86%	85%	81%	81%	81%	81%
CYS	89%	93%	90%	90%	74%	83%	79%	80%	74%	70%	70%	70%
ILE	89%	88%	89%	88%	78%	78%	77%	77%	69%	69%	70%	69%
ASP	82%	81%	86%	83%	78%	79%	81%	79%	70%	71%	73%	72%
PRO	87%	89%	87%	85%	83%	86%	85%	83%	82%	85%	83%	82%
SER	76%	76%	77%	75%	72%	71%	71%	71%	66%	67%	69%	69%
GLU	78%	74%	83%	77%	66%	62%	65%	60%	51%	49%	50%	49%
MET	74%	66%	70%	65%	57%	52%	40%	39%	35%	31%	28%	27%
HIS	69%	66%	72%	66%	64%	62%	62%	59%	57%	55%	54%	54%
GLN	75%	72%	75%	72%	63%	61%	59%	57%	45%	44%	43%	43%
ASN	67%	67%	70%	67%	63%	62%	63%	61%	50%	49%	50%	49%
ARG	52%	46%	61%	46%	42%	38%	46%	36%	27%	27%	28%	26%
LYS	51%	46%	50%	42%	45%	42%	43%	40%	35%	35%	36%	35%
**Total**	**87%**	**86%**	**87%**	**85%**	**74%**	**73%**	**74%**	**71%**	**57%**	**56%**	**57%**	**56%**

Figure S1. Side-chain prediction programs accuracy based on discretised rotamer classes across amino acid residues (Canonical rotamers only). (A) Accuracy percentage in prediction of rotamer classes by FASPR, RASP, SCWRL4 and SCWRL4v in all, alpha helix and beta sheet shows three groups of residues. (B) Accuracy percentage in prediction of rotamer classes showed no role of protein size. (C) Accuracy percentage in prediction of rotamer classes based on solvent accessibility show more errors in at high ACC score range.

A

Residue	3-10 Helix				Turn				Pi Helix			
	FASPR	RASP	SCWRL4	SCWRL4v	FASPR	RASP	SCWRL4	SCWRL4v	FASPR	RASP	SCWRL4	SCWRL4v
THR	93%	92%	93%	92%	94%	94%	94%	95%	100%	100%	100%	100%
PHE	89%	86%	84%	82%	87%	83%	84%	79%	80%	70%	80%	70%
LEU	89%	87%	88%	87%	90%	89%	90%	89%	100%	100%	100%	100%
TYR	88%	84%	84%	84%	87%	83%	83%	79%	100%	80%	80%	80%
CYS	86%	92%	86%	87%	80%	90%	85%	85%	100%	100%	100%	100%
TRP	83%	74%	73%	68%	81%	74%	70%	64%	50%	0%	100%	100%
VAL	83%	84%	85%	84%	89%	89%	90%	90%	90%	100%	100%	100%
SER	81%	79%	80%	79%	79%	79%	79%	78%	50%	100%	100%	100%
ASP	80%	78%	81%	79%	62%	67%	70%	68%	80%	90%	90%	90%
ILE	78%	75%	77%	75%	82%	79%	81%	80%	71%	57%	71%	71%
PRO	68%	78%	76%	73%	75%	78%	76%	74%	50%	100%	50%	50%
HIS	62%	60%	59%	58%	60%	58%	60%	55%	50%	25%	50%	50%
GLU	60%	60%	60%	57%	60%	56%	60%	55%	80%	80%	60%	80%
MET	59%	58%	56%	51%	58%	53%	53%	50%	67%	33%	33%	33%
ASN	55%	54%	51%	50%	52%	51%	52%	50%	50%	50%	50%	75%
GLN	48%	48%	46%	45%	43%	44%	43%	43%	67%	33%	33%	67%
ARG	42%	41%	45%	39%	38%	36%	43%	36%	67%	67%	67%	33%
LYS	38%	34%	35%	33%	35%	33%	33%	32%	60%	40%	80%	40%
**Total**	**72%**	**72%**	**72%**	**70%**	**69%**	**69%**	**69%**	**67%**	**78%**	**74%**	**80%**	**78%**

B

Residue	Beta Bridge				Bend				Coil			
	FASPR	RASP	SCWRL4	SCWRL4v	FASPR	RASP	SCWRL4	SCWRL4v	FASPR	RASP	SCWRL4	SCWRL4v
VAL	96%	97%	96%	95%	92%	93%	93%	92%	92%	93%	93%	93%
CYS	95%	95%	93%	96%	83%	90%	89%	89%	88%	91%	87%	87%
THR	93%	93%	93%	92%	92%	92%	92%	93%	92%	93%	93%	92%
TYR	93%	90%	91%	89%	86%	84%	86%	82%	90%	88%	89%	87%
PHE	93%	91%	91%	90%	90%	86%	87%	85%	90%	88%	89%	86%
LEU	91%	90%	90%	90%	90%	89%	89%	89%	89%	88%	89%	88%
TRP	86%	83%	83%	78%	82%	82%	74%	72%	87%	84%	84%	81%
ILE	85%	88%	84%	83%	84%	82%	83%	82%	85%	85%	85%	84%
PRO	79%	79%	77%	76%	80%	81%	80%	79%	77%	78%	76%	75%
SER	78%	79%	81%	81%	77%	77%	78%	77%	77%	77%	80%	78%
MET	76%	66%	71%	65%	61%	55%	55%	50%	63%	57%	54%	52%
ASP	75%	79%	80%	79%	74%	74%	76%	74%	79%	79%	83%	81%
GLU	66%	66%	69%	67%	57%	56%	60%	58%	62%	59%	64%	60%
ASN	61%	59%	63%	59%	55%	54%	55%	53%	60%	59%	61%	60%
HIS	60%	59%	59%	59%	60%	58%	60%	58%	62%	62%	65%	61%
ARG	53%	49%	54%	48%	43%	40%	43%	38%	45%	44%	48%	41%
GLN	51%	57%	51%	50%	46%	44%	44%	43%	51%	49%	50%	49%
LYS	34%	34%	31%	31%	35%	32%	35%	31%	34%	33%	34%	31%
**Total**	**79%**	**79%**	**79%**	**78%**	**73%**	**72%**	**73%**	**72%**	**76%**	**76%**	**77%**	**75%**

Figure S2. Side-chain prediction programs accuracy based on discretised rotamer classes across amino acid residues according to secondary structures. (A) Accuracy percentage in prediction of rotamer classes by FASPR, RASP, SCWRL4 and SCWRL4v in 3-10 helix, turn and pi helix shows three groups of residues. **(B)** Accuracy percentage in prediction of rotamer classes by FASPR, RASP, SCWRL4 and SCWRL4v in bend, beta bridge and coils shows three groups of residues.

A

Residue	3-10 Helix				Turn				Pi Helix			
	FASPR	RASP	SCWRL4	SCWRL4v	FASPR	RASP	SCWRL4	SCWRL4v	FASPR	RASP	SCWRL4	SCWRL4v
THR	93%	92%	94%	93%	95%	95%	95%	95%	100%	100%	100%	100%
PHE	90%	87%	85%	83%	89%	84%	85%	80%	89%	78%	89%	78%
LEU	93%	91%	92%	91%	94%	92%	93%	92%	100%	100%	100%	100%
TYR	88%	84%	85%	84%	88%	84%	84%	80%	100%	80%	80%	80%
CYS	87%	93%	86%	88%	81%	90%	86%	85%	100%	100%	100%	100%
TRP	88%	78%	78%	73%	84%	77%	73%	66%	50%	0%	100%	100%
VAL	83%	84%	86%	84%	90%	90%	91%	91%	90%	100%	100%	100%
SER	82%	79%	80%	80%	80%	80%	79%	78%	50%	100%	100%	100%
ASP	82%	80%	83%	82%	63%	69%	71%	69%	89%	100%	100%	100%
ILE	81%	77%	80%	78%	84%	81%	83%	82%	71%	57%	71%	71%
PRO	76%	86%	85%	81%	82%	86%	84%	81%	50%	100%	50%	50%
HIS	66%	63%	61%	60%	62%	61%	62%	57%	67%	33%	67%	67%
GLU	64%	64%	64%	61%	63%	59%	63%	58%	80%	80%	60%	80%
MET	66%	64%	62%	56%	64%	56%	57%	54%	100%	50%	50%	50%
ASN	61%	59%	56%	55%	56%	56%	57%	55%	50%	50%	50%	75%
GLN	62%	61%	58%	55%	53%	53%	51%	50%	67%	33%	33%	67%
ARG	39%	35%	44%	34%	36%	32%	42%	33%	50%	50%	50%	0%
LYS	43%	39%	40%	38%	41%	39%	39%	37%	75%	50%	100%	50%
**Total**	**77%**	**76%**	**76%**	**74%**	**73%**	**73%**	**73%**	**71%**	**83%**	**78%**	**84%**	**83%**

B

Residue	Beta Bridge				Bend				Coil			
	FASPR	RASP	SCWRL4	SCWRL4v	FASPR	RASP	SCWRL4	SCWRL4v	FASPR	RASP	SCWRL4	SCWRL4v
VAL	96%	97%	96%	95%	93%	94%	94%	93%	93%	94%	94%	94%
CYS	95%	95%	93%	96%	84%	90%	89%	89%	89%	92%	88%	88%
THR	94%	94%	94%	93%	93%	92%	93%	93%	93%	93%	94%	93%
TYR	95%	91%	93%	90%	88%	86%	87%	83%	92%	89%	91%	88%
PHE	94%	92%	93%	92%	92%	88%	89%	86%	92%	90%	90%	87%
LEU	95%	94%	94%	94%	93%	92%	93%	92%	93%	92%	93%	92%
TRP	92%	88%	89%	82%	86%	85%	79%	76%	90%	86%	88%	83%
ILE	86%	89%	85%	84%	86%	84%	85%	84%	87%	86%	86%	85%
PRO	88%	88%	85%	85%	88%	89%	88%	86%	86%	88%	86%	85%
SER	79%	81%	83%	82%	78%	78%	79%	78%	78%	78%	81%	80%
MET	79%	67%	74%	67%	66%	58%	59%	53%	68%	62%	59%	56%
ASP	78%	82%	84%	83%	76%	76%	78%	76%	81%	81%	84%	82%
GLU	70%	69%	72%	71%	61%	59%	64%	61%	67%	64%	69%	64%
ASN	66%	65%	69%	65%	61%	60%	61%	59%	66%	65%	68%	66%
HIS	63%	61%	61%	61%	63%	60%	63%	60%	65%	64%	67%	64%
ARG	43%	35%	44%	38%	38%	35%	41%	34%	41%	37%	45%	36%
GLN	67%	75%	63%	63%	59%	56%	55%	52%	63%	59%	60%	58%
LYS	40%	39%	36%	34%	41%	37%	41%	36%	40%	38%	39%	36%
**Total**	**83%**	**82%**	**83%**	**81%**	**77%**	**76%**	**77%**	**76%**	**81%**	**80%**	**81%**	**79%**

Figure S3. Side-chain prediction programs accuracy based on discretised rotamer classes across amino acid residues according to secondary structures (Canonical rotamers only). (A) Accuracy percentage in prediction of rotamer classes by FASPR, RASP, SCWRL4 and SCWRL4v in 3-10 helix, turn and pi helix shows three groups of residues. **(B)** Accuracy percentage in prediction of rotamer classes by FASPR, RASP, SCWRL4 and SCWRL4v in bend, beta bridge and coils shows three groups of residues.

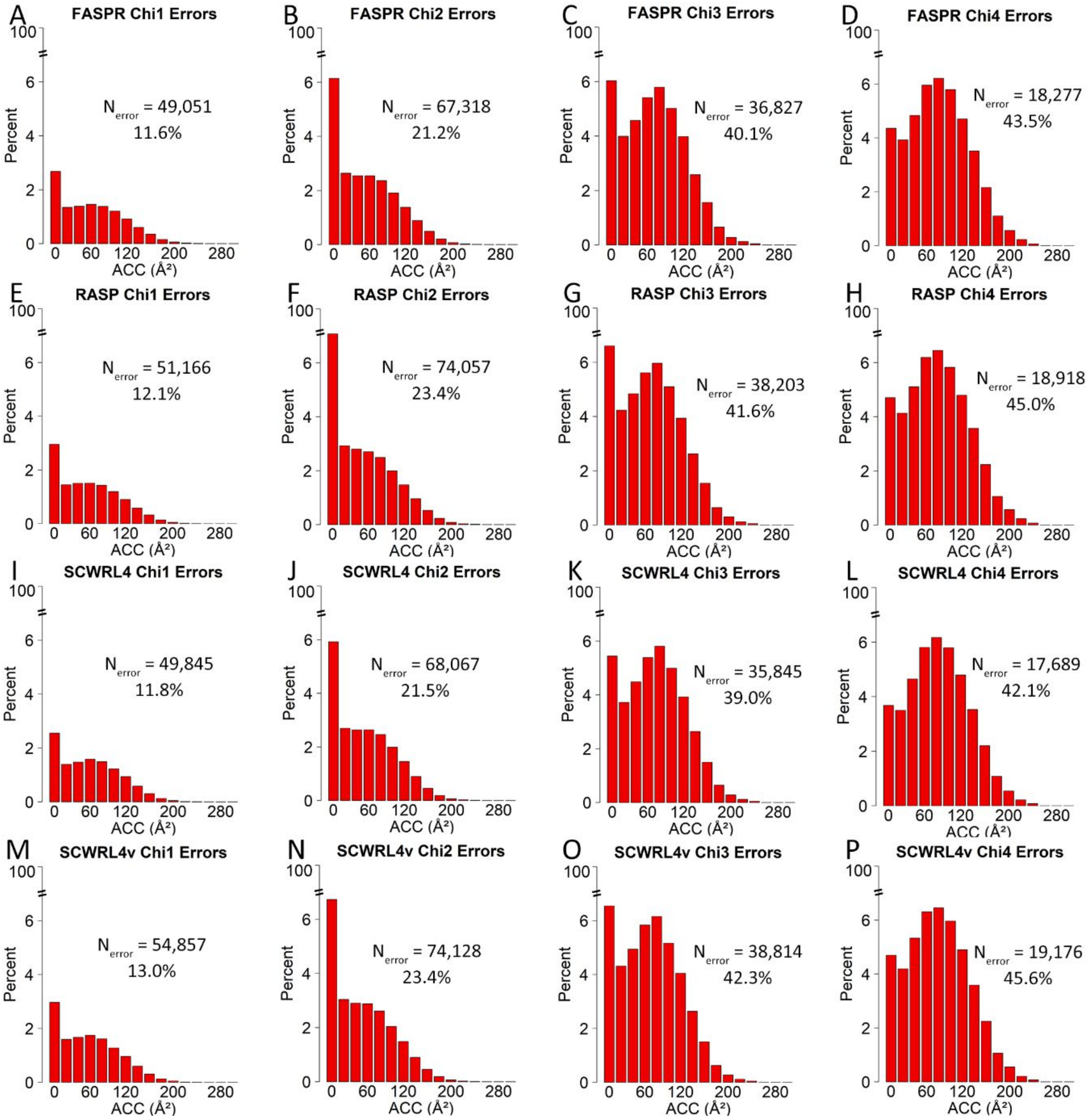

Figure S4. Errors in side-chain prediction programs according to solvent accessibility as a function of Chi angle deviation by 30°. Percent angular errors in torsional angles following analysis (A-D) with FASPR, (E-H) RASP, (I-L) SCWRL4, and (M-P) SCWRL4v. The y-axis represents percentage of errors in each bin divided by total number of cases for that Chi angle thus it gives more weight to the number of cases in each bin. Each bar is labelled by the lower limit of a 20 Å2 ACC interval divided from 0 to 320 Å2. SCWRL4v represents use of SCWRL4 program with fixed rotamer library search using -v option to disable sub-rotamers.

	Residue	FASPR				RASP				SCWRL4				SCWRL4v			
		Chi1	Chi2	Chi3	Chi4	Chi1	Chi2	Chi3	Chi4	Chi1	Chi2	Chi3	Chi4	Chi1	Chi2	Chi3	Chi4
A		Errors at all ACC values															
	ARG	14.3%	14.7%	29.5%	33.7%	14.5%	16.5%	30.7%	34.7%	14.5%	15.1%	28.8%	32.2%	16.5%	17.1%	31.3%	34.8%
	ASN	11.7%	27.4%			12.5%	28.0%			11.4%	27.4%			12.3%	28.4%		
	ASP	12.2%	19.9%			12.5%	20.1%			10.5%	17.6%			12.0%	19.2%		
	CYS	10.7%				7.6%				9.9%				9.7%			
	GLN	15.1%	21.5%	35.2%		15.1%	22.6%	35.9%		14.8%	21.8%	34.9%		16.0%	22.5%	36.1%	
	GLU	17.6%	21.1%	30.1%		18.8%	23.0%	30.1%		18.3%	19.9%	29.2%		19.5%	22.0%	30.8%	
	HIS	8.4%	31.6%			9.8%	32.5%			9.6%	30.0%			10.7%	31.7%		
	ILE	3.5%	10.6%			4.2%	11.5%			3.5%	10.8%			3.9%	11.5%		
	LEU	5.9%	9.5%			7.2%	10.7%			6.9%	10.1%			7.7%	11.1%		
	LYS	15.8%	15.1%	20.6%	26.5%	15.0%	17.0%	21.3%	26.8%	16.1%	15.6%	19.7%	26.8%	16.9%	16.3%	20.8%	27.4%
	MET	9.1%	11.8%	24.6%		11.2%	14.8%	27.2%		11.2%	15.4%	26.4%		12.7%	17.0%	28.1%	
	PHE	3.1%	19.4%			4.5%	23.7%			5.0%	21.8%			6.1%	22.6%		
	PRO	15.1%	16.5%			13.3%	14.8%			14.2%	15.7%			15.3%	16.8%		
	SER	20.9%				21.0%				20.5%				21.1%			
	THR	7.2%				7.1%				7.1%				7.3%			
	TRP	4.6%	11.4%			6.5%	15.7%			6.9%	15.6%			8.3%	18.7%		
	TYR	4.1%	20.1%			6.1%	23.1%			5.6%	21.7%			7.2%	23.0%		
	VAL	5.7%				5.6%				5.1%				5.6%			
B		Errors at zero ACC values															
	ARG	9.1%	15.2%	18.8%	30.6%	9.8%	16.4%	24.3%	31.3%	7.0%	11.8%	18.1%	25.1%	10.7%	16.7%	22.8%	31.2%
	ASN	4.8%	26.5%			5.5%	26.9%			3.6%	25.6%			4.8%	27.4%		
	ASP	6.7%	21.7%			7.1%	21.0%			3.4%	16.5%			4.8%	19.9%		
	CYS	5.9%				4.8%				7.2%				7.1%			
	GLN	9.4%	12.6%	33.3%		8.3%	12.0%	33.5%		5.5%	8.1%	29.8%		7.1%	9.8%	32.9%	
	GLU	8.0%	13.8%	30.7%		11.0%	15.8%	28.6%		7.5%	10.6%	24.4%		9.7%	13.9%	30.3%	
	HIS	3.1%	32.9%			3.9%	33.0%			2.7%	27.8%			2.7%	31.0%		
	ILE	1.7%	7.5%			2.6%	8.8%			2.1%	7.2%			2.2%	8.1%		
	LEU	3.2%	8.0%			4.7%	9.2%			3.4%	7.7%			4.1%	8.9%		
	LYS	2.6%	11.0%	14.6%	28.6%	7.4%	14.2%	22.7%	32.0%	3.6%	9.7%	16.9%	25.5%	7.4%	11.0%	20.1%	30.5%
	MET	5.3%	7.3%	20.8%		8.0%	10.4%	24.2%		5.7%	6.5%	20.3%		7.5%	9.5%	23.9%	
	PHE	1.2%	19.1%			1.4%	23.4%			1.1%	20.4%			1.3%	21.2%		
	PRO	12.7%	14.2%			11.2%	13.0%			10.9%	12.6%			13.1%	14.7%		
	SER	18.4%				18.4%				17.7%				19.9%			
	THR	6.1%				5.9%				5.5%				6.0%			
	TRP	2.9%	7.4%			3.9%	9.6%			2.8%	5.8%			3.7%	8.3%		
	TYR	2.2%	19.4%			4.4%	22.8%			2.1%	19.7%			3.2%	21.1%		
	VAL	3.8%				4.0%				3.3%				3.6%			
C		Difference in Errors															
	ARG	-5.1%	0.5%	-10.7%	-3.1%	-4.7%	-0.1%	-6.4%	-3.4%	-7.5%	-3.3%	-10.7%	-7.1%	-5.8%	-0.3%	-8.4%	-3.6%
	ASN	-6.9%	-0.9%			-7.0%	-1.1%			-7.9%	-1.8%			-7.5%	-1.0%		
	ASP	-5.4%	1.8%			-5.4%	0.9%			-7.2%	-1.2%			-7.3%	0.7%		
	CYS	-4.7%				-2.8%				-2.7%				-2.6%			
	GLN	-5.7%	-8.8%	-1.9%		-6.7%	-10.6%	-2.5%		-9.3%	-13.8%	-5.0%		-8.9%	-12.7%	-3.2%	
	GLU	-9.6%	-7.3%	0.6%		-7.7%	-7.2%	-1.5%		-10.8%	-9.4%	-4.8%		-9.7%	-8.1%	-0.5%	
	HIS	-5.4%	1.3%			-5.8%	0.5%			-6.9%	-2.2%			-8.0%	-0.7%		
	ILE	-1.8%	-3.1%			-1.6%	-2.6%			-1.4%	-3.6%			-1.7%	-3.4%		
	LEU	-2.7%	-1.6%			-2.6%	-1.6%			-3.5%	-2.3%			-3.6%	-2.2%		
	LYS	-13.1%	-4.1%	-6.0%	2.1%	-7.6%	-2.8%	1.4%	5.2%	-12.5%	-5.9%	-2.8%	-1.3%	-9.5%	-5.3%	-0.7%	3.1%
	MET	-3.9%	-4.4%	-3.8%		-3.3%	-4.4%	-3.0%		-5.5%	-8.9%	-6.1%		-5.2%	-7.5%	-4.2%	
	PHE	-2.0%	-0.4%			-3.1%	-0.3%			-3.9%	-1.3%			-4.8%	-1.4%		
	PRO	-2.4%	-2.4%			-2.1%	-1.8%			-3.3%	-3.1%			-2.3%	-2.1%		
	SER	-2.5%				-2.6%				-2.7%				-1.2%			
	THR	-1.1%				-1.1%				-1.6%				-1.3%			
	TRP	-1.8%	-4.0%			-2.6%	-6.1%			-4.1%	-9.8%			-4.5%	-10.5%		
	TYR	-1.9%	-0.7%			-1.7%	-0.3%			-3.4%	-1.9%			-4.0%	-1.9%		
	VAL	-1.8%				-1.6%				-1.8%				-2.0%			

Figure S5. Side-chain prediction programs errors based on Chi angles deviation by 30° from original, decomposed by amino acids. (A) Error percentage in prediction of Chi angles by FASPR, RASP, SCWRL4 and SCWRL4v at all ACC values. (B) Error percentage in prediction of Chi angles by FASPR, RASP, SCWRL4 and SCWRL4v at zero ACC values. (C) Differences in Error percentages between residues at zero ACC values and those at all ACC values.

	Residue	FASPR				RASP				SCWRL4				SCWRL4v			
		Chi1	Chi2	Chi3	Chi4	Chi1	Chi2	Chi3	Chi4	Chi1	Chi2	Chi3	Chi4	Chi1	Chi2	Chi3	Chi4
A		Amino acids contributions to errors at all ACC values															
	ARG	5.2%	6.5%	23.4%	53.0%	5.2%	6.5%	23.5%	53.2%	5.2%	6.5%	23.3%	52.3%	5.3%	6.5%	23.6%	53.0%
	ASN	5.2%	7.7%			5.2%	7.6%			5.1%	7.7%			5.1%	7.7%		
	ASP	7.2%	9.7%			7.2%	9.6%			7.1%	9.4%			7.1%	9.5%		
	CYS	1.6%				1.5%				1.6%				1.5%			
	GLN	4.0%	5.4%	19.4%		4.0%	5.4%	19.4%		4.0%	5.4%	19.5%		4.0%	5.3%	19.4%	
	GLU	6.8%	8.7%	29.4%		6.9%	8.8%	29.0%		6.8%	8.6%	29.2%		6.9%	8.6%	29.2%	
	HIS	2.9%	4.8%			3.0%	4.8%			3.0%	4.7%			3.0%	4.7%		
	ILE	6.9%	9.1%			6.9%	9.0%			6.9%	9.1%			6.8%	9.0%		
	LEU	11.7%	14.9%			11.8%	14.8%			11.8%	15.0%			11.8%	14.9%		
	LYS	5.3%	6.4%	20.4%	47.0%	5.2%	6.4%	20.4%	46.8%	5.3%	6.4%	20.3%	47.7%	5.3%	6.4%	20.2%	47.0%
	MET	1.7%	2.1%	7.5%		1.7%	2.2%	7.6%		1.7%	2.2%	7.7%		1.7%	2.2%	7.7%	
	PHE	5.1%	7.5%			5.2%	7.8%			5.2%	7.7%			5.2%	7.7%		
	PRO	6.4%	8.0%			6.3%	7.7%			6.4%	7.9%			6.4%	7.9%		
	SER	7.8%				7.8%				7.8%				7.8%			
	THR	6.7%				6.7%				6.7%				6.7%			
	TRP	1.9%	2.5%			1.9%	2.5%			1.9%	2.6%			1.9%	2.6%		
	TYR	4.6%	6.7%			4.6%	6.9%			4.6%	6.8%			4.7%	6.9%		
	VAL	9.0%				8.9%				8.9%				8.9%			
B		Amino acids contributions to errors at zero ACC values															
	ARG	0.6%	1.0%	9.1%	66.3%	0.6%	1.0%	9.5%	65.5%	0.6%	1.0%	9.3%	65.6%	0.7%	1.0%	9.4%	65.9%
	ASN	2.1%	4.0%			2.1%	3.9%			2.1%	3.9%			2.1%	4.0%		
	ASP	2.1%	3.5%			2.1%	3.4%			2.0%	3.3%			2.0%	3.4%		
	CYS	3.5%				3.5%				3.6%				3.6%			
	GLN	1.1%	1.6%	19.1%		1.1%	1.6%	18.6%		1.1%	1.6%	18.7%		1.1%	1.6%	18.5%	
	GLU	1.2%	1.8%	20.2%		1.2%	1.8%	19.1%		1.2%	1.8%	19.0%		1.2%	1.8%	19.6%	
	HIS	1.3%	2.7%			1.3%	2.6%			1.3%	2.5%			1.3%	2.6%		
	ILE	14.7%	22.3%			14.7%	22.2%			14.8%	22.4%			14.7%	22.2%		
	LEU	21.4%	32.1%			21.5%	31.9%			21.5%	32.2%			21.4%	32.1%		
	LYS	0.3%	0.5%	4.5%	33.7%	0.3%	0.5%	4.9%	34.5%	0.3%	0.5%	4.8%	34.4%	0.3%	0.5%	4.7%	34.1%
	MET	3.1%	4.6%	47.1%		3.2%	4.6%	47.9%		3.1%	4.6%	48.2%		3.2%	4.6%	47.9%	
	PHE	7.9%	13.8%			7.9%	14.3%			7.9%	14.1%			7.9%	14.0%		
	PRO	3.3%	4.8%			3.2%	4.6%			3.2%	4.7%			3.3%	4.8%		
	SER	7.3%				7.3%				7.3%				7.4%			
	THR	6.1%				6.0%				6.1%				6.0%			
	TRP	1.5%	2.2%			1.5%	2.2%			1.5%	2.2%			1.5%	2.2%		
	TYR	3.0%	5.2%			3.0%	5.3%			3.0%	5.2%			3.0%	5.2%		
	VAL	19.5%				19.4%				19.5%				19.4%			
C		Difference in amino acids contributions to errors															
	ARG	-4.6%	-5.5%	-14.2%	13.4%	-4.6%	-5.5%	-14.0%	12.2%	-4.6%	-5.5%	-14.0%	13.3%	-4.7%	-5.5%	-14.2%	12.9%
	ASN	-3.0%	-3.7%			-3.0%	-3.7%			-3.0%	-3.7%			-3.0%	-3.7%		
	ASP	-5.2%	-6.2%			-5.2%	-6.1%			-5.1%	-6.1%			-5.1%	-6.1%		
	CYS	2.0%				2.0%				2.0%				2.0%			
	GLN	-2.9%	-3.7%	-0.3%		-2.9%	-3.7%	-0.8%		-3.0%	-3.8%	-0.8%		-3.0%	-3.8%	-0.9%	
	GLU	-5.6%	-6.9%	-9.2%		-5.6%	-6.9%	-10.0%		-5.6%	-6.8%	-10.1%		-5.6%	-6.8%	-9.6%	
	HIS	-1.6%	-2.1%			-1.7%	-2.2%			-1.7%	-2.2%			-1.7%	-2.2%		
	ILE	7.8%	13.2%			7.8%	13.1%			7.9%	13.3%			7.9%	13.2%		
	LEU	9.7%	17.2%			9.8%	17.1%			9.7%	17.3%			9.7%	17.2%		
	LYS	-4.9%	-5.9%	-15.9%	-13.4%	-4.9%	-5.9%	-15.5%	-12.2%	-4.9%	-5.9%	-15.6%	-13.3%	-4.9%	-5.9%	-15.4%	-12.9%
	MET	1.4%	2.4%	39.6%		1.5%	2.5%	40.3%		1.4%	2.3%	40.5%		1.4%	2.4%	40.1%	
	PHE	2.8%	6.3%			2.7%	6.5%			2.7%	6.4%			2.7%	6.3%		
	PRO	-3.2%	-3.3%			-3.1%	-3.1%			-3.1%	-3.2%			-3.1%	-3.2%		
	SER	-0.5%				-0.6%				-0.5%				-0.4%			
	THR	-0.7%				-0.7%				-0.7%				-0.6%			
	TRP	-0.4%	-0.2%			-0.4%	-0.3%			-0.4%	-0.4%			-0.4%	-0.4%		
	TYR	-1.6%	-1.5%			-1.6%	-1.6%			-1.6%	-1.6%			-1.7%	-1.6%		
	VAL	10.5%				10.5%				10.5%				10.5%			

Figure S6. Amino acid contribution to errors in side-chain prediction programs based on Chi angles deviation by 30° from original. (A) Amino acid contribution percentage in prediction of Chi angles by FASPR, RASP, SCWRL4 and SCWRL4v at all ACC values. (B) Amino acid contribution percentage in prediction of Chi angles by FASPR, RASP, SCWRL4 and SCWRL4v at zero ACC values. (C) Differences in amino acid contribution percentage between residues at zero ACC values and those at all ACC values.

	Residue	FASPR				RASP				SCWRL4				SCWRL4v			
		Chi1	Chi2	Chi3	Chi4	Chi1	Chi2	Chi3	Chi4	Chi1	Chi2	Chi3	Chi4	Chi1	Chi2	Chi3	Chi4
A		Errors at low ACC values															
	ARG	9.6%	13.5%	23.9%	31.0%	11.3%	15.5%	26.4%	32.7%	9.0%	12.5%	21.7%	27.3%	12.6%	16.3%	26.4%	32.3%
	ASN	6.5%	25.5%			7.4%	26.2%			5.6%	25.1%			6.9%	26.6%		
	ASP	8.2%	19.8%			8.9%	19.9%			5.7%	16.0%			7.5%	18.3%		
	CYS	9.9%				7.0%				9.3%				9.1%			
	GLN	9.7%	13.9%	32.9%		10.1%	15.1%	33.8%		8.6%	12.8%	31.6%		10.0%	14.7%	33.5%	
	GLU	11.1%	15.9%	29.0%		12.9%	17.8%	28.9%		10.4%	12.4%	25.5%		12.7%	15.9%	28.9%	
	HIS	4.5%	30.8%			5.8%	32.0%			5.2%	27.8%			6.4%	30.5%		
	ILE	2.7%	9.3%			3.3%	10.4%			2.7%	9.4%			3.1%	10.2%		
	LEU	4.6%	8.6%			6.1%	9.8%			5.5%	8.9%			6.3%	10.1%		
	LYS	7.1%	11.7%	19.7%	25.9%	8.3%	13.7%	22.1%	26.6%	8.9%	12.4%	19.2%	25.6%	10.6%	13.9%	21.6%	27.9%
	MET	7.4%	9.8%	22.7%		9.8%	12.7%	25.5%		9.0%	11.8%	24.0%		10.8%	14.0%	26.0%	
	PHE	1.8%	18.7%			3.1%	23.3%			3.0%	20.7%			3.8%	21.6%		
	PRO	13.7%	15.1%			12.3%	13.8%			13.1%	14.6%			14.4%	15.8%		
	SER	19.9%				19.9%				19.2%				20.1%			
	THR	6.7%				6.4%				6.5%				6.8%			
	TRP	3.2%	9.1%			4.8%	13.1%			4.2%	11.9%			5.6%	15.4%		
	TYR	2.4%	19.2%			4.4%	22.5%			3.2%	20.4%			4.5%	21.9%		
	VAL	4.8%				4.8%				4.3%				4.8%			
B		Errors at medium ACC values															
	ARG	11.9%	13.6%	28.6%	33.2%	12.6%	15.0%	30.3%	34.4%	13.7%	14.9%	28.8%	32.1%	15.6%	16.8%	31.6%	34.7%
	ASN	12.9%	27.2%			13.4%	27.6%			13.3%	27.5%			14.0%	28.3%		
	ASP	12.6%	18.9%			12.8%	19.1%			11.2%	17.3%			13.1%	18.8%		
	CYS	21.3%				15.2%				18.0%				17.4%			
	GLN	15.7%	21.9%	35.5%		15.4%	23.0%	36.3%		15.8%	23.6%	35.6%		17.3%	24.2%	36.6%	
	GLU	16.6%	20.6%	29.4%		18.4%	22.6%	29.4%		18.8%	19.8%	29.3%		20.2%	22.1%	30.6%	
	HIS	9.3%	32.3%			10.8%	32.7%			11.7%	32.0%			13.1%	33.1%		
	ILE	6.7%	16.7%			7.4%	16.4%			6.7%	17.5%			7.2%	17.5%		
	LEU	9.7%	12.3%			10.9%	13.5%			11.8%	14.0%			12.5%	14.8%		
	LYS	11.7%	14.0%	21.5%	25.7%	12.2%	16.1%	22.1%	26.1%	14.7%	15.3%	20.5%	26.2%	15.7%	16.1%	21.7%	26.5%
	MET	13.3%	16.6%	29.5%		14.0%	19.8%	31.2%		17.4%	26.9%	33.6%		18.0%	26.9%	34.1%	
	PHE	4.6%	20.6%			8.2%	24.6%			10.6%	24.7%			13.0%	25.9%		
	PRO	16.4%	17.9%			14.0%	15.7%			15.0%	16.5%			16.0%	17.5%		
	SER	22.4%				22.8%				22.7%				22.8%			
	THR	7.4%				7.4%				7.6%				7.7%			
	TRP	5.2%	12.3%			7.8%	18.8%			9.2%	21.4%			10.9%	25.0%		
	TYR	4.4%	20.7%			7.1%	23.9%			7.5%	23.4%			9.8%	24.4%		
	VAL	9.2%				9.0%				8.6%				9.2%			
C		Errors at high ACC values															
	ARG	20.0%	16.6%	34.7%	36.4%	18.8%	18.7%	34.6%	36.7%	19.6%	17.4%	34.1%	36.2%	20.5%	18.0%	34.7%	36.9%
	ASN	21.3%	32.5%			22.1%	33.1%			21.1%	32.6%			21.5%	32.9%		
	ASP	19.0%	22.0%			19.2%	22.5%			18.3%	21.6%			18.8%	21.9%		
	CYS	22.9%				25.0%				25.0%				25.0%			
	GLN	21.3%	30.3%	38.0%		21.3%	31.5%	38.5%		21.6%	30.7%	38.6%		22.2%	30.2%	39.1%	
	GLU	24.0%	25.9%	32.0%		24.0%	27.8%	32.0%		24.1%	26.1%	32.3%		24.2%	26.8%	32.5%	
	HIS	18.3%	33.1%			19.3%	33.6%			18.8%	33.4%			19.0%	33.5%		
	ILE	12.9%	19.6%			12.6%	20.5%			11.4%	20.6%			11.8%	20.9%		
	LEU	16.2%	18.2%			16.5%	18.6%			16.3%	18.6%			16.2%	18.5%		
	LYS	22.2%	17.4%	20.2%	27.4%	20.0%	19.2%	20.2%	27.6%	20.3%	17.3%	19.3%	27.8%	20.5%	17.5%	19.6%	27.9%
	MET	19.7%	23.9%	35.7%		21.9%	26.9%	37.2%		21.8%	27.3%	37.0%		22.8%	27.5%	37.4%	
	PHE	15.2%	25.8%			14.4%	27.1%			17.1%	28.3%			17.9%	27.8%		
	PRO	17.5%	18.7%			15.4%	16.8%			16.9%	18.2%			17.5%	18.8%		
	SER	26.0%				25.7%				24.6%				24.4%			
	THR	11.1%				10.8%				10.7%				10.5%			
	TRP	13.2%	23.6%			15.5%	26.3%			19.2%	27.6%			20.1%	28.2%		
	TYR	12.2%	24.0%			13.1%	25.4%			14.0%	25.5%			15.8%	26.5%		
	VAL	14.7%				13.7%				13.7%				13.7%			

Figure S7. Side-chain prediction programs errors based on Chi angles deviation by 30° from original, decomposed by amino acids. (A) Error percentage in prediction of Chi angles by FASPR, RASP, SCWRL4 and SCWRL4v at low ACC values. (B) Error percentage in prediction of Chi angles by FASPR, RASP, SCWRL4 and SCWRL4v at medium ACC values. (C) Error percentage in prediction of Chi angles by FASPR, RASP, SCWRL4 and SCWRL4v at high ACC values.

	Residue	FASPR				RASP				SCWRL4				SCWRL4v			
		Chi1	Chi2	Chi3	Chi4	Chi1	Chi2	Chi3	Chi4	Chi1	Chi2	Chi3	Chi4	Chi1	Chi2	Chi3	Chi4
A		Amino acids contributions to errors at low ACC values															
	ARG	2.6%	3.5%	20.3%	62.2%	2.6%	3.6%	20.6%	62.6%	2.6%	3.5%	20.2%	61.1%	2.7%	3.6%	20.6%	62.0%
	ASN	3.9%	6.4%			3.9%	6.4%			3.9%	6.4%			3.9%	6.4%		
	ASP	4.9%	7.3%			4.9%	7.1%			4.7%	6.9%			4.8%	7.0%		
	CYS	2.5%				2.4%				2.4%				2.4%			
	GLN	2.5%	3.5%	22.5%		2.5%	3.4%	22.3%		2.5%	3.4%	22.5%		2.5%	3.4%	22.2%	
	GLU	3.3%	4.5%	27.0%		3.3%	4.5%	26.4%		3.2%	4.3%	26.3%		3.3%	4.4%	26.5%	
	HIS	2.6%	4.7%			2.6%	4.7%			2.6%	4.5%			2.6%	4.6%		
	ILE	9.8%	13.7%			9.8%	13.6%			9.8%	13.8%			9.7%	13.6%		
	LEU	16.0%	21.8%			16.1%	21.6%			16.1%	21.9%			16.1%	21.8%		
	LYS	1.6%	2.3%	12.6%	37.8%	1.7%	2.3%	12.7%	37.4%	1.7%	2.3%	12.8%	38.9%	1.7%	2.3%	12.6%	38.0%
	MET	2.2%	3.0%	17.6%		2.3%	3.0%	17.9%		2.3%	3.1%	18.3%		2.3%	3.1%	18.1%	
	PHE	6.9%	10.9%			6.9%	11.3%			7.0%	11.2%			7.0%	11.1%		
	PRO	5.6%	7.5%			5.5%	7.2%			5.6%	7.4%			5.6%	7.4%		
	SER	8.8%				8.7%				8.7%				8.7%			
	THR	7.0%				7.0%				7.0%				7.0%			
	TRP	2.2%	3.1%			2.2%	3.2%			2.2%	3.2%			2.2%	3.2%		
	TYR	5.0%	7.9%			5.1%	8.1%			5.1%	8.1%			5.1%	8.1%		
	VAL	12.7%				12.6%				12.6%				12.6%			
B		Amino acids contributions to errors at medium ACC values															
	ARG	6.8%	8.2%	23.2%	51.3%	6.8%	8.2%	23.5%	51.6%	6.9%	8.3%	23.2%	50.7%	7.0%	8.3%	23.7%	51.6%
	ASN	7.0%	9.9%			7.0%	9.8%			7.0%	9.9%			7.0%	9.8%		
	ASP	11.2%	14.2%			11.1%	14.0%			10.9%	13.8%			11.0%	13.8%		
	CYS	0.4%				0.4%				0.4%				0.4%			
	GLN	5.5%	7.0%	19.9%		5.5%	7.1%	20.0%		5.5%	7.1%	20.0%		5.5%	7.1%	19.8%	
	GLU	9.7%	12.0%	31.4%		9.8%	12.1%	31.1%		9.8%	11.7%	31.4%		9.9%	11.9%	31.3%	
	HIS	3.2%	5.1%			3.3%	5.1%			3.3%	5.0%			3.3%	5.0%		
	ILE	3.2%	4.2%			3.2%	4.1%			3.1%	4.2%			3.1%	4.1%		
	LEU	6.2%	7.6%			6.3%	7.6%			6.3%	7.6%			6.3%	7.6%		
	LYS	7.2%	8.7%	22.3%	48.7%	7.2%	8.8%	22.2%	48.4%	7.4%	8.8%	22.0%	49.3%	7.4%	8.7%	21.8%	48.4%
	MET	1.0%	1.2%	3.3%		1.0%	1.2%	3.3%		1.0%	1.3%	3.5%		1.0%	1.3%	3.4%	
	PHE	2.5%	3.5%			2.6%	3.7%			2.6%	3.7%			2.7%	3.7%		
	PRO	9.4%	11.3%			9.1%	10.8%			9.2%	11.0%			9.2%	11.0%		
	SER	8.6%				8.6%				8.6%				8.5%			
	THR	8.2%				8.1%				8.1%				8.0%			
	TRP	1.3%	1.7%			1.4%	1.8%			1.4%	1.9%			1.4%	1.9%		
	TYR	3.9%	5.5%			4.0%	5.7%			4.0%	5.7%			4.0%	5.7%		
	VAL	4.6%				4.6%				4.5%				4.5%			
C		Amino acids contributions to errors at high ACC values															
	ARG	13.1%	13.1%	26.6%	48.5%	13.0%	13.2%	26.5%	48.6%	13.0%	13.1%	26.4%	48.3%	13.1%	13.2%	26.5%	48.5%
	ASN	6.9%	8.4%			7.0%	8.4%			6.9%	8.4%			6.9%	8.4%		
	ASP	9.9%	10.7%			10.0%	10.6%			9.9%	10.6%			9.9%	10.6%		
	CYS	0.0%				0.1%				0.1%				0.1%			
	GLN	7.5%	8.9%	15.9%		7.6%	8.9%	16.0%		7.6%	8.9%	16.0%		7.6%	8.8%	16.1%	
	GLU	16.0%	17.1%	29.6%		16.0%	17.2%	29.6%		16.0%	17.0%	29.8%		16.0%	17.1%	29.7%	
	HIS	3.7%	4.7%			3.7%	4.6%			3.7%	4.7%			3.7%	4.7%		
	ILE	1.7%	1.9%			1.7%	1.9%			1.6%	1.9%			1.6%	1.9%		
	LEU	3.8%	4.1%			3.9%	4.1%			3.9%	4.1%			3.8%	4.1%		
	LYS	16.3%	16.0%	26.3%	51.5%	15.9%	16.1%	26.3%	51.4%	15.9%	15.9%	26.1%	51.7%	15.9%	15.9%	26.0%	51.5%
	MET	0.8%	0.9%	1.6%		0.8%	0.9%	1.7%		0.8%	0.9%	1.7%		0.8%	0.9%	1.7%	
	PHE	2.5%	2.9%			2.5%	2.9%			2.5%	3.0%			2.5%	3.0%		
	PRO	4.8%	5.1%			4.7%	4.9%			4.8%	5.0%			4.8%	5.1%		
	SER	2.8%				2.8%				2.8%				2.8%			
	THR	3.2%				3.2%				3.2%				3.2%			
	TRP	1.4%	1.6%			1.4%	1.7%			1.5%	1.7%			1.5%	1.7%		
	TYR	3.9%	4.7%			3.9%	4.7%			4.0%	4.7%			4.0%	4.8%		
	VAL	1.7%				1.7%				1.6%				1.6%			

Figure S8. Amino acid contribution to errors in side-chain prediction programs based on Chi angles deviation by 30° from original. (A) Amino acid contribution percentage in prediction of Chi angles by FASPR, RASP, SCWRL4 and SCWRL4v at low ACC values. (B) Amino acid contribution percentage in prediction of Chi angles by FASPR, RASP, SCWRL4 and SCWRL4v at medium ACC values. (C) Amino acid contribution percentage in prediction of Chi angles by FASPR, RASP, SCWRL4 and SCWRL4v at high ACC values.
